# Toehold switch based biosensors for sensing the highly trafficked rosewood *Dalbergia maritima*

**DOI:** 10.1016/j.synbio.2022.03.003

**Published:** 2022-03-30

**Authors:** Paul Soudier, Daniel Rodriguez Pinzon, Tristan Reif-Trauttmansdorff, Hassan Hijazi, Maëva Cherrière, Cátia Goncalves Pereira, Doriane Blaise, Maxime Pispisa, Angelyne Saint-Julien, William Hamlet, Melissa Nguevo, Eva Gomes, Sophia Belkhelfa, Anna Niarakis, Manish Kushwaha, Ioana Grigoras

**Affiliations:** a Evry Paris-Saclay 2020 iGEM team, France; b Université Paris-Saclay, INRAE, AgroParisTech, Micalis Institute, 78352, Jouy-en-Josas, France; c Université Paris-Saclay, Univ Evry, Laboratoire Européen de Recherche pour la Polyarthrite Rhumatoïde - Genhotel, Evry, France; d Université Paris-Saclay, Univ Evry, CNRS, CEA, Génomique Métabolique, Evry, France

**Keywords:** Toehold switch, Riboregulator, Biosensor, Rosewood, CITES, Convention on International Trade in Endangered Species of Wild Fauna and Flore, CUHK, Chinese University of Hong Kong, MatK, Maturase K, MEFL, Molecules of Equivalent Fluorescein, MFE, Minimum free energy, RbcL, Ribulose bisphosphate carboxylase large chain, RBS, Ribosome binding site, TrnL-UAA, Transfer RNA Leucine UAA

## Abstract

Nucleic acid sensing is a 3 decades old but still challenging area of application for different biological sub-domains, from pathogen detection to single cell transcriptomics analysis. The many applications of nucleic acid detection and identification are mostly carried out by PCR techniques, sequencing, and their derivatives used at large scale. However, these methods’ limitations on speed, cost, complexity and specificity have motivated the development of innovative detection methods among which nucleic acid biosensing technologies seem promising. Toehold switches are a particular class of RNA sensing devices relying on a conformational switch of secondary structure induced by the pairing of the detected trigger RNA with a *de novo* designed synthetic sensing mRNA molecule. Here we describe a streamlined methodology enabling the development of such a sensor for the RNA-mediated detection of an endangered plant species in a cell-free reaction system. We applied this methodology to help identify the rosewood *Dalbergia maritima,* a highly trafficked wood, whose protection is limited by the capacity of the authorities to distinguish protected logs from other unprotected but related species. The streamlined pipeline presented in this work is a versatile framework enabling cheap and rapid development of new sensors for custom RNA detection.

## 1 Introduction

Nucleic acids are the main form of information-storage molecules in living organisms. They encode in their sequence information relevant for all biological processes, the sequence being highly specific to an organism (such as the ribosomal RNA) [1] or to a biological process (such as transcriptomic data) [2]. These features make nucleic acids excellent markers to detect and monitor biological information of interest. Qualitative or quantitative detection of nucleic acid molecules have been used since the invention of PCR in 1986 [3] for applications in fundamental research [2], health, (including pathogens detections [4] and other human disease diagnostic [5], industry [6], forensic science [7], environment [8] and others). Most of these applications utilise the Polymerase Chain Reaction (PCR) technique or its derivatives (RT-PCR, qPCR, RNA-seq, …), sometimes coupled with sequencing approaches to highlight with various degree of quantitativeness and specificity the presence of these nucleic acid markers. Nevertheless, all these techniques have their limitations (specificity, time, cost, need for trained operators or heavy equipment) and the development of tools targeting all these limitations remains challenging [9]. The problem of rapid and inexpensive detection of nucleic acid has been targeted by synthetic biology, a science that aims to apply engineering principles to biological systems to create new parts, devices or systems. In the field of molecular detection, synthetic biology aims to create biosensors which are devices harnessing the diversity of life's biochemical interactions to generate highly specific molecular sensing objects [10]**.**

Using the potential of biomolecular devices naturally interacting with nucleic acid, several biosensors have been developed, in particular for the programmable detection of specific RNA molecules [[11], [12], [13], [14], [15]]. These RNA biosensors can be divided in 2 categories: *in vivo* sensing devices where the aim is to follow the endogenous production of an RNA by a cell equipped with a sensing system, possibly to trigger or control other cellular response in reaction to that sensing [16], and *in vitro* sensing devices where the biosensor is implemented in a membraneless system (e.g. a cell-free system) which allows the detection of exogenous RNA in a given sample [17].

**Fig. 1:**
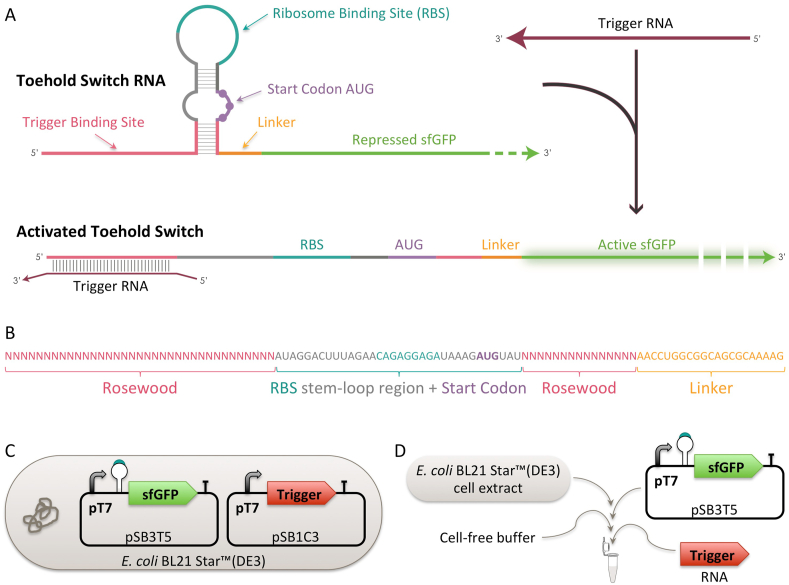
Rosewood toehold switches principle. A. Hairpin structure of the sensor mRNA before and after the binding of trigger RNA. B. Detail of the toehold switch sequence showing conserved and variable regions between different sensor candidates. C/D. *in vivo* (C) and *in vitro* (D) toehold switch activity screening systems.

Toehold switches [13] are a particular class of RNA sensing devices using predictive RNA-RNA pairing interaction to modify a reporter mRNA secondary structure in response to a trigger RNA in order to provide a specific fast and high signal detection of the trigger RNA (Fig. 1A). The binding between the trigger and the switch is an RNA–based device containing a ribosome binding site (RBS) and an AUG start codon embedded in a hairpin structure that blocks translation initiation [13]. The hairpin can be unfolded upon binding of a trigger RNA, dictated by RNA-RNA interactions between the complementary base pairs, thereby exposing the RBS and allowing translation. In the past, such biosensors have been employed to identify relevant biological material and mutations specific to diseases like cancer [18], Zika virus [17], Norovirus [19] or more recently dengue virus [20] and coronaviruses [[21], [22], [23]]. Computer-aided predictions of RNA secondary structures based on thermodynamic models are used to compute sensing devices candidates for any RNA of interest [24,25]. Here we show how these principles can be used to develop a new RNA biosensor detecting specifically the genetic signature of a species of interest. The pipeline we showcase covers all the steps required to achieve the development of a good detection tool for a new species from the identification of a specific nucleic acid signature to the computational design, *in vivo* screening and *in vitro* implementation of the sensing device. We applied that methodology for the detection of Rosewood.

Rosewood is the most trafficked product in the world by volume and value [[26], [27], [28], [29]] far beyond well-known examples like elephant's ivory, rhinoceros' horn or tiger's fur. Several timber species are sold on the market as rosewood: *Dalbergia* spp., *Pterocarpus* spp., *Millettia* spp., … They all have the particularity of having red hardwood. Differences are at various levels like wood color, density, hardness, tree size. Among them *Dalbergia nigra* is threatened with extinction and thus its trade is prohibited by being included in the CITES (Convention on International Trade in Endangered Species of Wild Fauna and Flore) Appendix I. All other *Dalbergia* spp. are included in Appendix II of CITES which list the species who's trade must be controlled in order to avoid their extinction. Timber species that are not listed in CITES are not protected by over-exploitation, however some of them are considered endangered by the International Union for Conservation of Nature's (IUCN) and listed as such in the Red List of Threatened Species. The drawback of this regulation is the increasing demand and market price [30,31] which made this timber subject to illegal trafficking. To stop the illegal rosewood trade, a big part of the difficulty lies in the strenuous, slow identification process [32]. Indeed, once rosewood is logged, it is nearly impossible to distinguish it from other non-protected wood species by naked eye and still hard with the use of a microscope [33]. However, there is compelling scientific evidence that the *Dalbergia* species can be distinguished at the genetic level with a precision that is legally relevant [[34], [35], [36], [37]]. Yet, to the best of our knowledge, no easy-to-use Rosewood identification tool based on genetic signatures exists. Here we developed toehold switch-based biosensors to specifically detect *Dalbergia maritima*, the main rosewood species in Madagascar, an island where the biodiversity of *Dalbergia* spp. is significant [38]. This is a critical step in the development of a low resource nucleic acid biosensor that, coupled with an isothermal amplification step, will provide a highly sensitive and specific assay for recognising this endangered wood species. To the best of our knowledge, this work represents a first example of development of RNA-based synthetic devices for biodiversity protection against illegal trafficking of a wildlife product.

## 2 Materials and methods

### 2.1 *In silico* rosewood toehold switches design

The rosewood toehold switches were designed using the Chinese University of Hong Kong (CUHK) model [25] as detailed in Supplementary Note S1. In order to adopt the previously optimized architecture of the Series B of toehold switch sensors for Zika virus detection [17] and of the BioBits™ toeholds [39], custom Loop and Linker sequences (Fig. 1B) were specified as the parameters in the CUHK web tool (Supplementary Table S1).

The target rosewood sequences were the *Dalbergia maritima* var. *pubescens’* MatK, RbcL and TrnL-UAA genes [36] available in the BOLD database [40] (Acc. numbers listed in Supplementary Table S1).

All designed toehold switches and their parameters are available in Supplementary Tables S2, S3 and S4.

### 2.2 Plasmid construction

The toehold switch sensors were assembled in the low copy plasmid pSB3T5 and placed upstream of sfGFP [41] fused to the LVA degradation tag [42]. The expression was controlled by the T7 promoter and the strong SBa_000587 synthetic terminator [43]. The trigger sequences were placed also under the control of the T7 promoter and followed by the strong SBa_000587 synthetic terminator, but assembled in the high copy plasmid pSB1C3. As positive controls we designed two parts, all harboring the sfGFP-LVAtag under the control of T7 promoter and the strong SBa_000587 synthetic terminator, but with different RBSes: a strong custom made RBS designed for sfGFP-LVAtag using the Salis RBS calculator [44] or the synthetic stem-loop containing an RBS designed by Pardee et al. [17] to be part of a standardized toehold switch which was also used for all our rosewood toehold switches (Fig. 1B and Supplementary Table S1). As negative control, we used BBa_K3453103 in which no promoter and no RBS are present upstream of sfGFP-LVAtag. Full plasmid sequences and their accession numbers are provided as supplementary materials.

### 2.3 Toehold switches *in vivo* characterization

Toehold switches *in vivo* characterization was performed in *E. coli* BL21 Star™(DE3) (Thermo Fisher Scientific) cells which contain a truncated version of the RNaseE gene (*rne131*) that leads to reduced level of mRNA degradation and thus increased RNA stability. *E. coli* cells containing switch and trigger plasmids (Fig. 1C) were first grown overnight in 96-deep-well plates containing 1 mL of LB medium supplemented with 5 μg/mL tetracycline and 17.5 μg/mL chloramphenicol, then diluted by 40x into similar media. Upon reaching early log-phase, cells were further diluted 20x in 100 μL of LB medium supplemented with 5 μg/mL tetracycline, 17.5 μg/mL chloramphenicol and 10 μM IPTG in an opaque wall 96-well polystyrene microplate, the COSTAR 96 (Corning). The plate was incubated at 37 °C at 200 rpm and the sfGFP fluorescence (*λ*_excitation_ 483 nm and *λ*_emission_ 530 nm) and OD_600nm_ were measured every 10 min for 24 h in a CLARIOstar (BMG Labtech GmbH) plate reader. Fluorescence values were normalized by OD_600nm_ and these arbitrary units were converted into Molecules of Equivalent FLuorescein (MEFL)/particle using standard curves prepared as described [[45], [46], [47]].

### 2.4 Toehold switches *in vitro* characterization

An extract based cell-free system was prepared following Noireaux's lab protocol replacing the bead beating step by a french press lysis method [48] using the *E. coli* BL21 Star™(DE3) cells (Thermo Fisher Scientific) grown in 2YTP medium in the presence of 1 mM IPTG for T7 RNA polymerase expression. Mg glutamate, K glutamate and dithiothreitol concentrations were optimized in order to have the highest efficiency of protein production achieved by the cell-free preparation.

Reactions (Fig. 1D) were run in an opaque wall round 384-well polystyrene microplate (Corning) in a final volume of 20 μL constituted by 6.66 μL cell-free extract, 8.33 μL buffer, 5 nM sensor plasmid DNA and 2 μM trigger RNA (produced from a PCR amplified template using the TranscriptAid T7 high yield transcription kit (Thermo Fisher Scientific) according to suppliers instructions). The plate was incubated at 37 °C and the sfGFP fluorescence (*λ*_excitation_ 485 nm and *λ*_emission_ 528 nm) was measured every 5 min for 8 h in a Cytation™3 (BioTek) plate reader. An endpoint measurement was extracted at 2 h and 30 min to compare the fluorescence of the sensor with and without addition of the trigger RNA in a fast output setup. The amount of trigger RNA to be added in the reactions and the endpoint measurement time were determined using the sensitivity tests presented in Supplementary Fig. S3 that show that, after 2 h and 30 min, all reactions reached a quasi steady state after which the fluorescence fold changes were no longer significantly varying. Fluorescence values were converted into MEFL using a calibration curve prepared by adapting the 96-well calibration protocol [45,46] to a 384-well experimental setup.

## 3 Results and discussions

### 3.1 Whole cell screening of rosewood toehold switches

#### *3.1.1* *In vivo* experimental characterization of MatK based rosewood toehold switches

For experimental validation, we selected the top 3 ranked switches for each gene MatK, RbcL and TrnL-UAA and we labeled them 1.1, 1.2, 1.3 in the descending order of the predicted efficacy scores (Supplementary Tables S2, S3 and S4). For MatK, the toehold switches 1.1 and 1.2 are only one nucleotide away, thus we considered that it would be more informative to evaluate the performance of the MatK toehold switch 1.4 instead of 1.2.

**Fig. 2:**
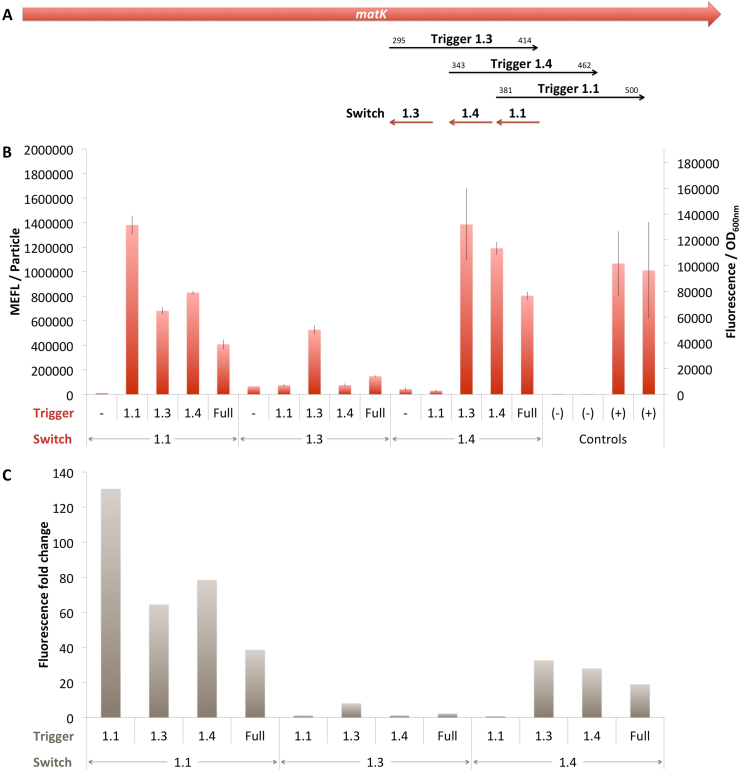
*D. maritima* MatK rosewood toehold switches. A. Schematic representation of the MatK gene and the localisation of switches and triggers. B. *In vivo* characterization of sfGFP expression by *E. coli* BL21 Star™(DE3) cells harboring the DmMatK toehold switches and triggers (Fig. 1C). The negative controls have been performed with an empty pSB3T5, pSB1C3 (no trigger) and BBa_K3453103 (no promoter, no RBS) and the positive controls with BBa_K3453104 and BBa_K3453105. The data and error bars are the mean and standard deviation of at least three measurements on independent biological replicates. C. MEFL/Particle fold changes of the DmMatK toehold switches in the presence of the DmMatK triggers compared to the MEFL/Particle value in the absence of the trigger.

DmMatK 1.1 proved to be one of the best toehold switches (Fig. 2). When tested against the DmMatK 1.1 trigger, Fluorescence/OD_600nm_ reached a value higher than that of the positive controls suggesting that we leveraged the full capacity of the expression cassette (Fig. 2B). In the absence of the trigger this value is very low demonstrating this toehold switch is able to tightly control the translation initiation of the reporter gene. The ON/OFF ratio of this toehold switch was greater than 130, which is on the same order of performance as some of the best switches reported in literature (Fig. 2C). When challenged with the other two DmMatK triggers, the Fluorescence/OD_600nm_ was lower, but the fold change was reasonably high (>60). It is not unexpected that DmMatK 1.3 and 1.4 triggers were able to unfold the DmMatK 1.1 toehold switch. Indeed, as depicted in Fig. 2A, these two triggers overlap with the DmMatK 1.1 and the three of them have the sequence complementary to the DmMatK 1.1 switch sequence. A lower Fluorescence/OD_600nm_, but a reasonably high ∼40 ON/OFF ratio was also observed when the test was performed in the presence of the full known fragments of *D. maritima* MatK gene suggesting this toehold switch is able to be activated by a trigger sequence present in the middle of a long transcript and that the steric constraints do not completely interfere with the switch - trigger binding level.

DmMatK 1.3 is also functional and behaves as expected (Fig. 2). However, when tested against its cognate trigger, this toehold switch showed an ON/OFF ratio of only 8 (Fig. 2C). The main reason is that the Fluorescence/OD_600nm_ reached a value lower than that of the positive controls suggesting that we did not leverage the full capacity of the expression cassette (Fig. 2B). It should be noted that this toehold switch showed to be specific to its cognate trigger. Indeed, when tested with the other two DmMatK triggers that do not contain the DmMatK 1.3 binding site (Fig. 2A), the Fluorescence/OD_600nm_ were very low, at the same level as in the absence of any trigger (Fig. 2B). Moreover, in the presence of the full known fragments of *D. maritima* MatK gene, this DmMatK 1.3 toehold switch was partially turned ON, which indicates lower levels of switch - trigger binding most probably due to steric constraints.

DmMatK 1.4 has a lower predicted efficacy score (Supplementary Table S2), but it behaves better than DmMatK 1.3 (Fig. 2). When tested against its cognate trigger, Fluorescence/OD_600nm_ reached a value higher than that of the positive controls. Similarly high values were obtained also with the DmMatK 1.4 trigger that contains the DmMatK 1.3 binding site, but not with DmMatK 1.1 trigger, nor in the absence of any trigger, thus demonstrating its specificity. In addition, this toehold switch behaved as expected in the presence of the full known fragments of *D. maritima* MatK gene. However, the fluorescence fold changes were lower compared to those observed for the DmMatK 1.1 toehold switch, but still decently high (>30) and this is due to an almost 5 times higher negative output.

#### 3.1.2 *In vivo* experimental characterization of RbcL based rosewood toehold switches

**Fig. 3:**
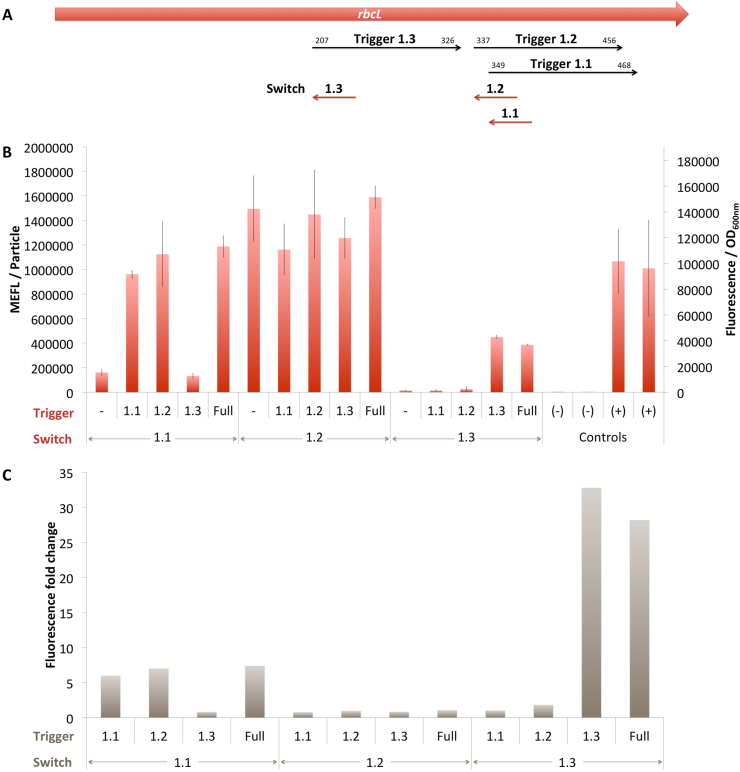
*D. maritima* RbcL rosewood toehold switches. A. Schematic representation of the RbcL gene and the localisation of switches and triggers. B. *In vivo* characterization of sfGFP expression by *E. coli* BL21 Star™(DE3) cells harboring the DmRbcL toehold switches and triggers (Fig. 1C). The negative controls have been performed with an empty pSB3T5, pSB1C3 (no trigger) and BBa_K3453103 (no promoter, no RBS) and the positive controls with BBa_K3453104 and BBa_K3453105. The data and error bars are the mean and standard deviation of at least three measurements on independent biological replicates. C. MEFL/Particle fold changes of the DmRbcL toehold switches in the presence of the DmRbcL triggers compared to the MEFL/Particle value in the absence of the trigger.

DmRbcL 1.1 toehold switch is functional and behaves as expected (Fig. 3). When tested against its cognate trigger, this toehold switch showed fluorescence with a mean comparable to that of the positive controls (Fig. 3B) suggesting that we leveraged the full capacity of the expression cassette. However, its ON/OFF ratio was slightly greater than 5 (Fig. 3C), due to some leakage observed in the absence of the trigger. Nevertheless, the DmRbcL 1.1 toehold switch showed great specificity: when tested with the other DmRbcL triggers, the sfGFP expression was only detected when the trigger contained the switch binding site (Fig. 3B) i.e in the presence of DmRbcL 1.2 and the full known fragments of *D. maritima* RbcL gene, but not in the presence of DmRbcL 1.3 (Fig. 3A). Moreover, with these 2 triggers, the Fluorescence/OD_600nm_ reached values comparable to those obtained in the presence of the cognate trigger, suggesting that for this toehold switch the position of the binding site in the trigger has no influence on its capacity to interact with the switch.

DmRbcL 1.2 toehold switch is not functional and does not behave as expected (Fig. 3). Indeed, a strong sfGFP expression was observed when tested in the no trigger condition, but also in the presence of DmRbcL triggers 1.1 and 1.3 that do not have the cognate binding site. This leaky behavior disqualifies this toehold switch for further consideration.

DmRbcL 1.3 has the lowest predicted efficacy score among all nine selected rosewood toehold switches (Supplementary Table S3), but it behaves correctly (Fig. 3). When tested against its cognate trigger, Fluorescence/OD_600nm_ reached a moderate value of nearly half of that of the positive controls which corresponds nevertheless to a ON/OFF ratio of >30. This same level is attained in the presence of the full known fragments of *D. maritima* RbcL gene suggesting the trigger does not adopt tight secondary structures that may impact its capacity to interact with this toehold switch. A similar behavior was observed with the DmRbcL 1.1. Moreover, the specificity of this toehold switch is obvious from his behavior in the no trigger condition or in the presence of both DmRbcL 1.1 and DmRbcL 1.2 triggers that both do not contain the DmRbcL 1.3 binding site.

#### 3.1.3 *In vivo* experimental characterization of TrnL-UAA based rosewood toehold switches

**Fig. 4:**
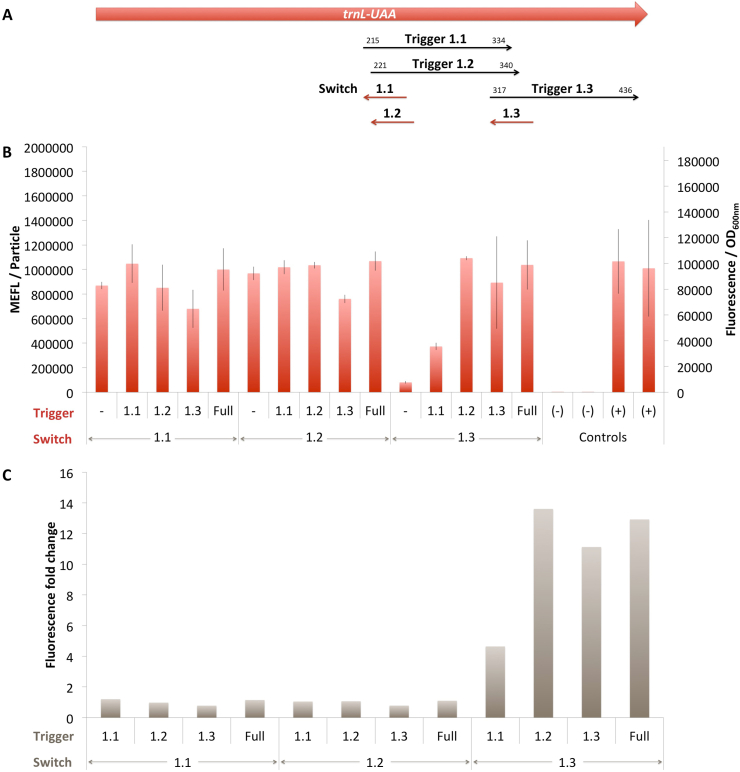
*D. maritima* TrnL-UAA rosewood toehold switches. A. Schematic representation of the TrnL-UAA gene and the localisation of switches and triggers. B. *In vivo* characterization of sfGFP expression by *E. coli* BL21 Star™(DE3) cells harboring the DmTrnL-UAA toehold switches and triggers (Fig. 1C). The negative controls have been performed with an empty pSB3T5, pSB1C3 (no trigger) and BBa_K3453103 (no promoter, no RBS) and the positive controls with BBa_K3453104 and BBa_K3453105. The data and error bars are the mean and standard deviation of at least three measurements on independent biological replicates. C. MEFL/Particle fold changes of the DmTrnL-UAA toehold switches in the presence of the DmTrnL-UAA triggers compared to the MEFL/Particle value in the absence of the trigger.

The top 2 DmTrnL-UAA toehold switches 1.1 and 1.2 were both not functional (Fig. 4). They showed high Fluorescence/OD_600nm_ values comparable to those of the positive controls in the presence of their non-cognate trigger or even in the absence of any trigger. As for DmRbcL 1.2, this leaky behavior disqualifies these toehold switches for further consideration.

DmTrnL-UAA 1.3 toehold switch is functional and behaves as expected (Fig. 4). When tested against its cognate trigger, this toehold switch showed a Fluorescence/OD_600nm_ value with a mean comparable to that of the positive controls suggesting that we leveraged the full capacity of the expression cassette. The ON/OFF ratio of this toehold switch was greater than 10 and this level was reached also in the presence of the full known fragments of *D. maritima* TrnL-UAA gene. Although not evident from the data in Fig. 4B, this toehold switch is specific to its trigger. Indeed both DmTrnL-UAA toehold switch 1.1 and 1.2 partially overlap with the binding site of this toehold switch (Fig. 4A) which explains the high Fluorescence/OD_600nm_ values obtained in the presence of these two triggers.

### 3.2 Cell-free biosensing platform for rosewood RNA

Cell-free platforms constitute a recent technological improvement with several advantages over cell cultures [49]. They can incorporate the detection capacities of biological sensors without preserving the cells, thus have the possibility of being used outside a lab without the limitations of the policies imposed on genetically modified organisms. Moreover, cell-free systems can be freeze-dried on paper discs or in tubes for storage, then the addition of water will rehydrate the system and revive its functionality. This provides an inexpensive, low-tech, and quick applicability for the end user. Golden examples of cell-free paper-based sensors are the ones developed for *in vitro* diagnostics including Zika [17], Norovirus [19], Ebola [50], to name but a few.

In this new platform, we tested the 6 rosewood toehold switches and triggers (Fig. 1D) that showed to be functional in the screening step performed in the whole cell *E. coli* system.

**Fig. 5:**
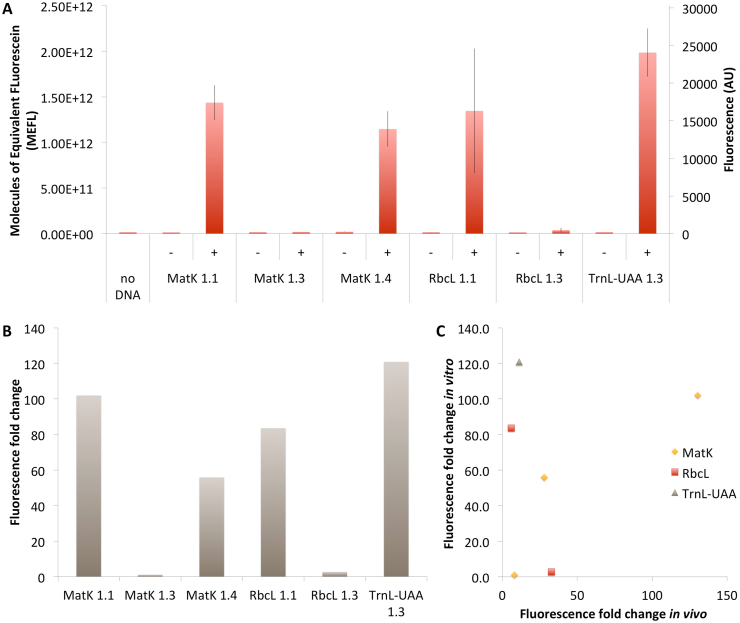
Characterization of sfGFP expression controlled by the rosewood toehold switches and triggers in an *E. coli* BL21 Star™(DE3) based cell-free system (A) and their corresponding fluorescence fold changes (B). The data and error bars are the mean and standard deviation of at least three measurements (row data are available in Supplementary Fig. S7). (C) Comparison of the fold change ratio of the rosewood toehold switches against their cognate triggers measured *in vivo* (Fig.s 2, 3, 4) versus their fold change ratio determined *in vitro*.

The results presented in Fig. 5, show that 4 of the 6 rosewood toehold switches are functional in a cell-free environment and exhibit a significant fluorescent fold change when adding the cognate RNA trigger. Indeed, in the presence of the trigger the fluorescence signal was strongly increased compared to the signal obtained in the absence of the trigger, the fluorescence fold changes ranging from 56 to 121. The highest ON/OFF ratio was exhibited by the toehold switch DmTrnL-UAA 1.3, followed by DmMatK 1.1 and DmRbcL 1.1. The toehold switch DmRbcL 1.3 showed a low positive signal that was large enough to obtain a two fold ON/OFF ratio. Only DmMatK 1.3 shows an indistinguishable ratio of 1 between the positive and negative signals. This toehold switch is thus not functional in cell-free.

Comparing *in vivo* versus cell-free response of the various toehold switch sensor candidates showed on average a low correlation on the ON/OFF fluorescence fold change between these two conditions except for the sensors developed for the gene MatK (Fig. 5C). This finding is coherent with data shown by a recent high throughput study that found similar orders of magnitude fold change and low correlation when transferring toehold switch from *in vivo* to cell-free [51]. On average for the candidates tested here, the results *in vitro* are better than the ones obtained *in vivo*. This may be caused by the difference of physiology between these two systems. Indeed, the cellular composition being more complex than the lysate based cell-free mix, a lot of endogenous nucleic acids only present in the living cell may be interacting with the sensor or the trigger RNA molecules involved in our sensors. Some difference in biochemical conditions such as redox potential or pH may also have an influence on the difference of response found between these systems.

### 3.3 Specificity of rosewood toehold switches

MatK, RbcL and TrnL-UAA are the three genes used in conjunction for DNA barcoding of plant species including *Dalbergia* spp [36].

Six out the nine tested rosewood toehold switches proved to be functional *in vivo* in *E. coli*: DmMatK 1.1 which exhibits an ON/OFF ratio greater than 130, DmMatK 1.4 and RbcL 1.3 which show an ON/OFF level close to 30 and DmMatK 1.3, DmRbcL 1.1 and DmTrnL 1.3 which display at least a level greater than 5. Moreover, these toehold switches performed well in the crosstalk experiments when the expression of the reporter gene was assessed with the trigger of a different toehold switch for the same gene added inside the cell. It should be noted that the triggers are 120 nt long and some contain binding sites for several switches (Fig.s 2A, 3A, 4A). Such triggers are closer to real life situations where the sequence to be detected is part of a long RNA molecule. This allowed us to investigate experimentally their capacity to unfold the corresponding toehold hairpin structure when present at the beginning of the trigger RNA, or embedded at various places in the middle of the RNA.

In addition, three rosewood toehold switches, one for each genetic marker, proved to be functional *in vitro* in an *E. coli*-based cell-free system: DmMatK 1.1 which exhibits an ON/OFF ratio greater than 100, DmRbcL 1.1 which show an ON/OFF level close to 80 and DmTrnL 1.3 which display the highest level greater than 120. To investigate further their capacity for precise identification of our target rosewood species, *D. maritima*, we challenged each of them with triggers issued from the corresponding sequences from other species. For this, we choose several other *Dalbergia* species, as the sequence comparisons (Supplementary Table S5) revealed diversity in the 35 nucleotides of each trigger binding site. We also selected other wood species sold on the market as rosewood, but which trade is not regulated as they are not protected by the CITES regulations: *Acacia excelsa* (ironwood wattle, rosewood), *Acacia melanoxylon* (Australian blackwood), *Berchemia zeyheri* (pink ivory), *Dysoxylum fraserianum* (Australian rose mahogany, rosewood), *Erythroxylum havanense* (redheart), *Machaerium scleroxylon* (pau ferro, Bolivian rosewood), *Metopium brownei* (chechen, Caribbean rosewood), *Millettia laurentii* (wenge, African rosewood) and *Pterocarpus indicus* (narra, Papua New Guinea rosewood).

**Fig. 6:**
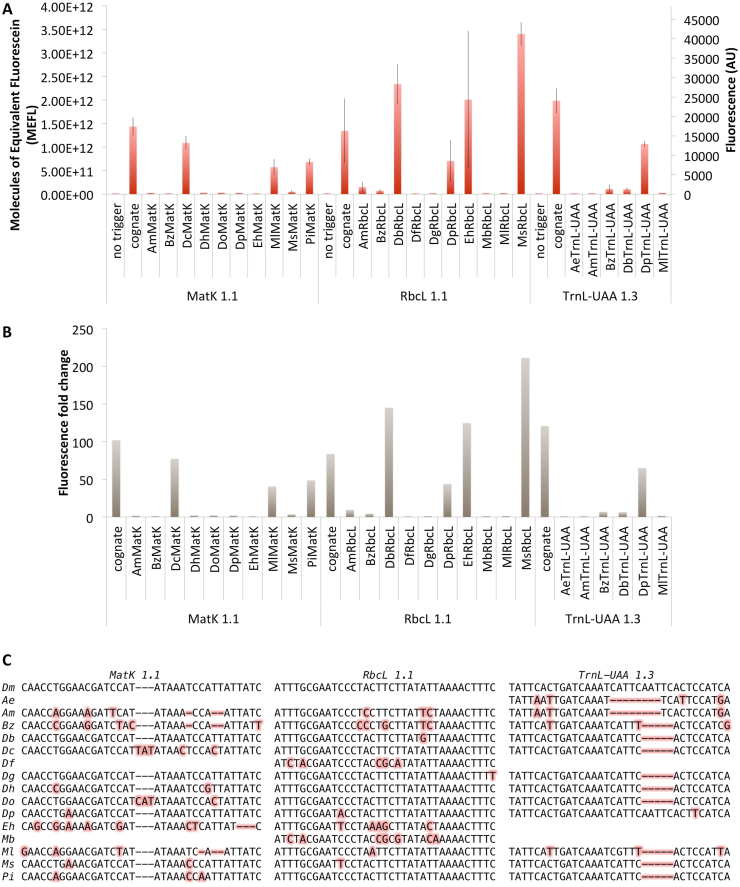
Characterization of sfGFP expression controlled by the rosewood toehold switches and triggers issued from other species in an *E. coli* BL21 Star™(DE3) based cell-free system (A) and their corresponding fluorescence fold changes (B). The data and error bars are the mean and standard deviation of at least three measurements (row data are available in Supplementary Figs. S8, S9 and S10). (C) Sequence comparisons between the DmMatK 1.1, DmRbcL 1.1 and DmTrnL-UAA 1.3 triggers and the corresponding sequences of other species. Polymorphisms are highlighted in pink. Abbreviations before gene names stand for: Ae, *Acacia excelsa*; Am, *Acacia melanoxylon*; Bz, *Berchemia zeyheri*; Db, *Dalbergia baronii*; Dc, *Dalbergia cochinchinensis*; Df, *Dysoxylum fraserianum*; Dg, *Dalbergia granadillo*; Dh, *Dalbergia hupeana*; Dp, *Dalbergia pervillei*; Do, Dalbergia ovata; Eh, *Erythroxylum havanense*; Mb, *Metopium brownei*; Ml, *Millettia laurentii*; Ms, *Machaerium scleroxylon*; Pi, *Pterocarpus indicus.* (For interpretation of the references to color in this figure legend, the reader is referred to the Web version of this article.)

Results presented in Fig. 6 show that the 3 rosewood toehold switches are rather specific to *D. maritima* as they do not allow the expression of the sfGFP reporter gene in the presence of the majority of trigger's variants. However, exceptions occur and this confirms the need already observed that one genetic marker is not enough to distinguish between species. DmMatK 1.1 toehold switch exhibits, apart from its cognate trigger, strong responses in the presence of DcMatK, MlMatK and PiMatK triggers with ON/OFF ratios of 77, 40 and 48 respectively. DmRbcL 1.1 toehold switch is turned ON also in the presence of DbRbcL, DpRbcL, EhRbcL and MsRbcL triggers with ON/OFF ratios of 145, 44, 125 and 211, respectively, and DmTrnL-UAA 1.3 in the presence of DpTrnL-UAA trigger with an ON/OFF ratio of 65. Mild responses (ON/OFF ratio below 10) are also observed in the presence of MsMatK, AmRbcL, BzRbcL, BzTrnL-UAA and DbTrnL-UAA.

All selected triggers have at least one and up to 12 nucleotides mismatches compared to cognate triggers (Fig. 6C). As the binding between the trigger and the switch is dictated by RNA-RNA interactions between the complementary base pairs, increasing the number of mismatches can lead to poor binding and thus to the incapacity of a trigger to unfold the toehold switch hairpin structure.

However, the number of nucleotide mismatches is not the only determining parameter. We observed that the ON/OFF ratio in one example dropped to zero with a single mismatch, while in other cases up to 6 mismatches were tolerated although with lower efficiency (Supplementary Fig. S11A). The structure of the sensor-trigger complex and the kinetic parameters of this interaction play an important role in a toehold switch behavior. To better understand the reasons behind the strong responses observed in the presence of non cognate triggers, we performed secondary-structure predictions of various sensors-triggers pairs using the NUPACK web server [24] (Supplementary Fig. S12 and Table S6).

Looking at the ratio of trigger that was predicted to bind the sensor, we found out that when the binding was predicted absent, the experimental data showed low response. However, for the candidates with high binding proportion predicted, the experimental results were mixed, suggesting that other phenomena such as RNA stability or interaction with other endogenously present RNA may be involved. When comparing the fold change ratios with the free energy of the sensor-trigger structure (Supplementary Fig. S11B), we observed that all pairs having a ΔG°’ value inferior to ∼ −103 kcal/mol have an ON/OFF ratio greater than 60, while all pairs having a ΔG°’ value superior to ∼ −90 kcal/mol are not functional. The pairs having ΔG°’ values between ∼ −90 and ∼ −103 have mixed behaviors, suggesting that the free energy value is not a parameter that can correlate alone with the fold change ratio. These secondary-structure analyses predict interactions between 27 out of 29 sensor-trigger pairs evaluated (Supplementary Fig. S11C and Table S6), however, even though an interaction can occur, the hairpin structure of the toehold switch can still be maintained and thus the translation of the downstream gene is blocked.

Nevertheless, as different species have different combinations of the 3 trigger sequences, an ON response with all three markers concomitantly is obtained in a very limited number of cases (few *Dalbergia* species) and never in the presence of non *Dalbergia* species tested (Supplementary Table S5).

## 4 Conclusions

We have successfully designed, built, tested and analyzed 9 rosewood toehold switches, 6 of which were are able to act as toehold switches that (i) efficiently repress the downstream reporter gene expression in the absence of the cognate trigger and (ii) release the translation inhibition in the presence of the cognate trigger. Moreover, we obtained functional toehold switches for each of the three *D. maritima* genes tested: MatK, RbcL and TrnL-UAA, and further tested them in a cell-free experimental setup as a proof-of-concept for the future implementation of a rosewood detection tool. Thus, we demonstrate the efficiency of 4 rosewood toehold switches in the presence of rosewood RNA both *in vivo* and *in vitro*.

Toehold switches prove once again to be a reliable approach for developing biosensors for the detection of underexplored species based on their nucleic acid signatures. The challenge of the pipeline still resides at the designing step. Indeed, the available tools predicting RNA folding and designing toehold switches have a limited accuracy partially due to external factors such as biological variability, or non-standard physical and chemical conditions. This limited accuracy leads to the inability of certain designed switches to detect the presence of the target RNA at a satisfying level, thus a screening process had to be added to the development pipeline. In this study, we conducted this screening process by testing the toehold candidate plasmids in living *E. coli* cells using *in vivo* produced trigger RNA from a second plasmid. Then these sensors were tested in real conditions (cell-free systems supplemented with trigger RNA) to demonstrate their efficiency. The fold changes reached for these final cell-free sensors are sufficient to distinguish the presence of the trigger RNA in the noisy cell-free environment and are comparable to other fold changes observed for toehold sensors transferred from *in vivo* to *in vitro* [51]. With a sufficient transferability of the whole cell sensor behavior to the cell-free implementation of those, we can envision the use of large libraries of variant screening methods, sorted through flow cytometry to isolate the most promising candidates that can then be used in the cell-free environment. Moreover, finding new toehold switches working *in vivo* is also generally interesting to the synthetic biology and the bio-computing field as they can be integrated in complex genetic circuits as translational regulators to create complexe functions and behaviors [52].

Obtaining a high fold change for a toehold switches in cell-free systems requires a large amount of trigger RNA to demonstrate this behavior and consequently future field implementation of that system will require, in addition to RNA extraction method, the use RNA amplification methods [12] (such as NASBA [11]) to couple with the toehold assay. Indeed, for building such high specificity and high sensitivity sensors, Pardee et al. [17] demonstrated the interest of a 2 steps workflow combining an isothermal amplification step with a toehold switch sensor. The advantage of this method, compared to isothermal amplification only, is a bigger specificity and a lower rate of false positives. As a proof of concept of adapting this framework to the wood species recognition problem, the results presented here demonstrate the efficiency of 4 rosewood toehold switches for 3 different genetic markers commonly used for DNA barcoding of the plant species.

In addition to that, next generations of toehold switch designs following the latest developments in the field could also be considered to improve the developed systems on technical aspects (fold change, dynamic range, specificity …). In particular, the recently published SNIPRs system [18] could allow a single nucleotide resolution in the differentiation of protected wood from their closely related cousins.

As more and more toehold switches are being developed, the available training sets required for artificial intelligence based design systems is thus expanding enabling the deployment of new and more performant toehold switches designing methods and tools [51].

The development of these rosewood biosensors is the first step towards sustainable logging of Rosewood and other endangered species because it represents a high-impact pioneering example of using genomic technologies to maintain biodiversity.

## CRediT authorship contribution statement

**Paul Soudier:** Conceptualization, Methodology, Validation, Formal analysis, Investigation, Writing – original draft, Writing – review & editing, Visualization, Supervision. **Daniel Rodriguez Pinzon:** Conceptualization, Methodology, Validation, Formal analysis, Resources, Writing – original draft, Visualization, Funding acquisition. **Tristan Reif-Trauttmansdorff:** Conceptualization, Methodology, Validation, Formal analysis, Writing – original draft. **Hassan Hijazi:** Conceptualization, Methodology, Validation, Formal analysis. **Maëva Cherrière:** Conceptualization, Investigation, Resources, Funding acquisition, Project administration. **Cátia Goncalves Pereira:** Conceptualization, Investigation. **Doriane Blaise:** Investigation. **Maxime Pispisa:** Conceptualization, Investigation. **Angelyne Saint-Julien:** Conceptualization, Investigation. **William Hamlet:** Conceptualization, Investigation. **Melissa Nguevo:** Conceptualization, Validation, Investigation. **Eva Gomes:** Investigation. **Sophia Belkhelfa:** Investigation, Supervision. **Anna Niarakis:** Conceptualization, Writing – original draft, Writing – review & editing, Supervision. **Manish Kushwaha:** Conceptualization, Methodology, Validation, Formal analysis, Writing – original draft, Writing – review & editing, Supervision. **Ioana Grigoras:** Conceptualization, Methodology, Validation, Formal analysis, Resources, Writing – original draft, Writing – review & editing, Visualization, Supervision, Project administration, Funding acquisition.

## Declaration of competing interest

The authors declare no competing interests.
