# Supplementary material for: Toehold switch based biosensors for sensing the highly trafficked rosewood Dalbergia maritima
Source: Synth Syst Biotechnol. 2022 Mar 30;7(2):791–801. doi: 10.1016/j.synbio.2022.03.003 (PMC8976095; doi:10.1016/j.synbio.2022.03.003)
Supplement: Multimedia component 1 [file mmc1.pdf]

Supplementary information for:

Toehold switch based biosensors for sensing the highly trafficked rosewood *Dalbergia maritima*

Paul Soudier[a,b], Daniel Rodriguez Pinzon[a], Tristan Reif-Trauttmansdorff[a,b], Hassan Hijazi[a], Maëva Cherrière[a], Cátia Goncalves Pereira[a], Doriane Blaise[a], Maxime Pispisa[a], Angelyne Saint-Julien[a], William Hamlet[a], Melissa Nguevo[a], Eva Gomes[a], Sophia Belkhelfa[a], Anna Niarakis[a,c], Manish Kushwaha[a,b] and Ioana Grigoras[a,d,*]

[a] Evry Paris-Saclay 2020 iGEM team
[b] Université Paris-Saclay, INRAe, AgroParisTech, Micalis Institute, Jouy-en-Josas, France.
[c] Université Paris-Saclay, Univ Evry, Laboratoire Européen de Recherche pour la Polyarthrite Rhumatoïde - Genhotel, Evry, France.
[d] Université Paris-Saclay, Univ Evry, CNRS, CEA, Génomique Métabolique, Evry, France.

*Corresponding author:
Ioana Grigoras
ioana.popescu@univ-evry.fr

Table of contents — Page

Supplementary Note S1. *In silico* rosewood toehold switches design. — 3

Supplementary Table S1. CUHK online tool parameters used in this study. — 4

Supplementary Fig. S1. Designed toehold switches for *D. maritima*. — 5

Supplementary Fig. S2. Comparison of the fold change ratio of the rosewood toehold switches against their cognate triggers measured *in vivo* versus their predicted efficacy scores (ON/OFF rates) by the CUHK tool. — 6

Supplementary Fig. S3. Fluorescence fold changes of the DmMatK 1.1, DmRbcL 1.1 and DmTrnL-UAA 1.3 toehold switches over time in the presence of increasing concentrations of their cognate RNA triggers in an *E. coli* BL21 Star™(DE3) based cell-free system. — 7

Supplementary Fig. S4. Time curves of sfGFP expression controlled by the rosewood DmMatK 1.1 toehold switch and increasing concentrations of its cognate RNA trigger in an *E. coli* BL21 Star™(DE3) based cell-free system. — 8

Supplementary Fig. S5. Time curves of sfGFP expression controlled by the rosewood DmRbcL 1.1 toehold switch and increasing concentrations of its cognate RNA trigger in an *E. coli* BL21 Star™(DE3) based cell-free system. — 9

Supplementary Fig. S6. Time curves of sfGFP expression controlled by the rosewood DmMatK 1.1 toehold switch and increasing concentrations of its cognate RNA trigger in an *E. coli* BL21 Star™(DE3) based cell-free system. — 10

Supplementary Fig. S7. Time curves of sfGFP expression controlled by the rosewood toehold switches and cognate triggers in an *E. coli* BL21 Star™(DE3) based cell-free system. — 11

Supplementary Fig. S8. Time curves of sfGFP expression controlled by the rosewood — 15

DmMatK 1.1 toehold switch and various triggers in an *E. coli* BL21 Star™(DE3) based cell-free system.

Supplementary Fig. S9. Time curves of sfGFP expression controlled by the rosewood DmTrnL-UAA 1.3 toehold switch and various triggers in an *E. coli* BL21 Star™(DE3) based cell-free system. **17**

Supplementary Fig. S10. Time curves of sfGFP expression controlled by the rosewood DmMatK 1.1 toehold switch and various triggers in an *E. coli* BL21 Star™(DE3) based cell-free system. **19**

Supplementary Fig. S11. Comparison of the fold change ratio of the rosewood toehold switches against various triggers measured *in vivo* versus their number of nucleotide mismatches compared to cognate triggers, free energy of the trigger-switch dimers secondary structures and the equilibrium concentration of the trigger-switch dimers. **20**

Supplementary Fig. S12. Secondary-structure predictions of various sensors-triggers pairs. **21**

Supplementary Table S7. Sequences used in this study. **34**

Supplementary References **58**

Supplementary Note S1. *In silico* rosewood toehold switches design.

The first step towards the design of rosewood specific biosensors is the *in silico* identification of sequences to be used as targets of toehold switches. However, the genomic data on *Dalbergia* species are very limited: to date, a single draft chromosome-scale [1] and ten full chloroplasts genomes [2,3] have been published. Nevertheless, DNA barcoding studies were previously conducted for conservation purposes [4] and established that the three most common genetic markers for plants, *MatK* (encoding a maturase involved in intron splicing), *RbcL* (encoding the large subunit of Rubisco) and *TrnL-UAA* (a transfer RNA gene) [5] allow reliable distinction between *Dalbergia* species.
To demonstrate the capacity of toehold switches technology to specifically detect a rosewood genetic signature, we decided to focus our attention on *Dalbergia maritima*, the main rosewood species in Madagascar and one of the most heavily harvested and trafficked [6].
For the design, we choose to adopt the previously optimized architecture of the toehold switch sensors for Zika virus detection [7] that proved its efficiency on other toehold switches too [8]. This architecture comprises custom Loop and Linker sequences (Fig. 1B), which we specified as parameters in the CUHK web tool [9] that we used for designing the rosewood toehold switches (Supplementary Table S1). The CUHK model that takes as an input our target rosewood sequence, chops it into smaller fragments and embeds them each into a fixed switch sequence. Then, the RNAfold algorithm, which predicts an unknown structure from a known sequence, is called to predict minimum free energy (MFE) of trigger, switch, switch-domains and trigger-switch complex. The multiple output switches are then ranked according to an efficacy score calculated with a linear regression model trained on 181 switches and their ON/OFF rate to identify the most promising switch candidates.
The CUHK model returned a list of 50 switches for *D. maritima* MatK (Supplementary Table S2), 34 for *D. maritima* RbcL (Supplementary Table S3) and 46 for *D. maritima* TrnL-UAA (Supplementary Table S4). However, the efficacy score of the toehold switches decreased rapidly (Supplementary Fig. 1A). Finally, for each gene only the top few candidates were selected for experimental validation (Supplementary Fig. 1B).
In the top ranked switches, the minimum free energy (MFE) of the trigger-switch heterodimer is at least 28.1 kcal/ mol lower than the total MFE of trigger and switch monomers (termed MFE difference in the CUHK model output). These three switches also have a high difference between the MFE of trigger-switch dimer and either of the trigger-trigger and the switch-switch dimers suggesting that the activated switch conformation is favored thermodynamically. The MFE difference represents the Gibbs free energy change needed for the RNA:RNA interaction to proceed.
Following these calculations, we expect that the MatK 1.1 and MatK 1.2 will have similar beneficial results and that MatK 1.3 will be useful but with lesser performance. Comparing efficacy scores between genes, it is expected that the toehold switches designed to identify TrnL-UAA will have a much higher performance compared to those designed for RbcL, and similar performance compared to best ranked MatK toehold switches.
The experimental validation of the top 3 ranked switches for each gene revealed an *in vivo* and *in vitro* success rate of 66.7% and 44.4% respectively. Thus, the design strategy and the standard architecture (Fig. 1B) of the Series B of toehold switch sensors for Zika virus detection [7] and of the BioBits™ toeholds [8] demonstrated once again its robustness. However, the efficacy score weakly predicted fluorescent measurements of the toehold switches (Supplementary Fig. S2). In particular, the two designs with highest expected performance *in silico* did not produce functional toehold switches. In addition, the poorest design in terms of efficacy turned out to be the toehold switch with the second highest fold change ratio. These results confirm the challenging nature of predicting RNA secondary structure dynamics *in silico.*

Supplementary Table S1. CUHK online tool parameters used in this study.

Target RNA sequence	*Dalbergia maritima* MatK, RbcL or TrnL-UAA gene fragments:<br>● MatK: BOLD Acc. n° MADA155-14, MADA063-14, MADA062-14, MADA158-14, MADA065-14, MADA104-14, MADA023-14, MADA071-14, MADA110-14<br>● RbcL: BOLD Acc. n° MADA155-14, MADA063-14, MADA062-14, MADA158-14, MADA065-14, MADA104-14, MADA023-14, MADA071-14, MADA110-14<br>● TrnL-UAA (BOLD Acc. n° MADA155-14, MADA063-14, MADA062-14, MADA158-14, MADA065-14, MADA104-14, MADA023-14, MADA071-14, MADA110-14
Loop sequence	Custom: GGACUUUAGAACAGAGGAGAUAAAGAUG
Trigger length	120
Toehold domain length	35
Maximum toehold domain base pairs	5
Working temperature	37°C
Promoter sequence	T7
Linker sequence	AACCUGGCGGCAGCGCAAAAG
RE site for cloning	--

Supplementary Fig. S1. Designed toehold switches for *D. maritima*.

A. Efficacy scores (ON/OFF rates) estimated by CUHK model of the 130 switches designed for *D. maritima*.

B. Secondary structures of the top toehold switches predicted by NUPACK web server [10] using default parameters represented in dot-bracket notation coloured to match the different segments in Fig. 1A.

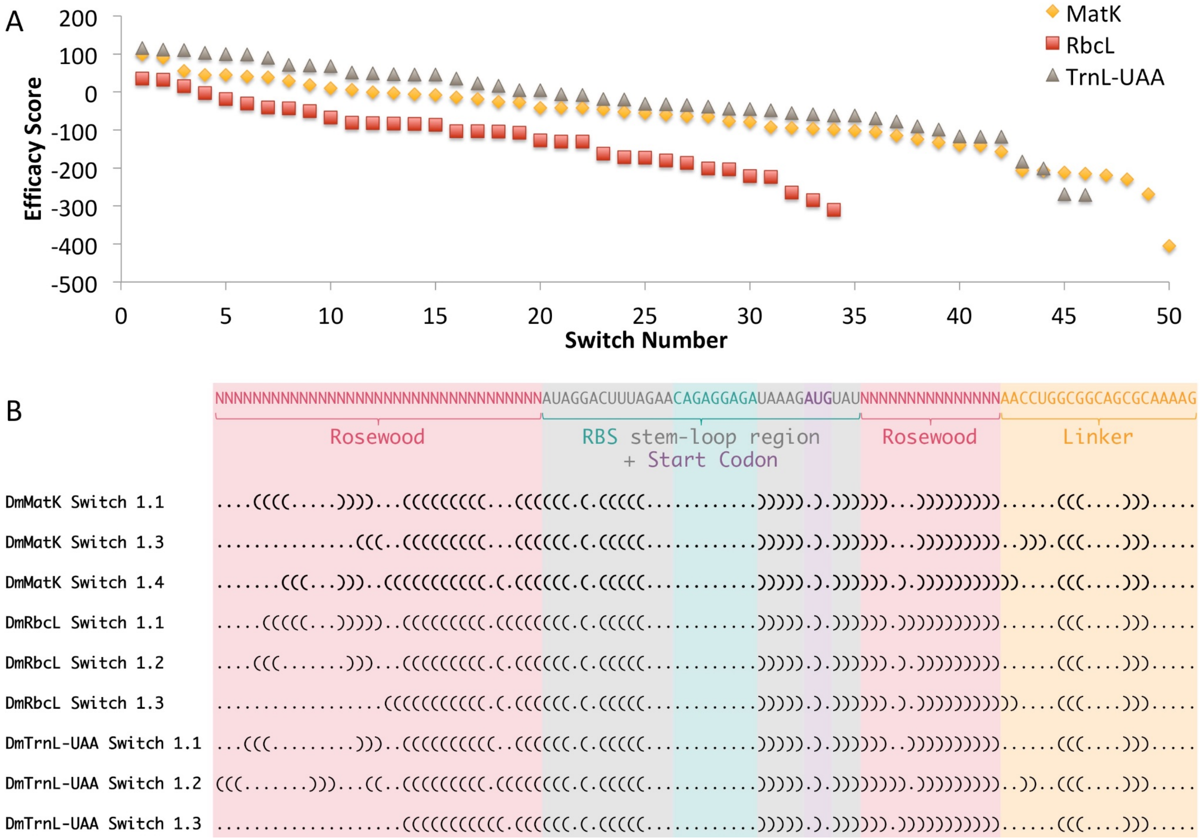

Supplementary Fig. S2. Comparison of the fold change ratio of the rosewood toehold switches against their cognate triggers measured *in vivo* (Fig.s 2, 3, 4) versus their predicted efficacy scores (ON/OFF rates) by the CUHK tool (Supplementary Tables S2, S3 and S4).

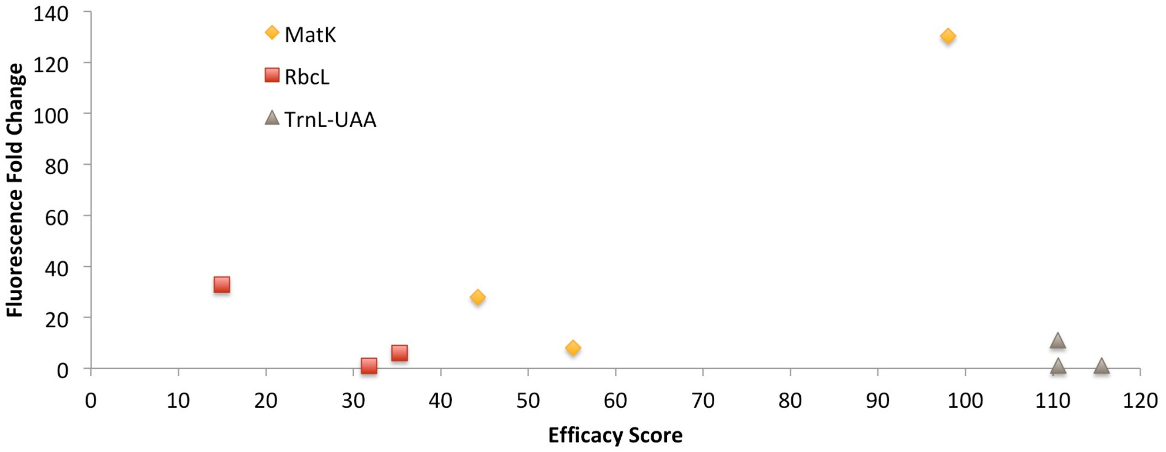

Supplementary Fig. S3. Fluorescence fold changes of the DmMatK 1.1, DmRbcL 1.1 and DmTrnL-UAA 1.3 toehold switches over time in the presence of increasing concentrations of their cognate RNA triggers in an *E. coli* BL21 Star™(DE3) based cell-free system. Row time curves used are presented in Supplementary Fig.s S4, S5 and S6.

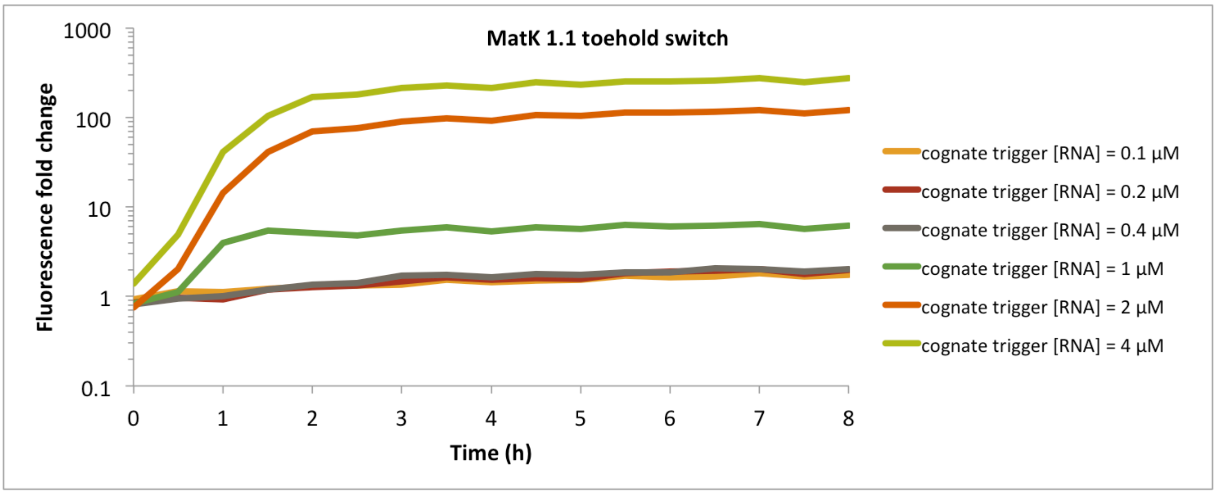

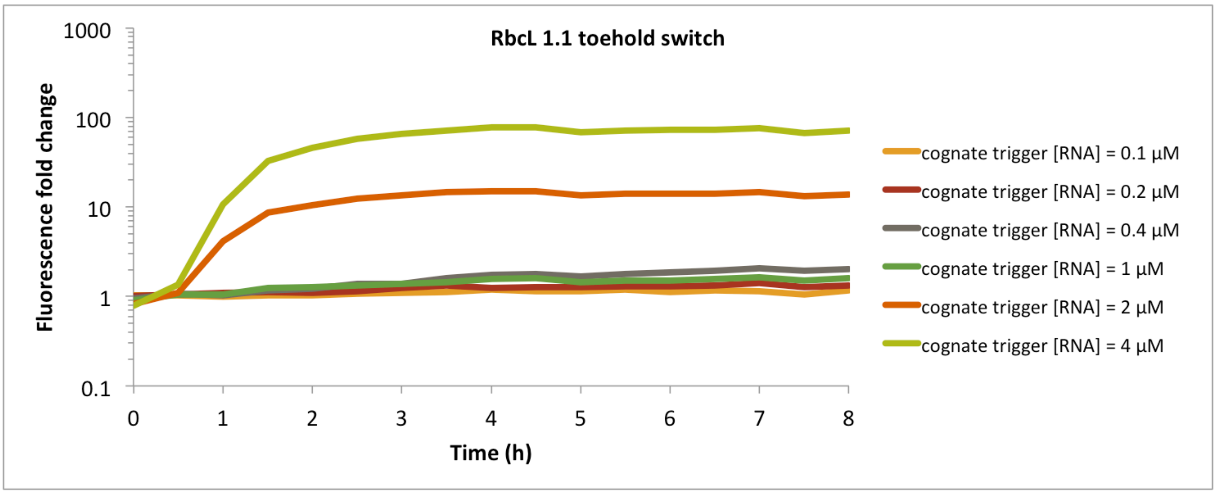

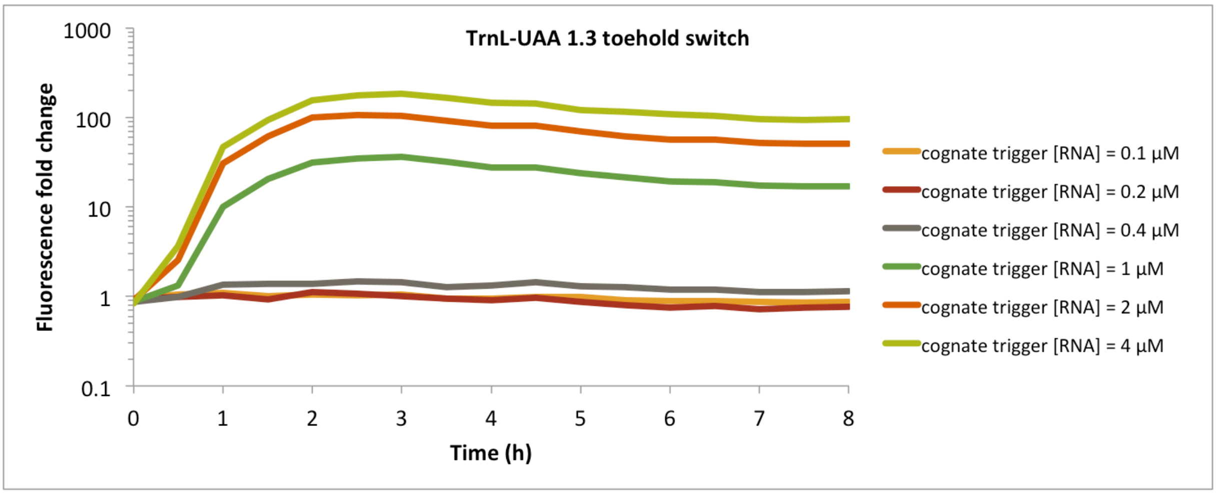

Supplementary Fig. S4. Time curves of sfGFP expression controlled by the rosewood DmMatK 1.1 toehold switch and increasing concentrations of its cognate RNA trigger in an *E. coli* BL21 Star™(DE3) based cell-free system. Measurements correspond to data presented in Supplementary Fig. S3.

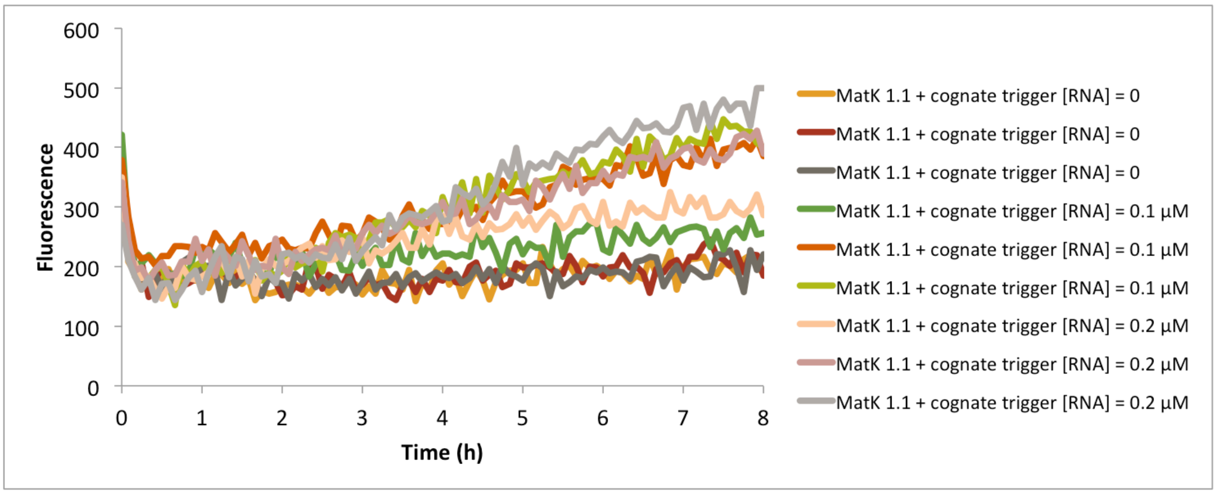

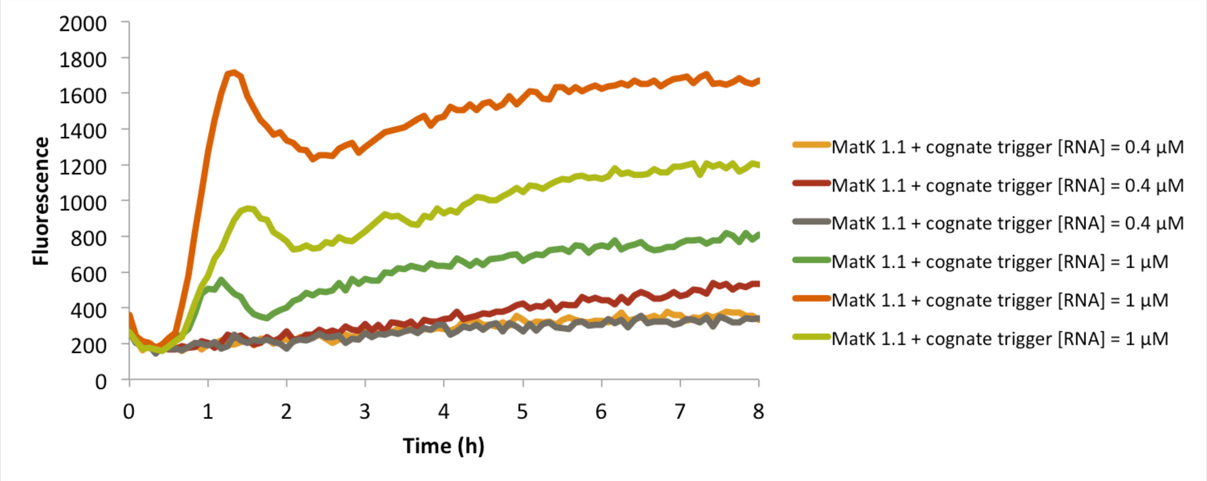

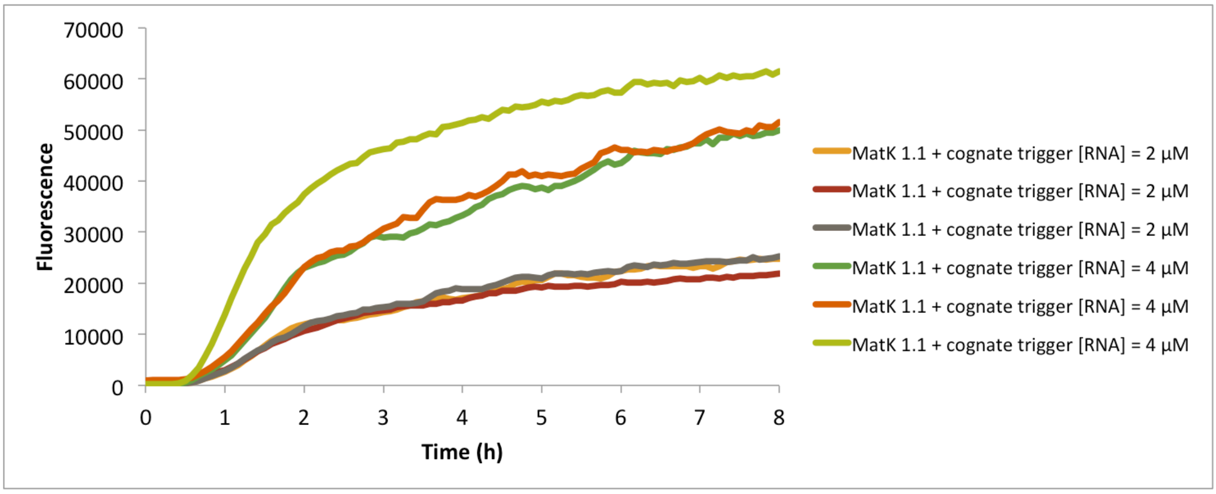

Supplementary Fig. S5. Time curves of sfGFP expression controlled by the rosewood DmRbcL 1.1 toehold switch and increasing concentrations of its cognate RNA trigger in an *E. coli* BL21 Star™(DE3) based cell-free system. Measurements correspond to data presented in Supplementary Fig. S3.

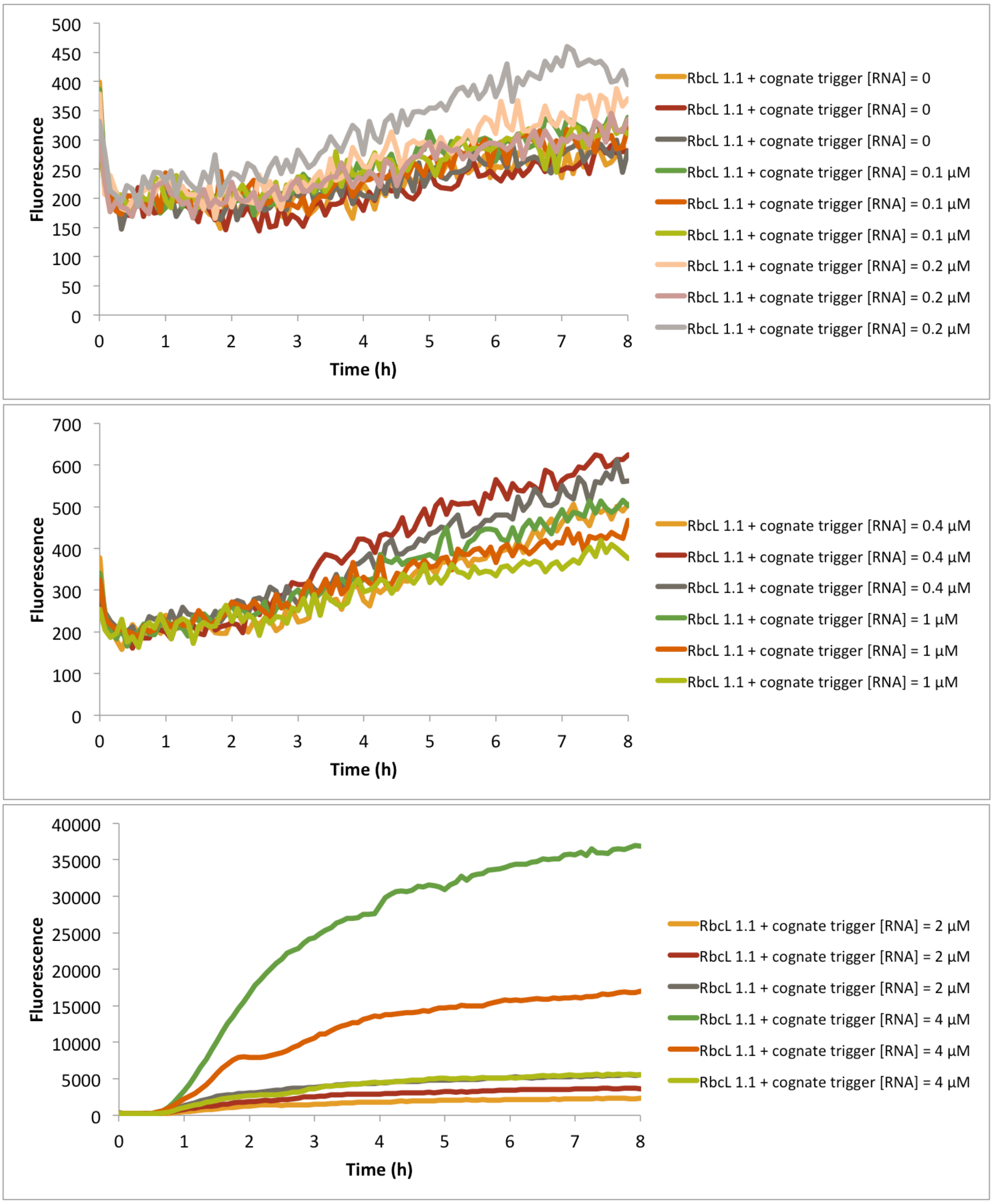

Supplementary Fig. S6. Time curves of sfGFP expression controlled by the rosewood DmTrnL-UAA 1.3 toehold switch and increasing concentrations of its cognate RNA trigger in an *E. coli* BL21 Star™(DE3) based cell-free system. Measurements correspond to data presented in Supplementary Fig. S3.

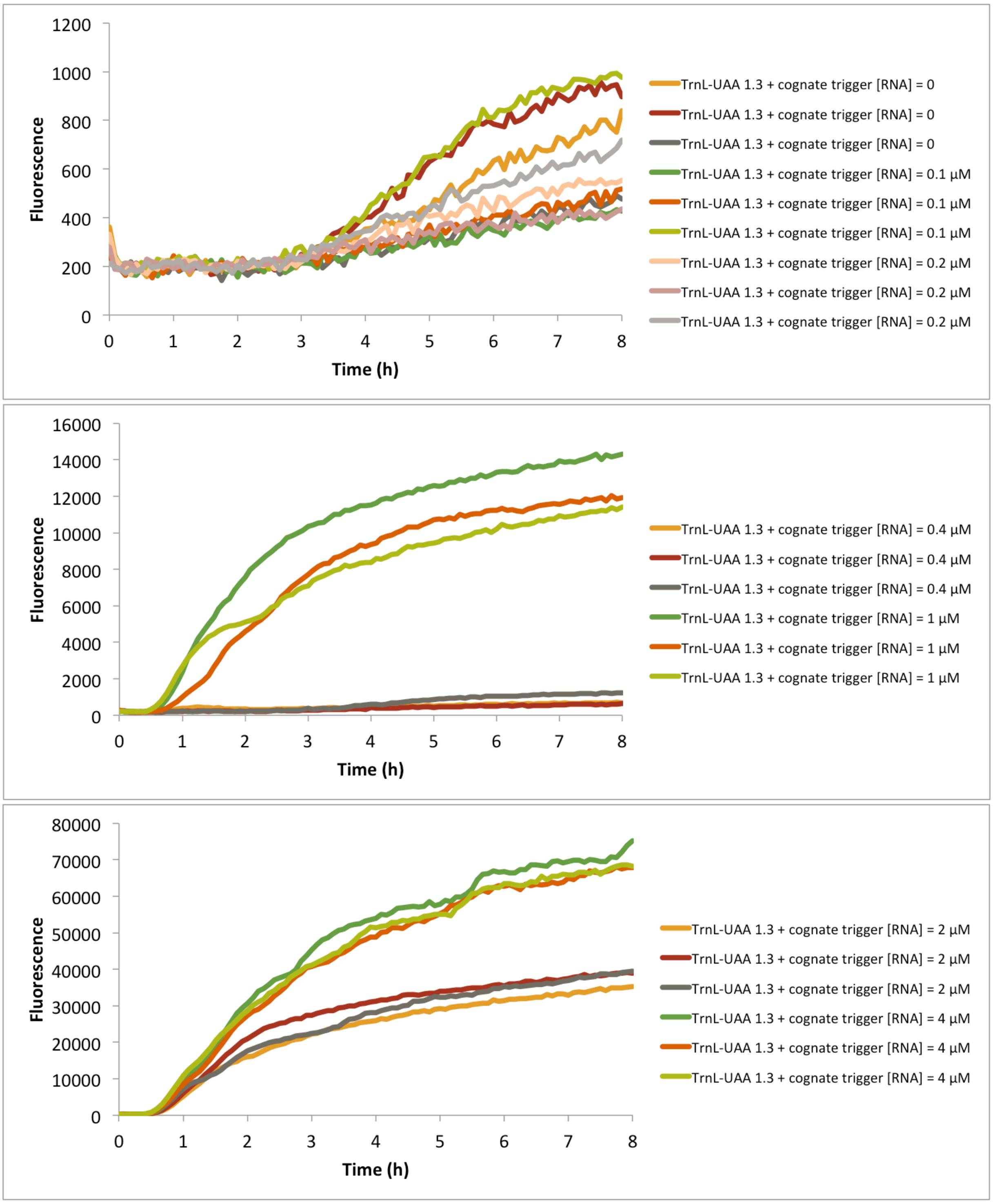

Supplementary Fig. S7. Time curves of sfGFP expression controlled by the rosewood toehold switches and cognate triggers in an *E. coli* BL21 Star™(DE3) based cell-free system. Measurements correspond to data presented in Fig. 6.

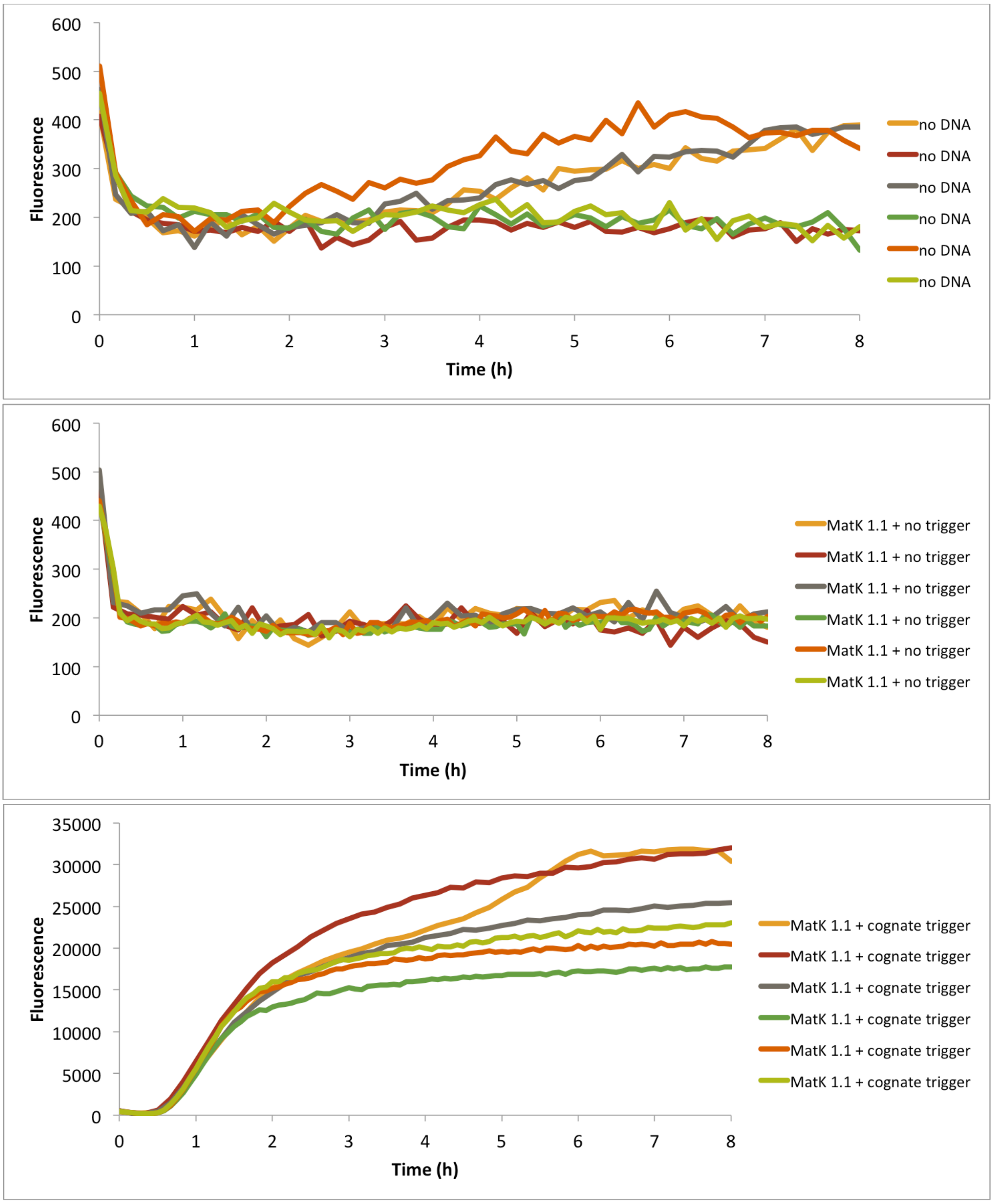

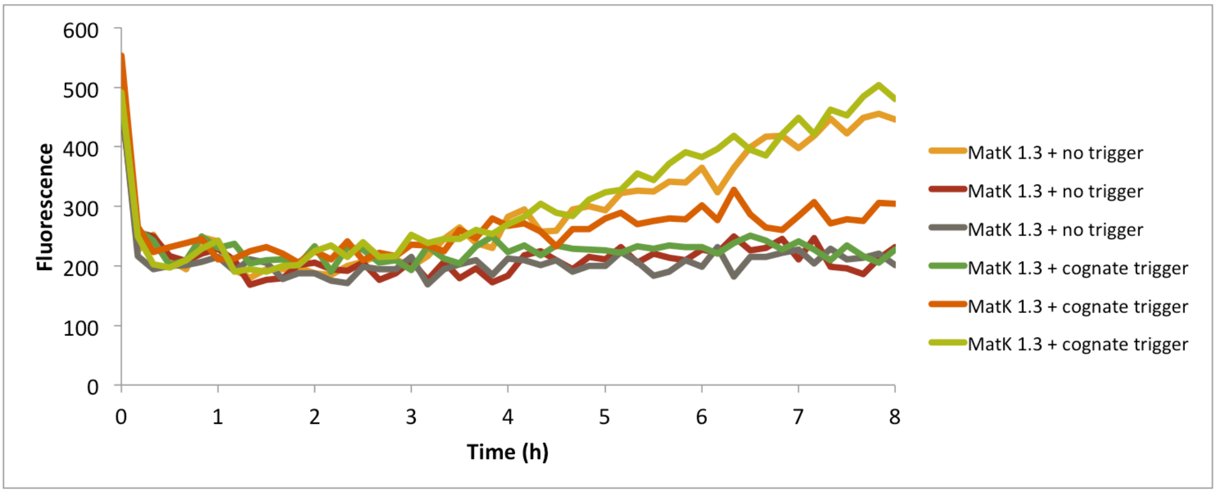

Fluorescence
Time (h)
MatK 1.3 + no trigger
MatK 1.3 + no trigger
MatK 1.3 + no trigger
MatK 1.3 + cognate trigger
MatK 1.3 + cognate trigger
MatK 1.3 + cognate trigger

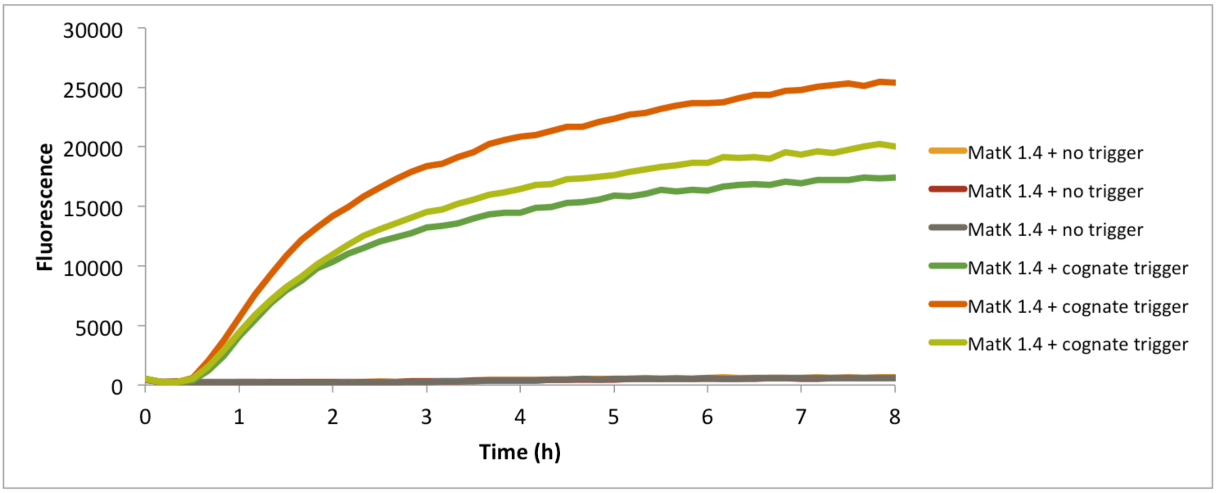

Fluorescence
Time (h)
MatK 1.4 + no trigger
MatK 1.4 + no trigger
MatK 1.4 + no trigger
MatK 1.4 + cognate trigger
MatK 1.4 + cognate trigger
MatK 1.4 + cognate trigger

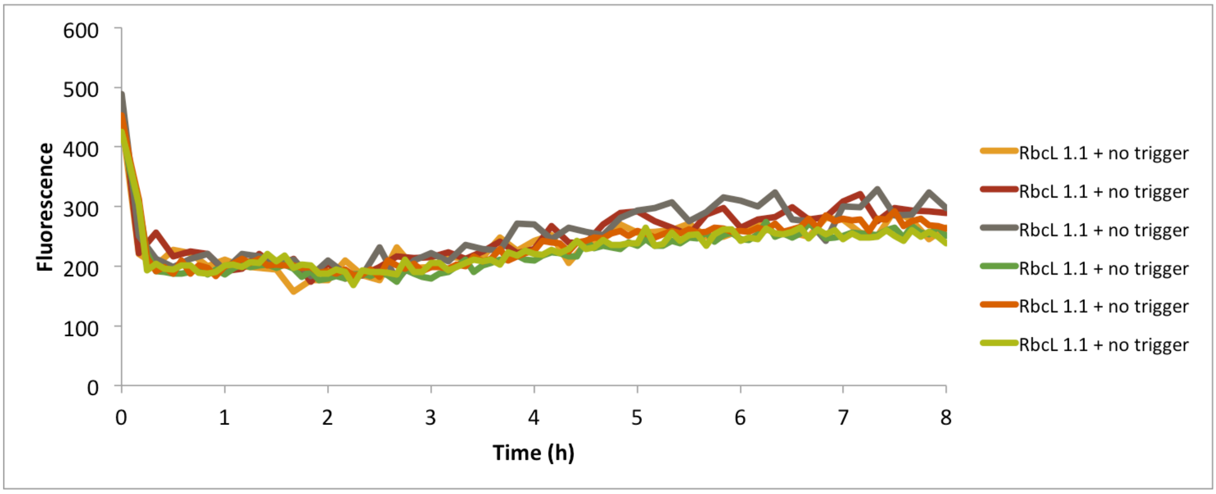

Fluorescence
Time (h)
RbcL 1.1 + no trigger
RbcL 1.1 + no trigger
RbcL 1.1 + no trigger
RbcL 1.1 + no trigger
RbcL 1.1 + no trigger
RbcL 1.1 + no trigger

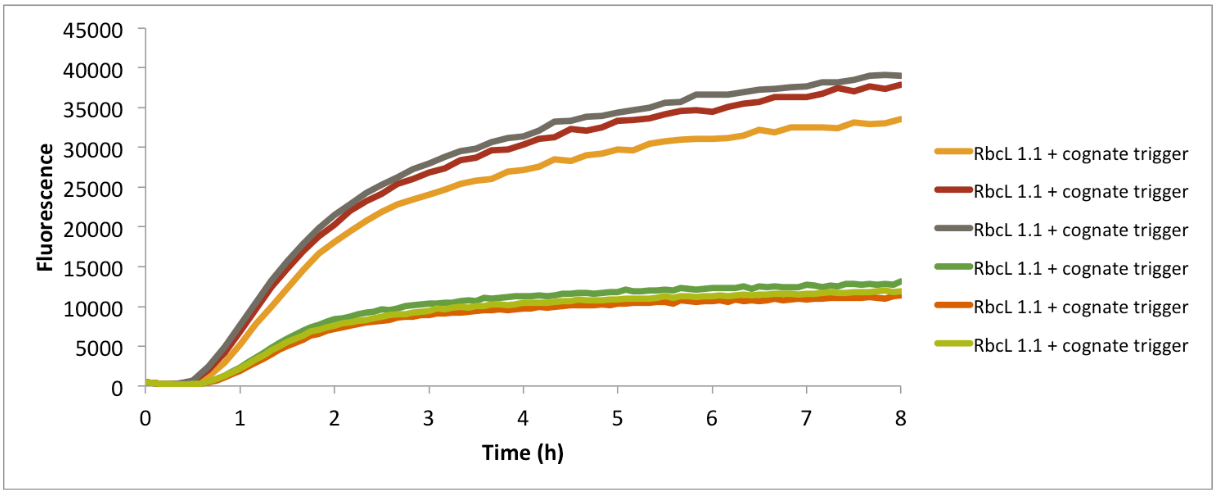
45000
40000
35000
30000
25000
20000
15000
10000
5000
0
Fluorescence
0
1
2
3
4
5
6
7
8
Time (h)
RbcL 1.1 + cognate trigger
RbcL 1.1 + cognate trigger
RbcL 1.1 + cognate trigger
RbcL 1.1 + cognate trigger
RbcL 1.1 + cognate trigger
RbcL 1.1 + cognate trigger

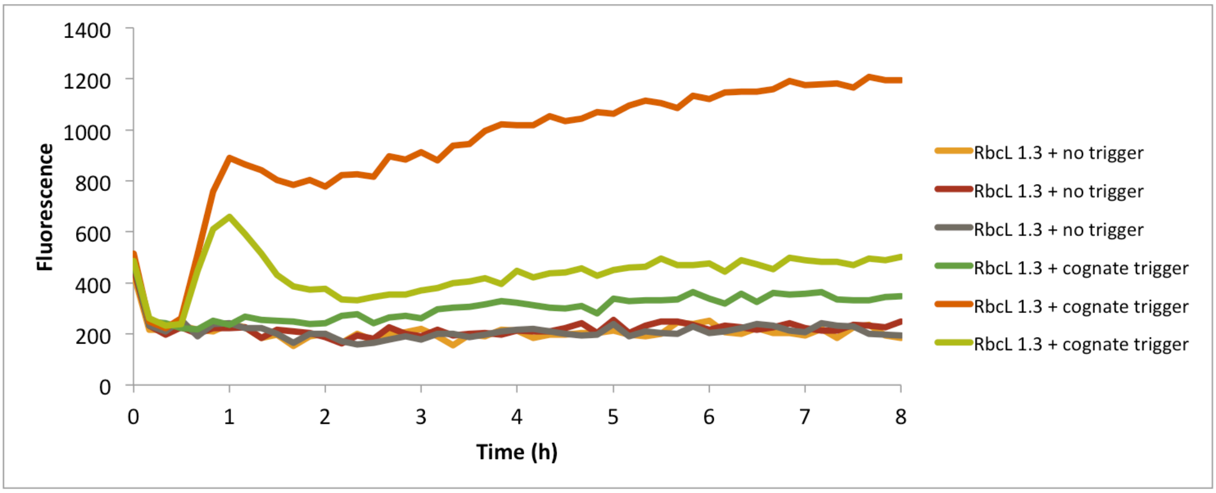
1400
1200
1000
800
600
400
200
0
Fluorescence
0
1
2
3
4
5
6
7
8
Time (h)
RbcL 1.3 + no trigger
RbcL 1.3 + no trigger
RbcL 1.3 + no trigger
RbcL 1.3 + cognate trigger
RbcL 1.3 + cognate trigger
RbcL 1.3 + cognate trigger

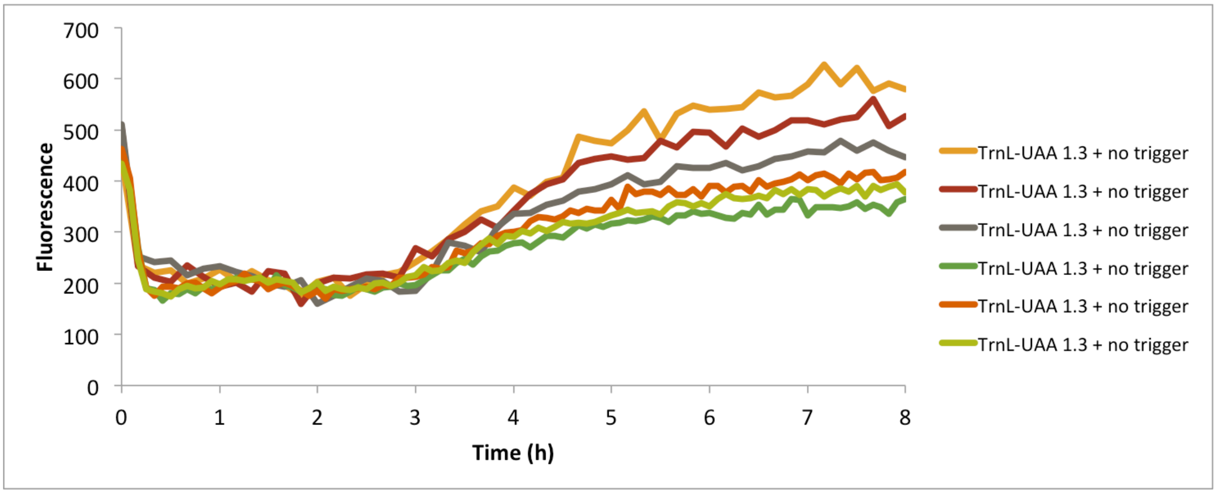
700
600
500
400
300
200
100
0
Fluorescence
0
1
2
3
4
5
6
7
8
Time (h)
TrnL-UAA 1.3 + no trigger
TrnL-UAA 1.3 + no trigger
TrnL-UAA 1.3 + no trigger
TrnL-UAA 1.3 + no trigger
TrnL-UAA 1.3 + no trigger
TrnL-UAA 1.3 + no trigger

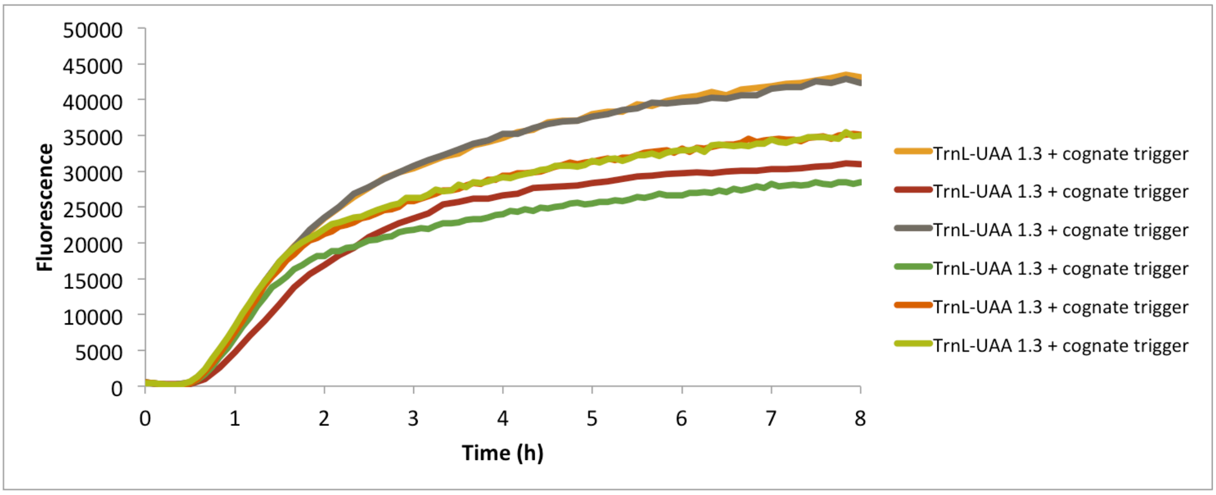
50000
45000
40000
35000
30000
25000
20000
15000
10000
5000
0
Fluorescence
0
1
2
3
4
5
6
7
8
Time (h)
TrnL-UAA 1.3 + cognate trigger
TrnL-UAA 1.3 + cognate trigger
TrnL-UAA 1.3 + cognate trigger
TrnL-UAA 1.3 + cognate trigger
TrnL-UAA 1.3 + cognate trigger
TrnL-UAA 1.3 + cognate trigger

Supplementary Fig. S8. Time curves of sfGFP expression controlled by the rosewood DmMatK 1.1 toehold switch and various triggers in an *E. coli* BL21 Star™(DE3) based cell-free system. Measurements correspond to data presented in Fig. 7.

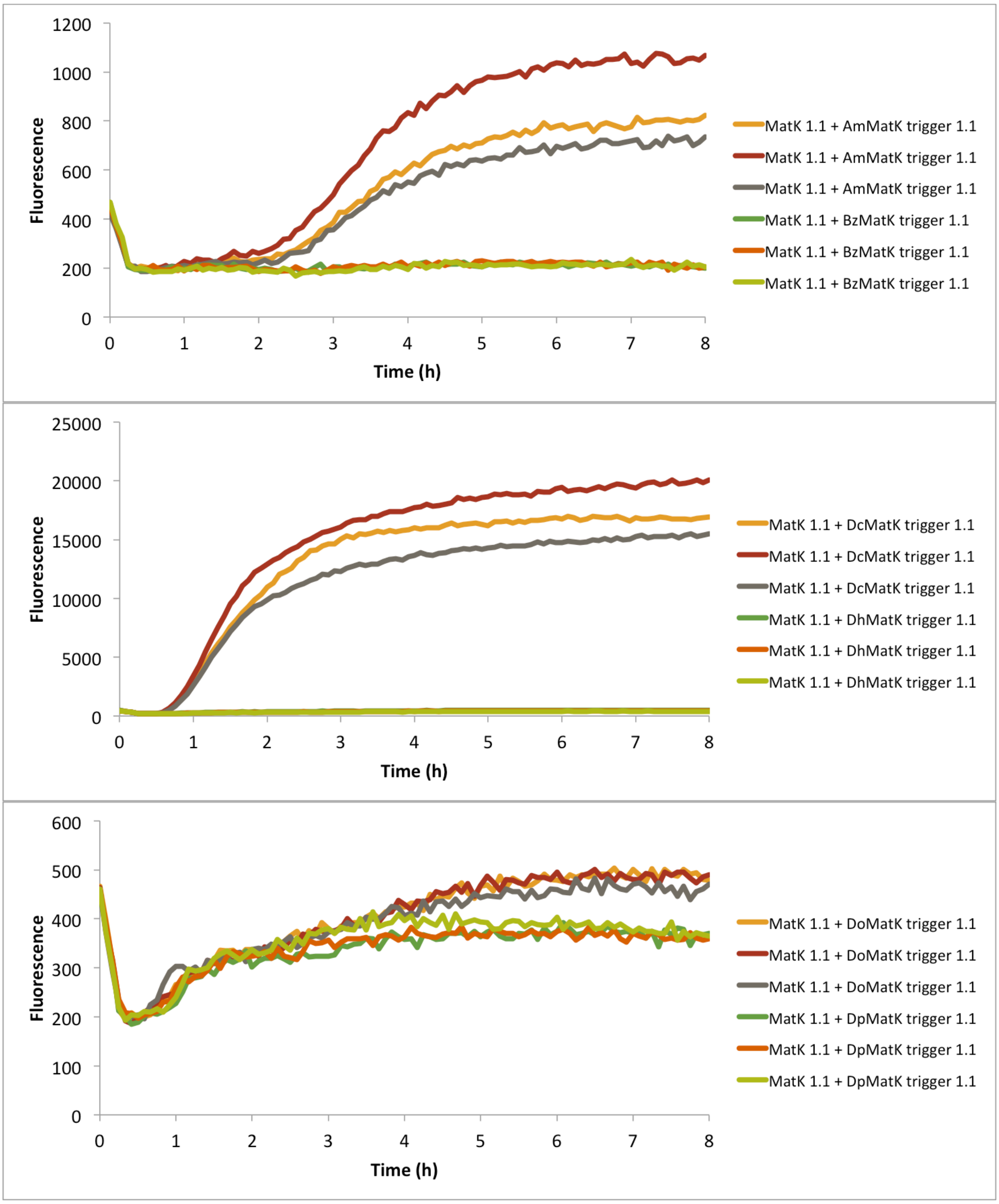

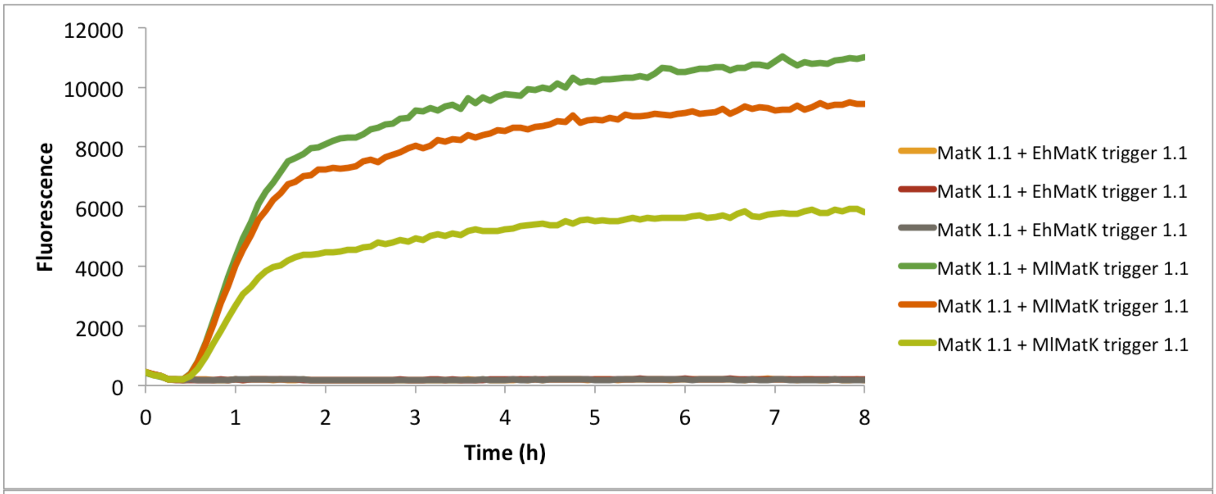

Fluorescence
Time (h)
MatK 1.1 + EhMatK trigger 1.1
MatK 1.1 + EhMatK trigger 1.1
MatK 1.1 + EhMatK trigger 1.1
MatK 1.1 + MlMatK trigger 1.1
MatK 1.1 + MlMatK trigger 1.1
MatK 1.1 + MlMatK trigger 1.1

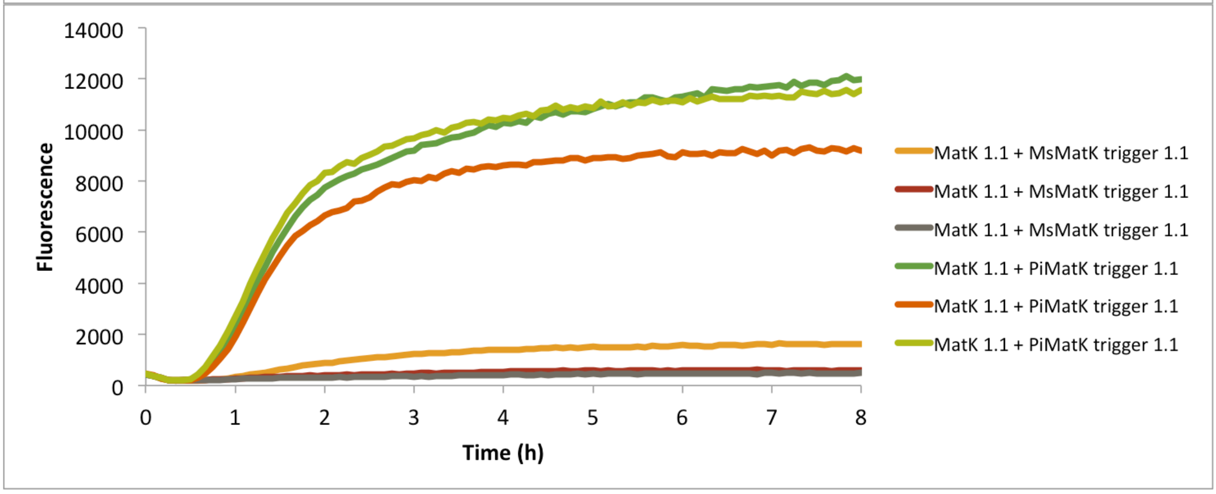

Fluorescence
Time (h)
MatK 1.1 + MsMatK trigger 1.1
MatK 1.1 + MsMatK trigger 1.1
MatK 1.1 + MsMatK trigger 1.1
MatK 1.1 + PiMatK trigger 1.1
MatK 1.1 + PiMatK trigger 1.1
MatK 1.1 + PiMatK trigger 1.1

Supplementary Fig. S9. Time curves of sfGFP expression controlled by the rosewood RbcL 1.1 toehold switch and various triggers in an *E. coli* BL21 Star™(DE3) based cell-free system. Measurements correspond to data presented in Fig. 7.

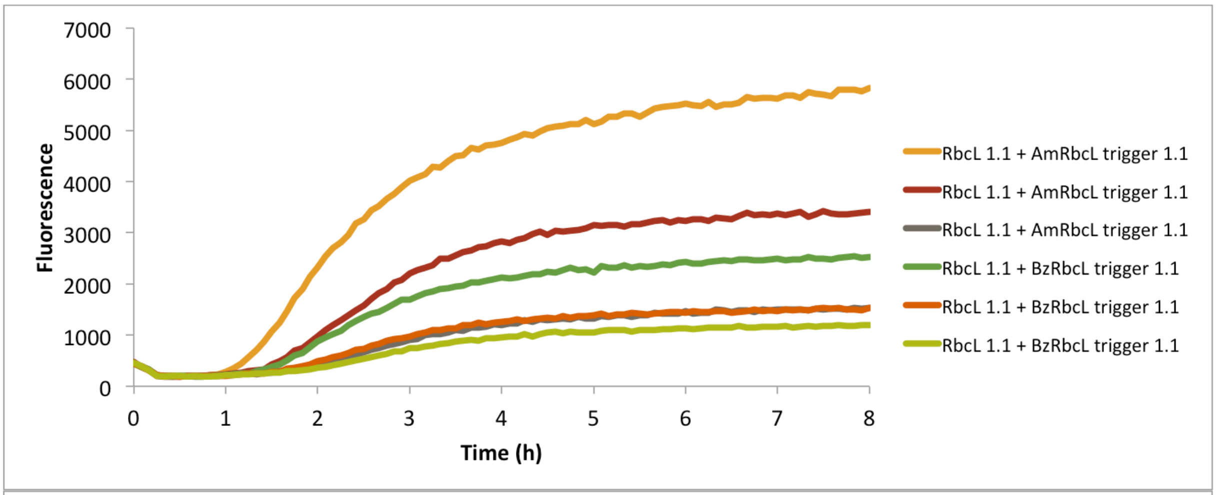

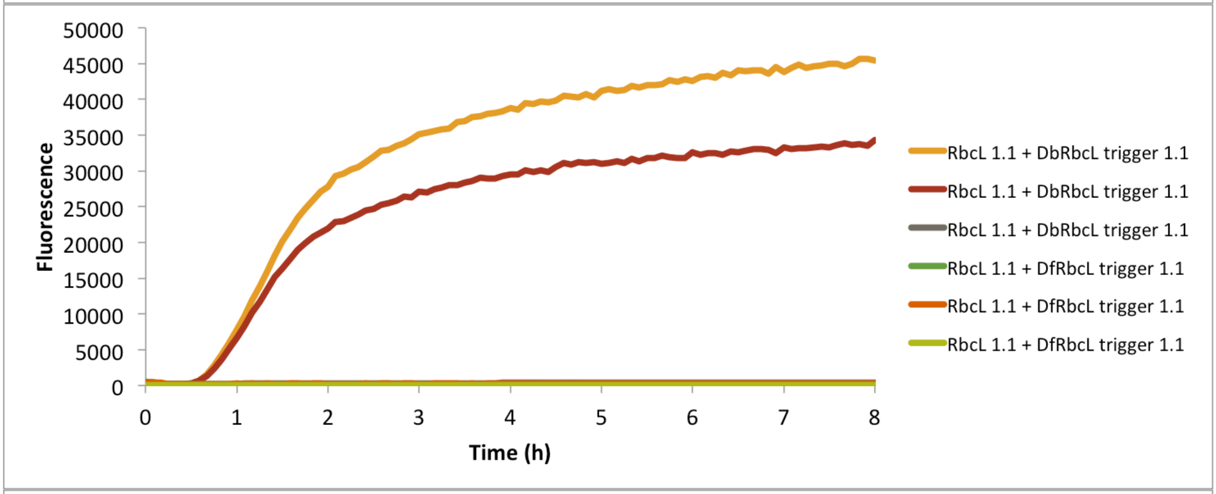

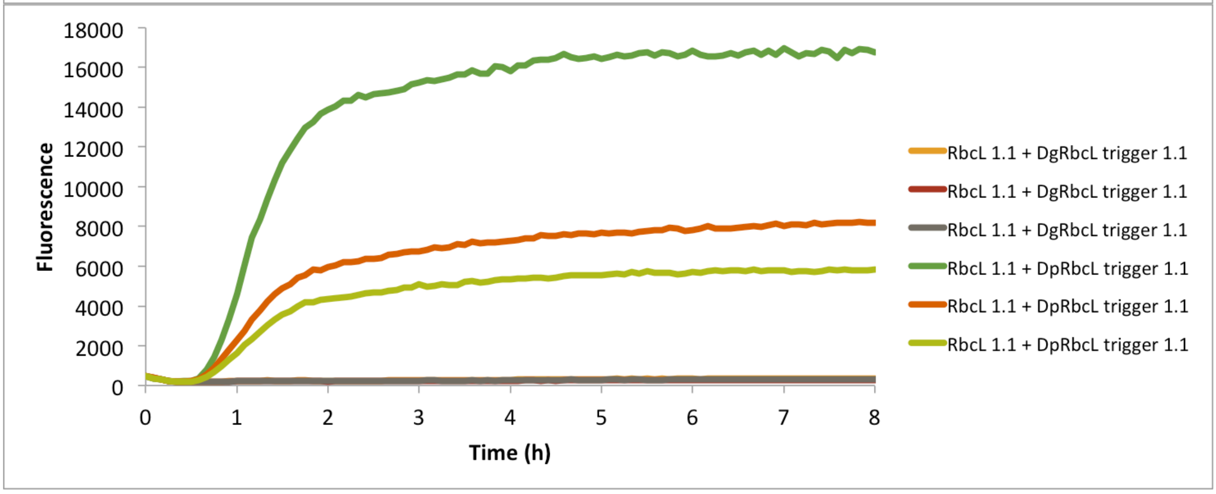

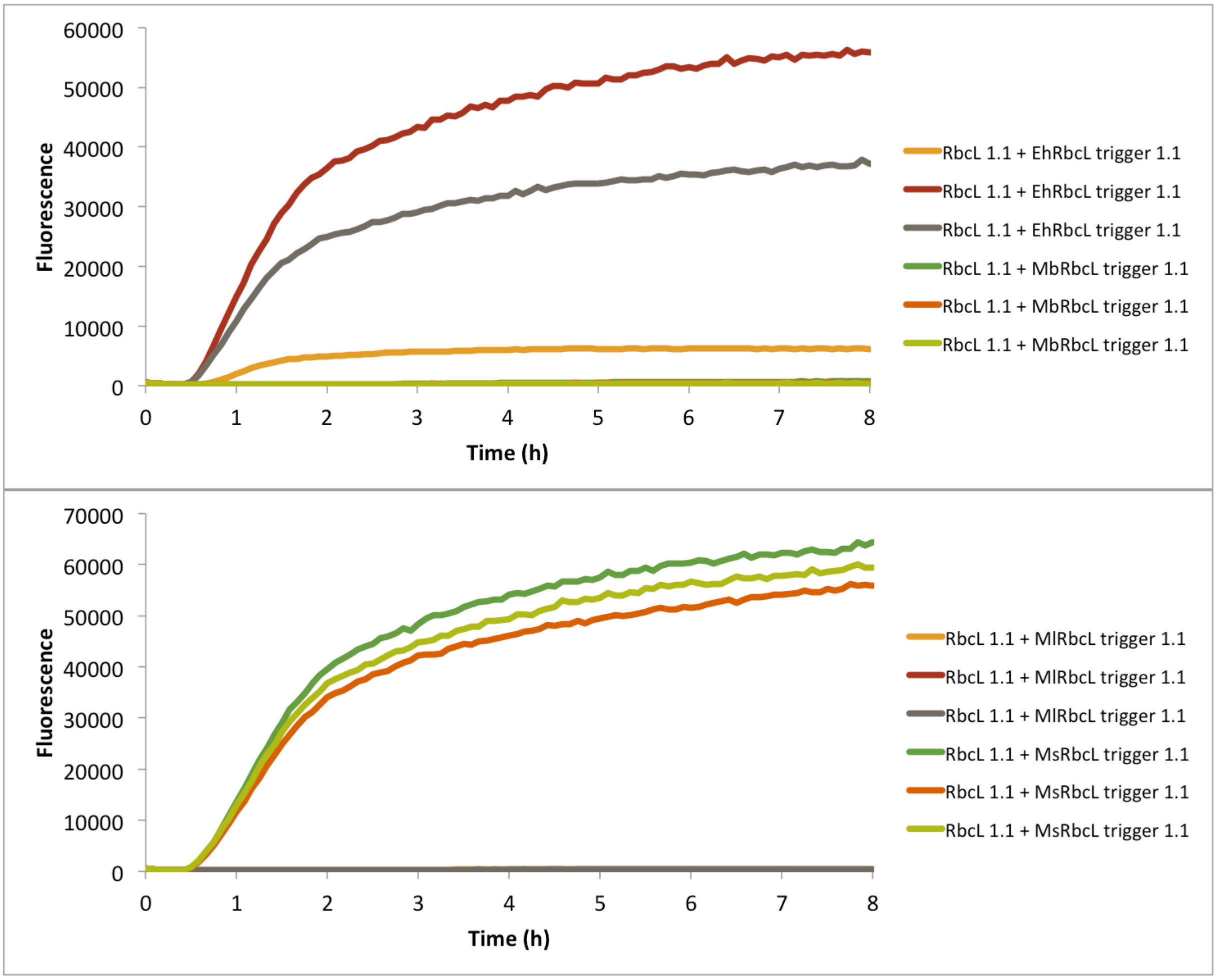

Fluorescence
Time (h)
RbcL 1.1 + EhRbcL trigger 1.1
RbcL 1.1 + EhRbcL trigger 1.1
RbcL 1.1 + EhRbcL trigger 1.1
RbcL 1.1 + MbRbcL trigger 1.1
RbcL 1.1 + MbRbcL trigger 1.1
RbcL 1.1 + MbRbcL trigger 1.1
Fluorescence
Time (h)
RbcL 1.1 + MlRbcL trigger 1.1
RbcL 1.1 + MlRbcL trigger 1.1
RbcL 1.1 + MlRbcL trigger 1.1
RbcL 1.1 + MsRbcL trigger 1.1
RbcL 1.1 + MsRbcL trigger 1.1
RbcL 1.1 + MsRbcL trigger 1.1

Supplementary Fig. S10. Time curves of sfGFP expression controlled by the rosewood DmTrnL-UAA 1.3 toehold switch and various triggers in an *E. coli* BL21 Star™(DE3) based cell-free system. Measurements correspond to data presented in Fig. 7.

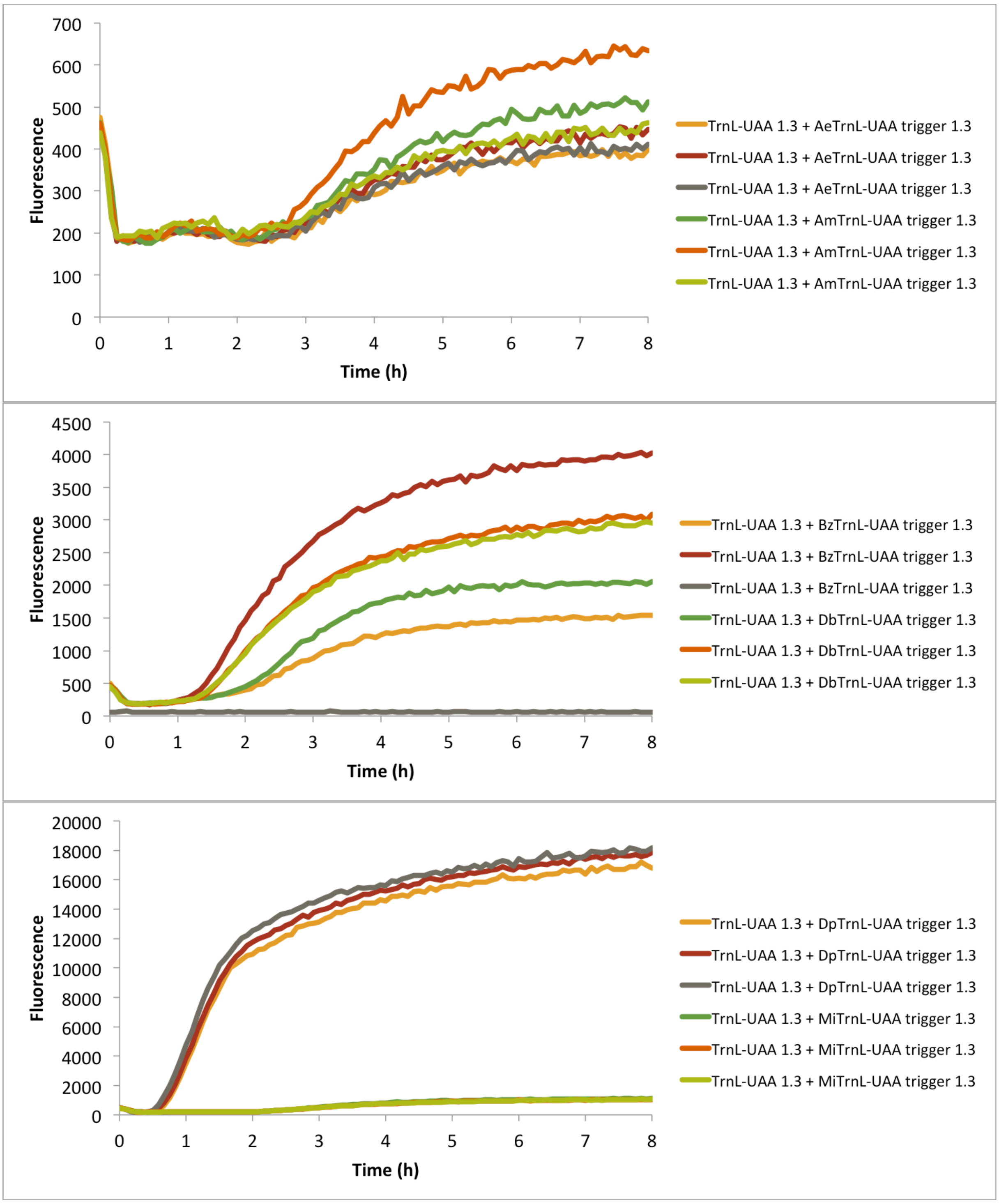

Supplementary Fig. S11. Comparison of the fold change ratio of the rosewood toehold switches against various triggers measured *in vivo* (Fig 6) versus their number of nucleotide mismatches compared to cognate triggers (A), free energy of the trigger-switch dimers secondary structures (B) and the equilibrium concentration of the trigger-switch dimers. Secondary-structure predictions and equilibrium concentrations evaluations were realized using the NUPACK web server [10] with default parameters. The initial concentration of both trigger and sensor RNA were set to 2 µM.

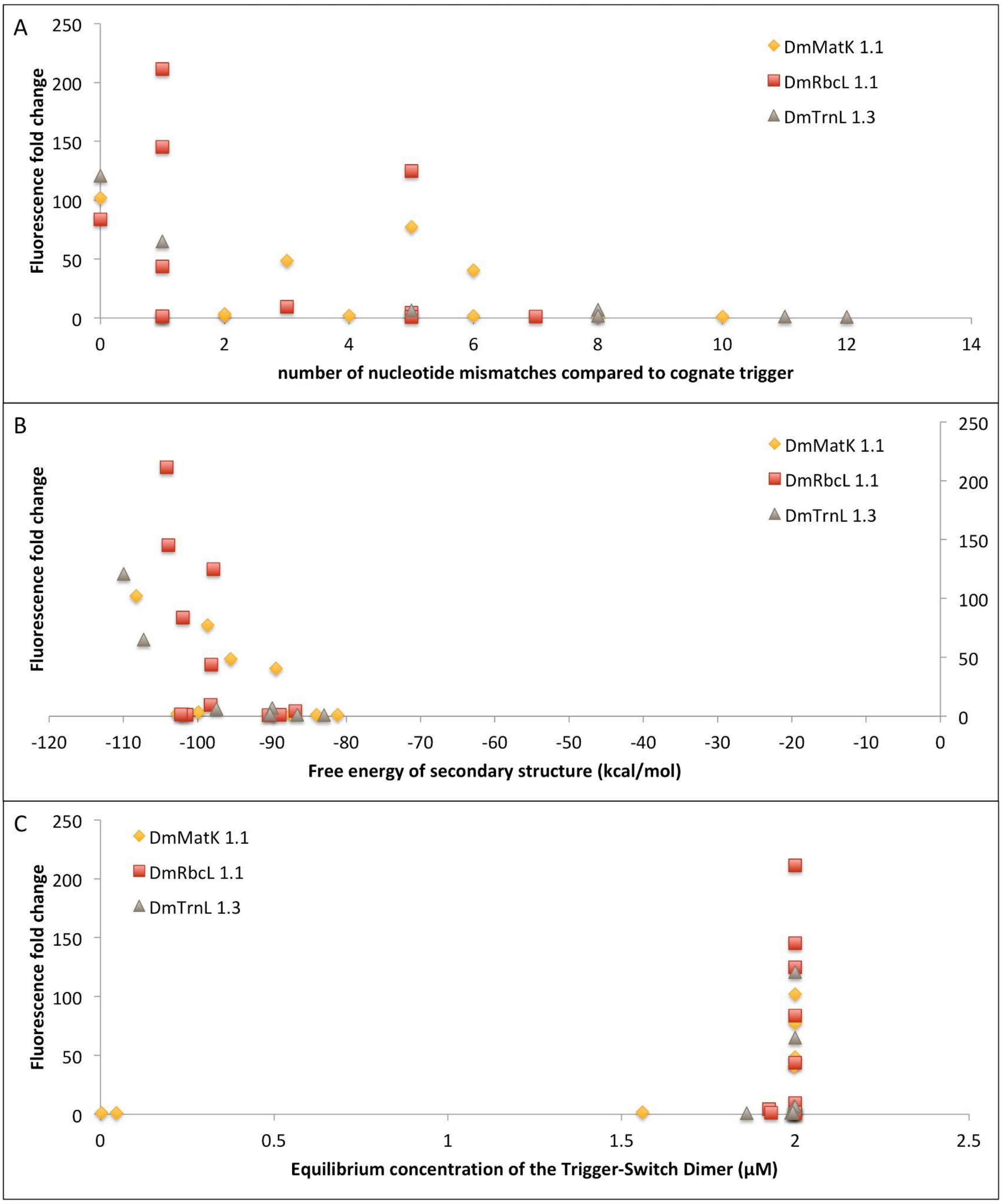

Supplementary Fig. S12. Secondary-structure predictions of various sensors-triggers pairs. The sensor switch sequences include the AUG of the reporter gene. The predictions were realized using the NUPACK web server [10] with default parameters and graphically represented using the forna RNA secondary structure visualization tool [11]. Nucleotides were coloured to match the different segments in Fig. 1. The SBa_000587 synthetic terminator is represented in blue.

Dalbergia maritima DmMatK Toehold Switch 1.1 Sensor
+ *Dalbergia maritima* DmMatK Trigger 1.1

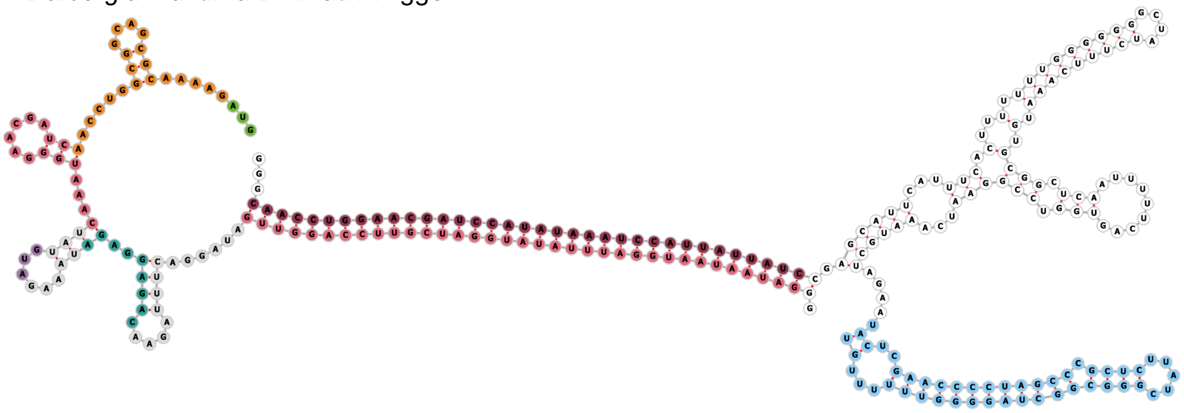

Dalbergia maritima DmMatK Toehold Switch 1.1 Sensor
+ *Acacia melanoxylon* AmMatK Trigger 1.1

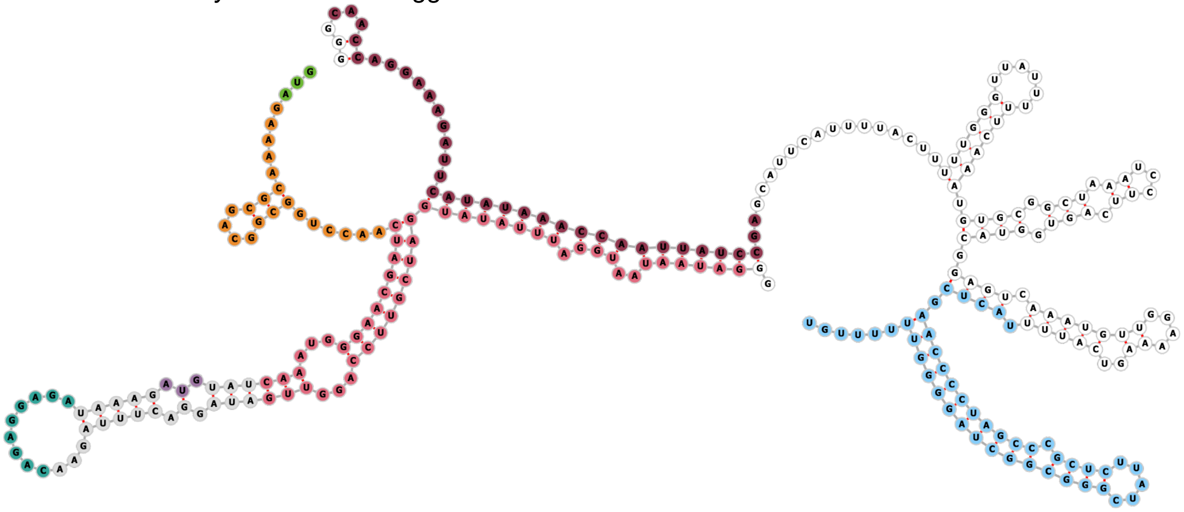

Dalbergia maritima DmMatK Toehold Switch 1.1 Sensor
+ *Berchemia zeyheri* BzMatK Trigger 1.1

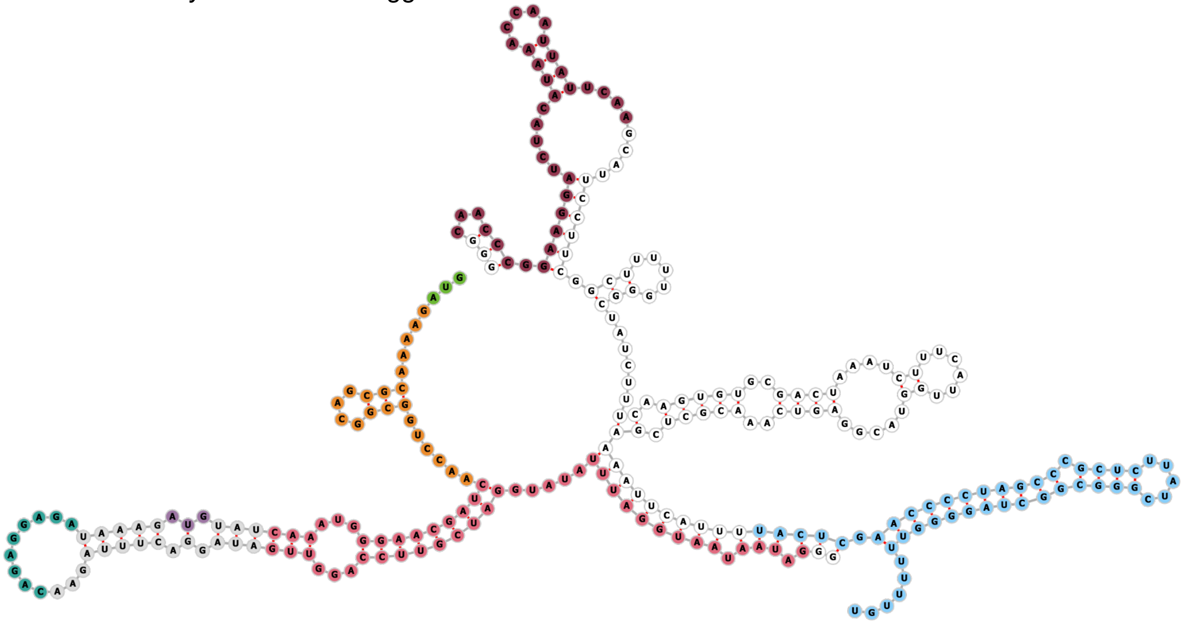

Dalbergia maritima DmMatK Toehold Switch 1.1 Sensor
+ *Dalbergia cochinchinensis* DcMatK Trigger 1.1

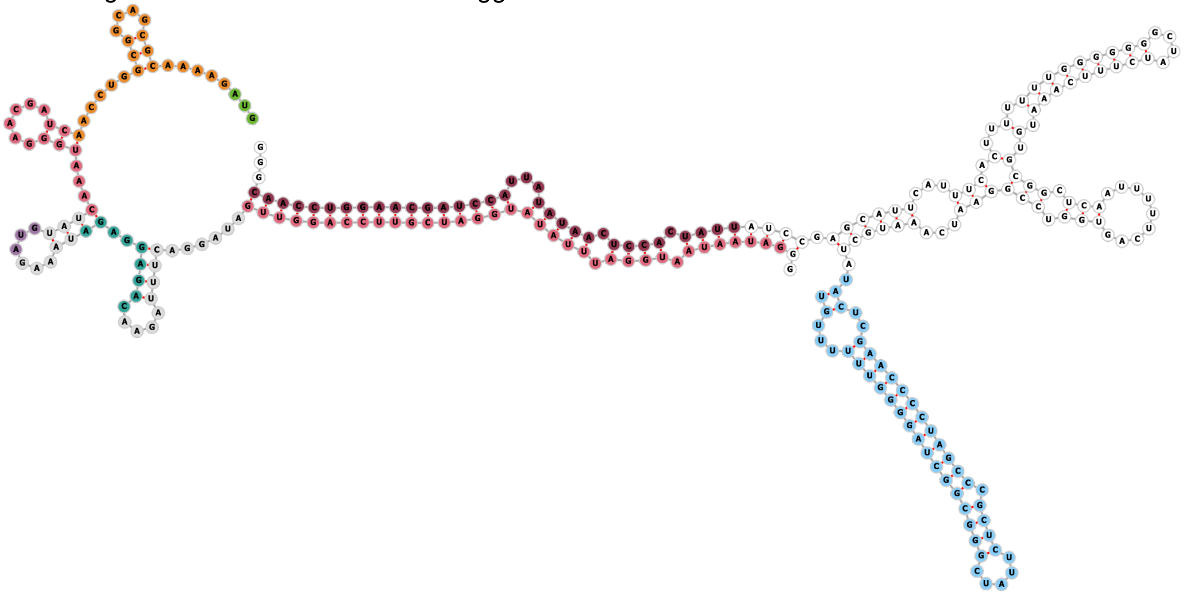

Dalbergia maritima DmMatK Toehold Switch 1.1 Sensor
\+ *Dalbergia hupeana* DhMatK Trigger 1.1

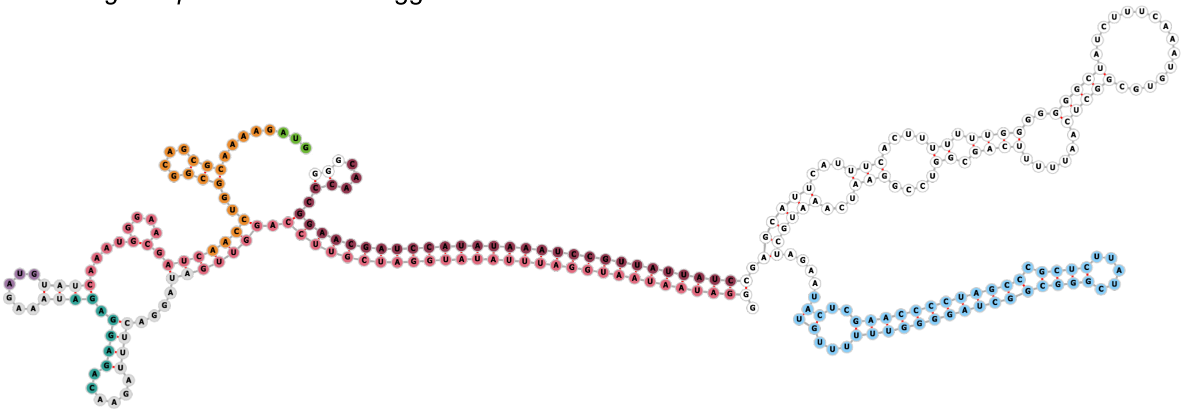

Dalbergia maritima DmMatK Toehold Switch 1.1 Sensor
\+ *Dalbergia ovata* DoMatK Trigger 1.1

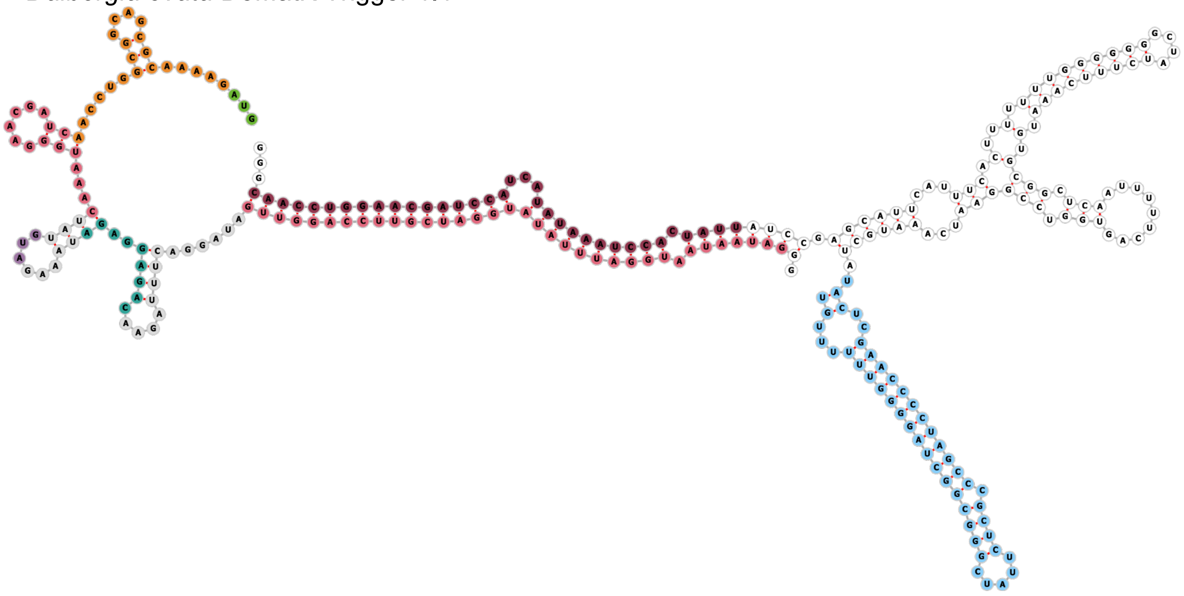

Dalbergia maritima DmMatK Toehold Switch 1.1 Sensor
\+ *Dalbergia pervillei* DpMatK Trigger 1.1

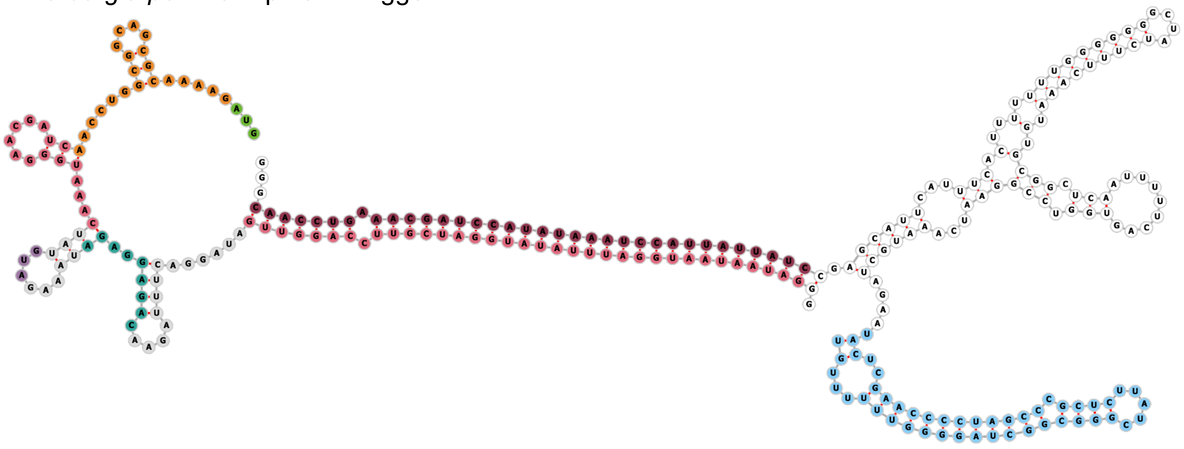

Dalbergia maritima DmMatK Toehold Switch 1.1 Sensor
+ *Erythroxylum havanense* EhMatK Trigger 1.1

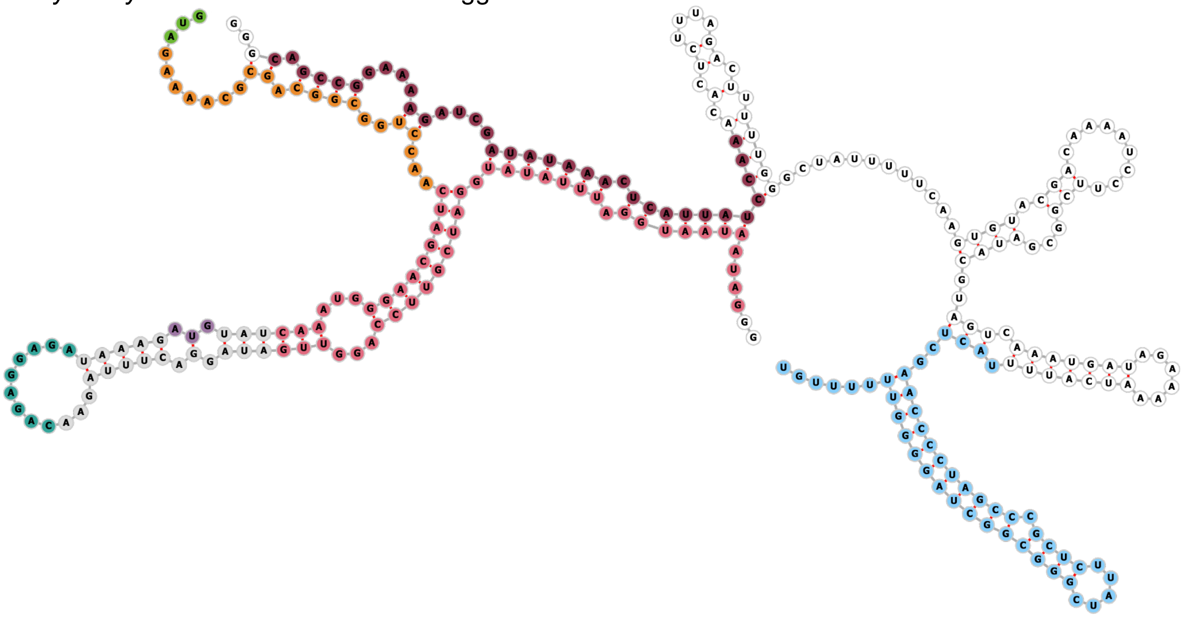

Dalbergia maritima DmMatK Toehold Switch 1.1 Sensor
+ *Millettia laurentii* MlMatK Trigger 1.1

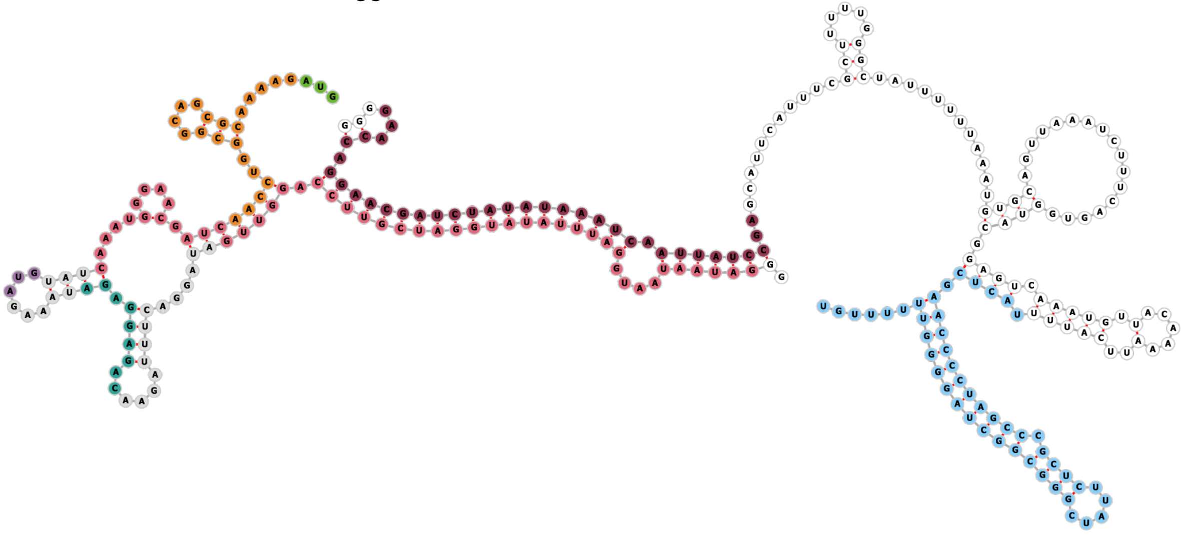

Dalbergia maritima DmMatK Toehold Switch 1.1 Sensor
+ *Machaerium scleroxylon* MsMatK Trigger 1.1

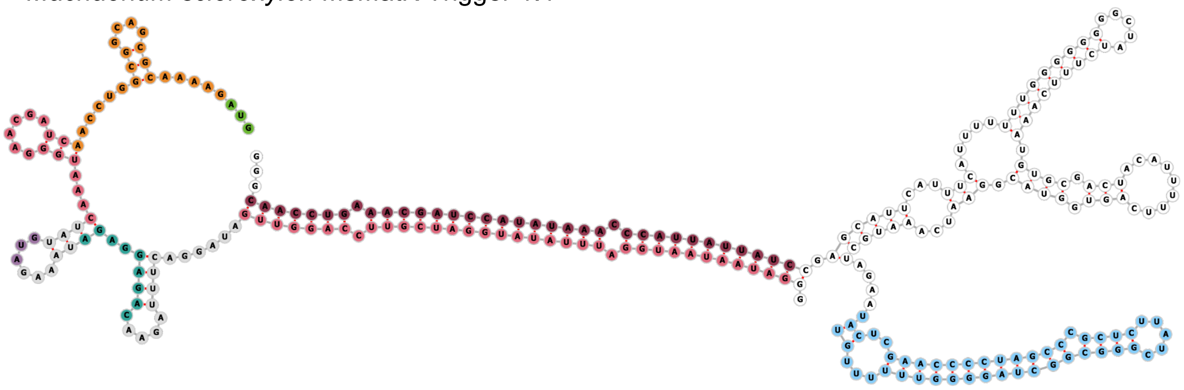

Dalbergia maritima DmMatK Toehold Switch 1.1 Sensor
+ *Pterocarpus indicus* PiMatK Trigger 1.1

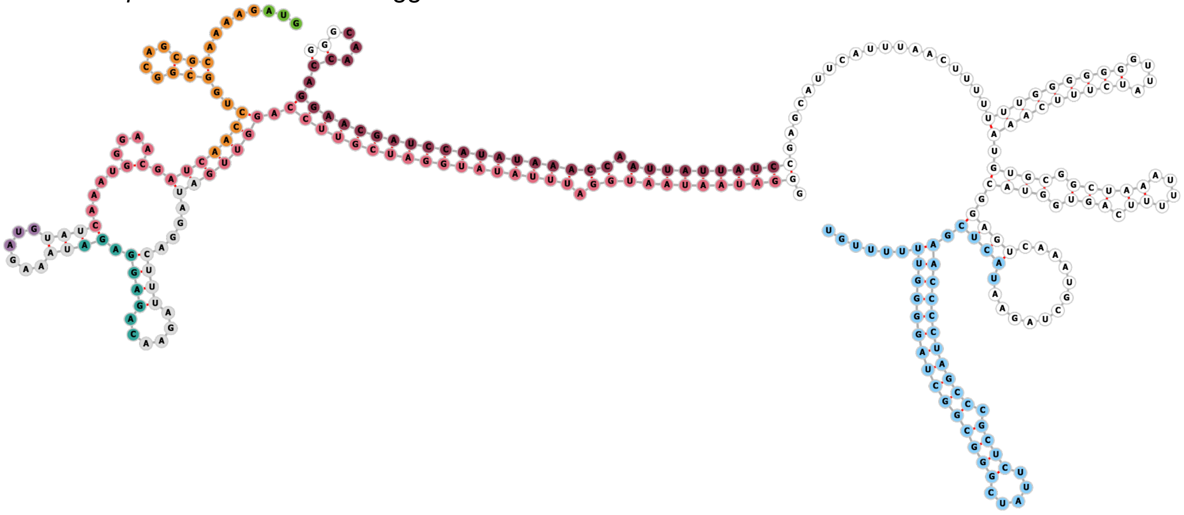

Dalbergia maritima DmRbcL Toehold Switch 1.1 Sensor
+ *Dalbergia maritima* DmRbcL Trigger 1.1

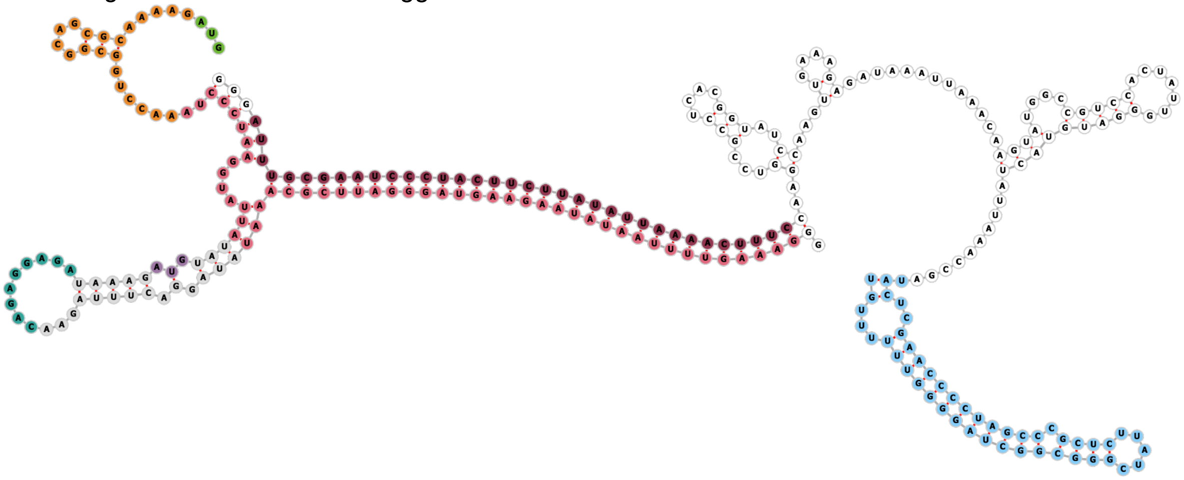

Dalbergia maritima DmRbcL Toehold Switch 1.1 Sensor
+ *Acacia melanoxylon* AmRbcL Trigger 1.1

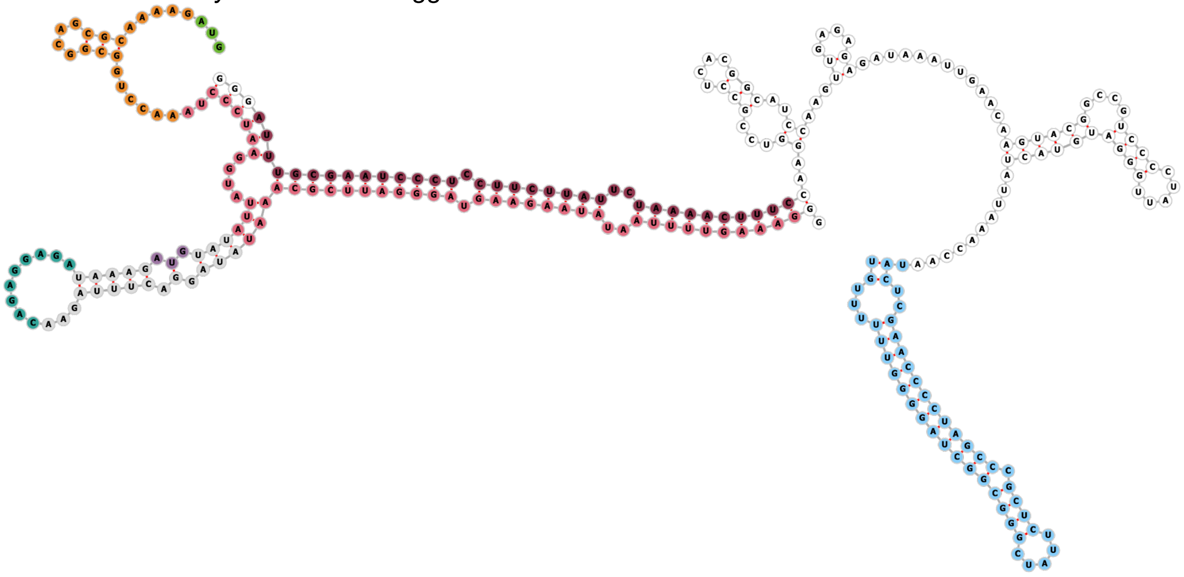

Dalbergia maritima DmRbcL Toehold Switch 1.1 Sensor
+ *Berchemia zeyheri* BzRbcL Trigger 1.1

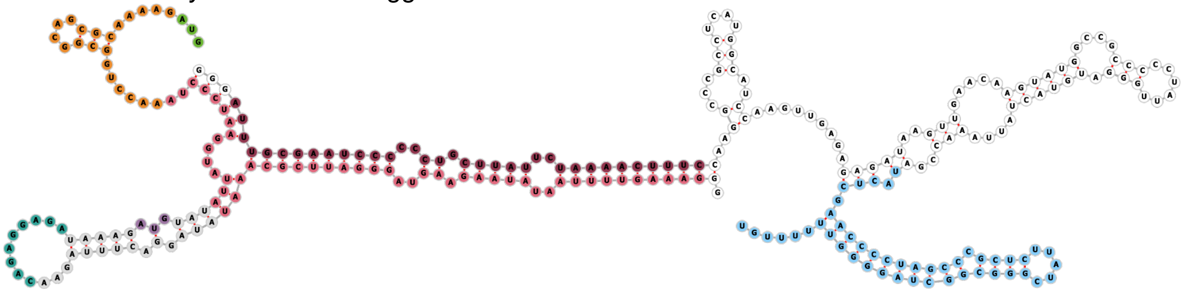

Dalbergia maritima DmRbcL Toehold Switch 1.1 Sensor
+ *Dalbergia baronii* DbRbcL Trigger 1.1

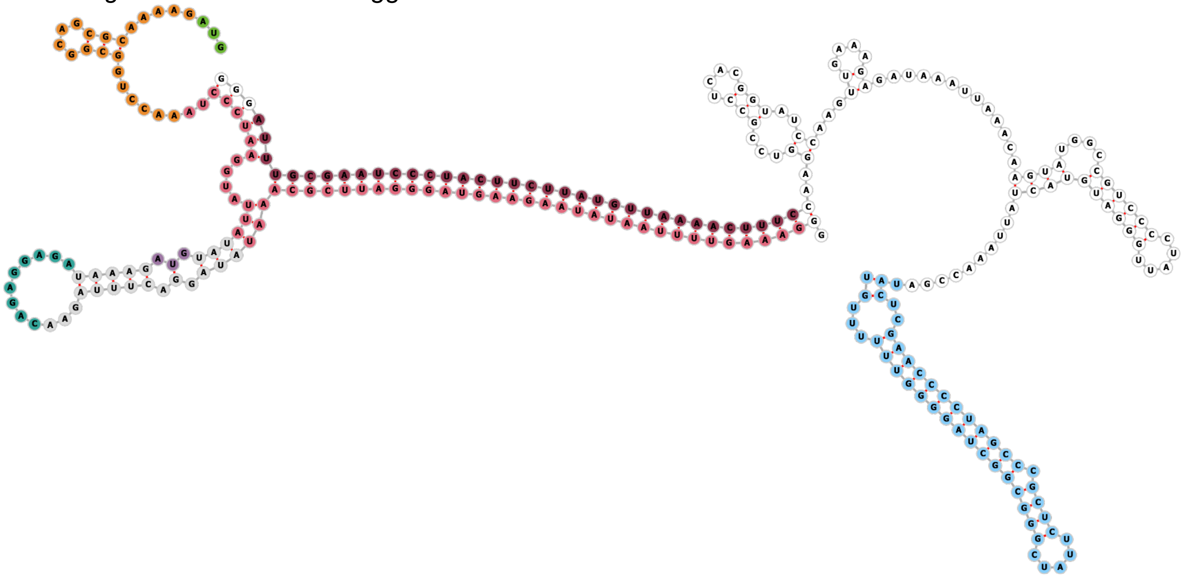

Dalbergia maritima DmRbcL Toehold Switch 1.1 Sensor
+ *Dysoxylum fraserianum* DfRbcL Trigger 1.1

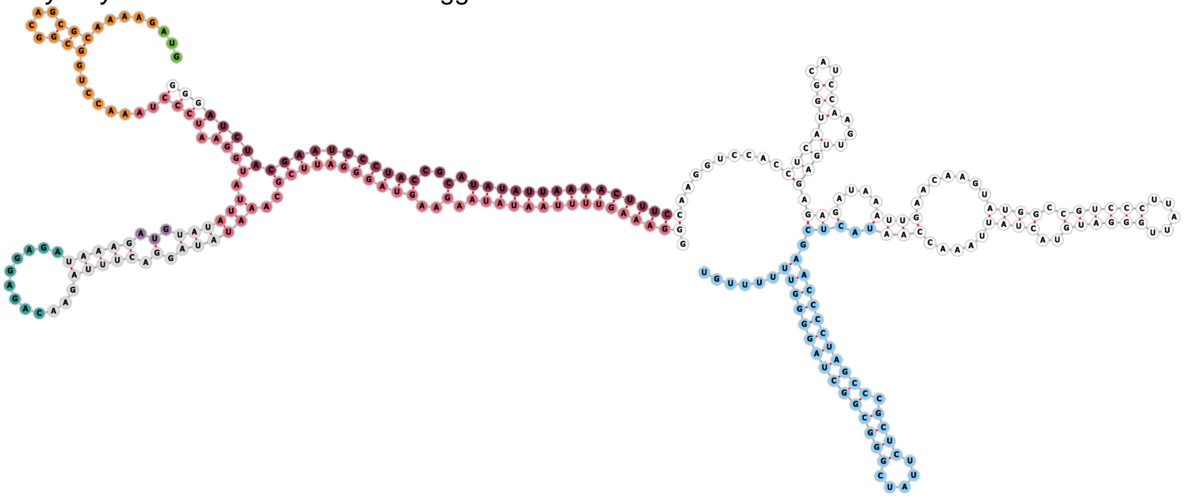

Dalbergia maritima DmRbcL Toehold Switch 1.1 Sensor
\+ *Dalbergia granadillo* DgRbcL Trigger 1.1

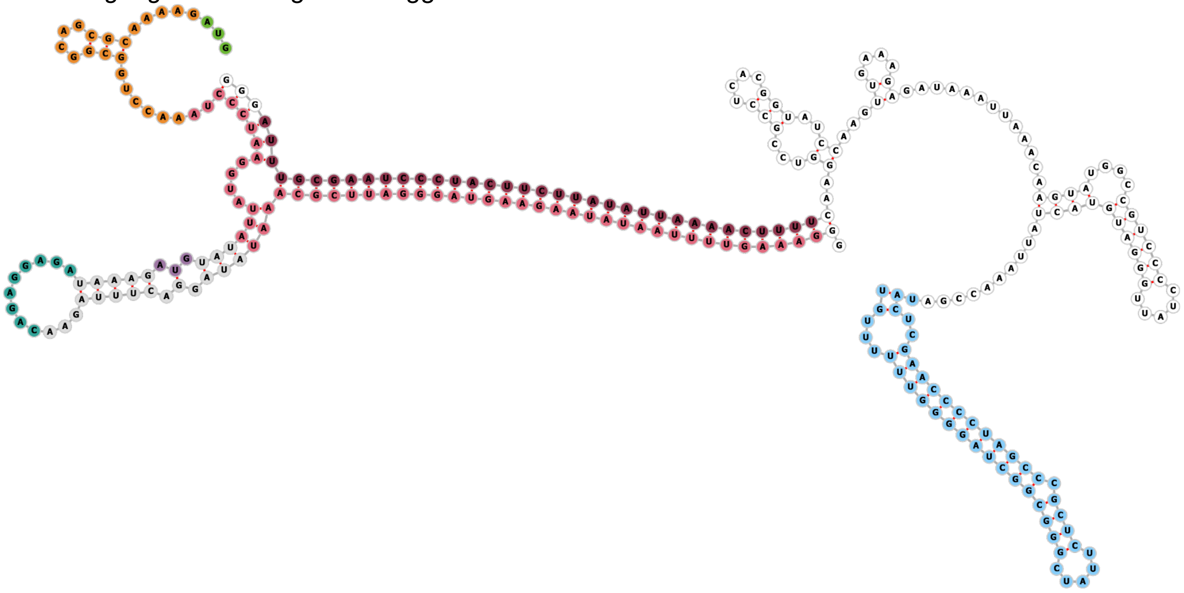

Dalbergia maritima DmRbcL Toehold Switch 1.1 Sensor
\+ *Dalbergia pervillei* DpRbcL Trigger 1.1

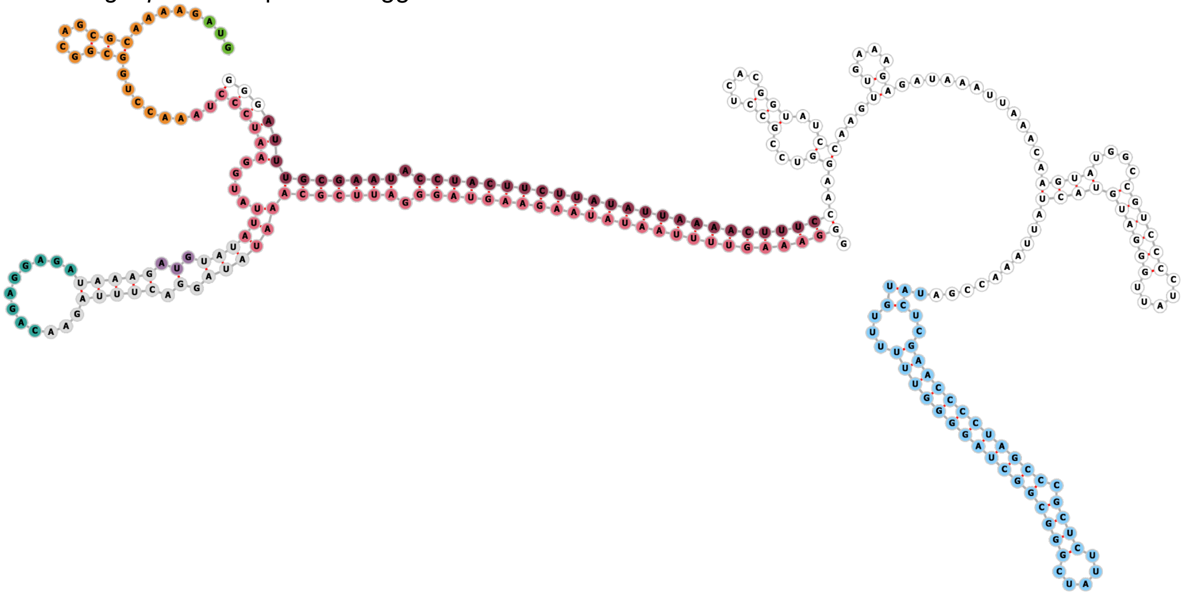

Dalbergia maritima DmRbcL Toehold Switch 1.1 Sensor
+ *Erythroxylum havanense* EhRbcL Trigger 1.1

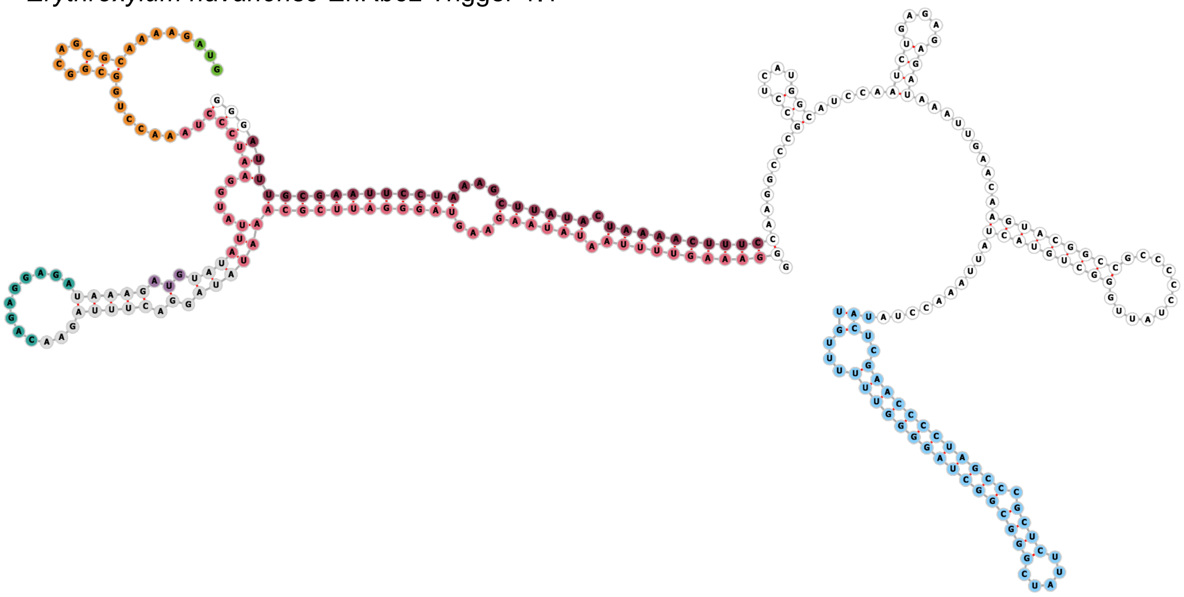

Dalbergia maritima DmRbcL Toehold Switch 1.1 Sensor
+ *Metopium brownei* MbRbcL Trigger 1.1

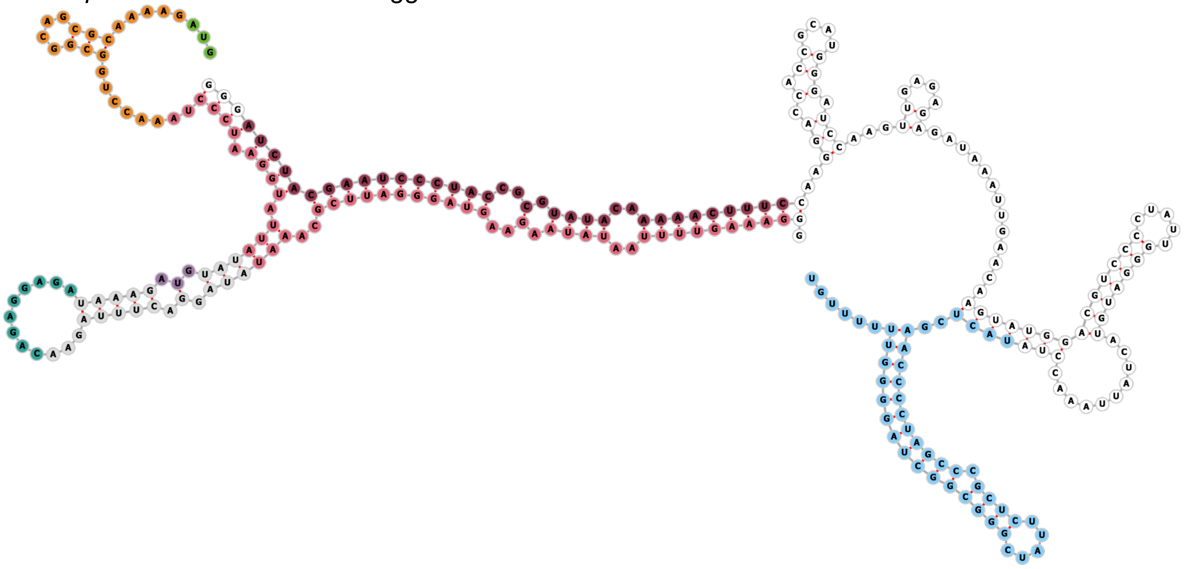

Dalbergia maritima DmRbcL Toehold Switch 1.1 Sensor
+ *Millettia laurentii* MlRbcL Trigger 1.1

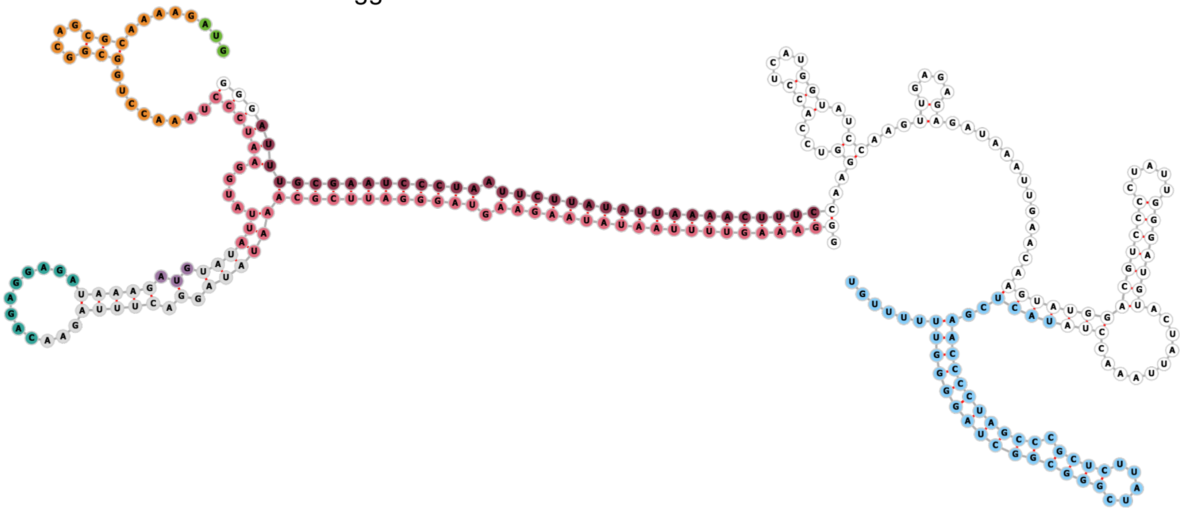

Dalbergia maritima DmRbcL Toehold Switch 1.1 Sensor
+ *Machaerium scleroxylon* MsRbcL Trigger 1.1

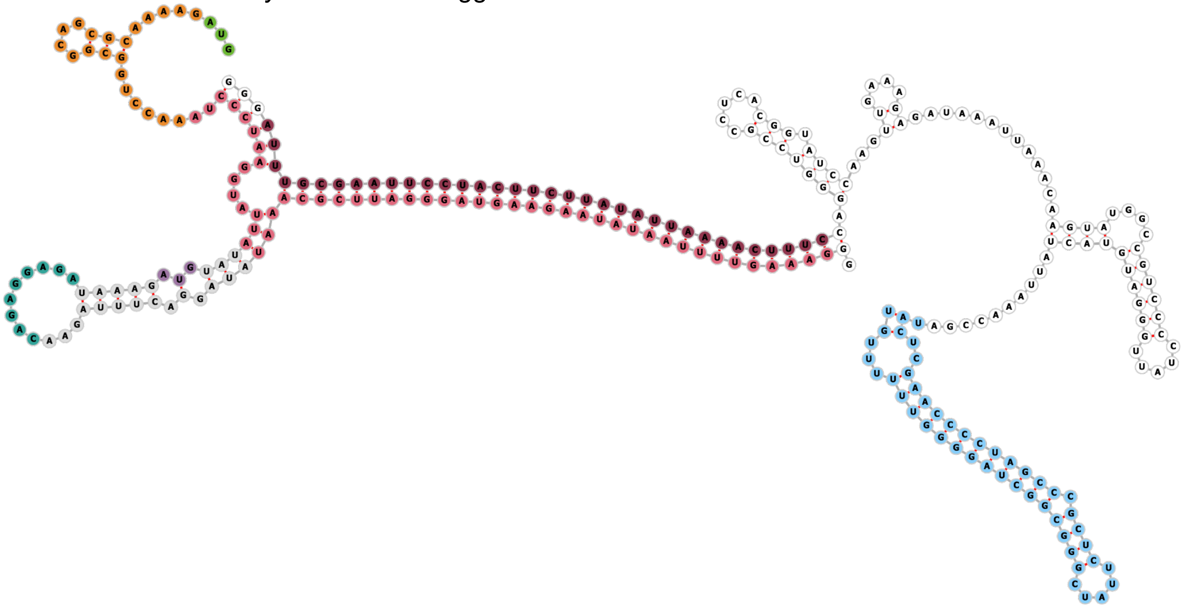

Dalbergia maritima DmTrnL-UAA Toehold Switch 1.3 Sensor
+ *Dalbergia maritima* DmTrnL-UAA RbcL Trigger 1.3

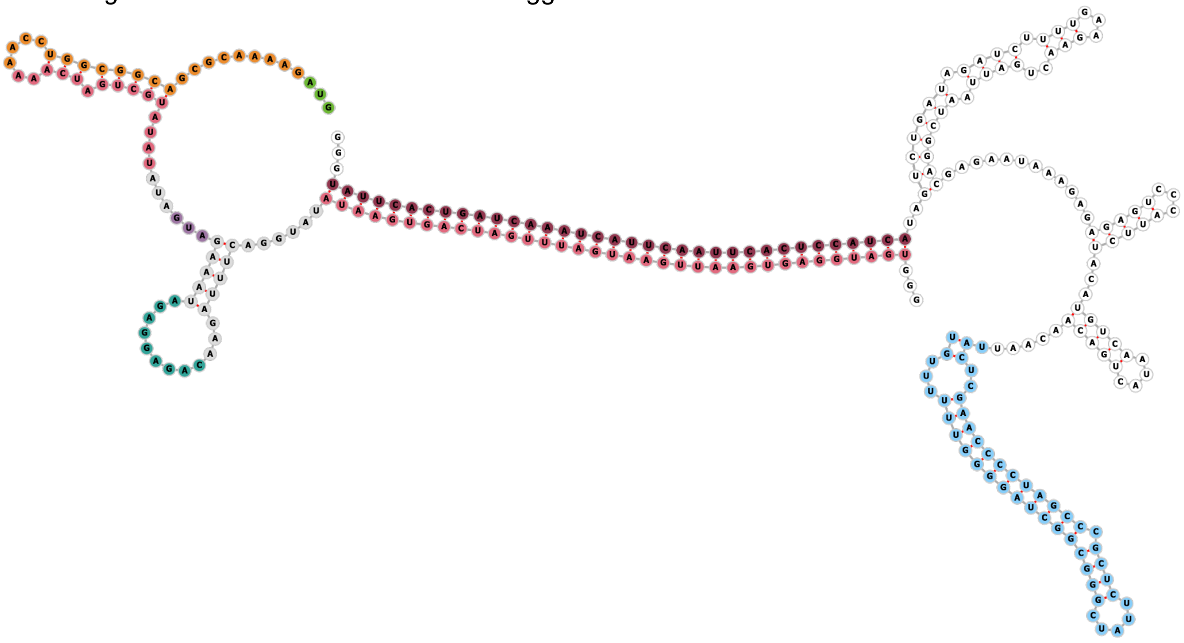

Dalbergia maritima DmTrnL-UAA Toehold Switch 1.3 Sensor
+ *Acacia excelsa* AeTrnL-UAA Trigger 1.3

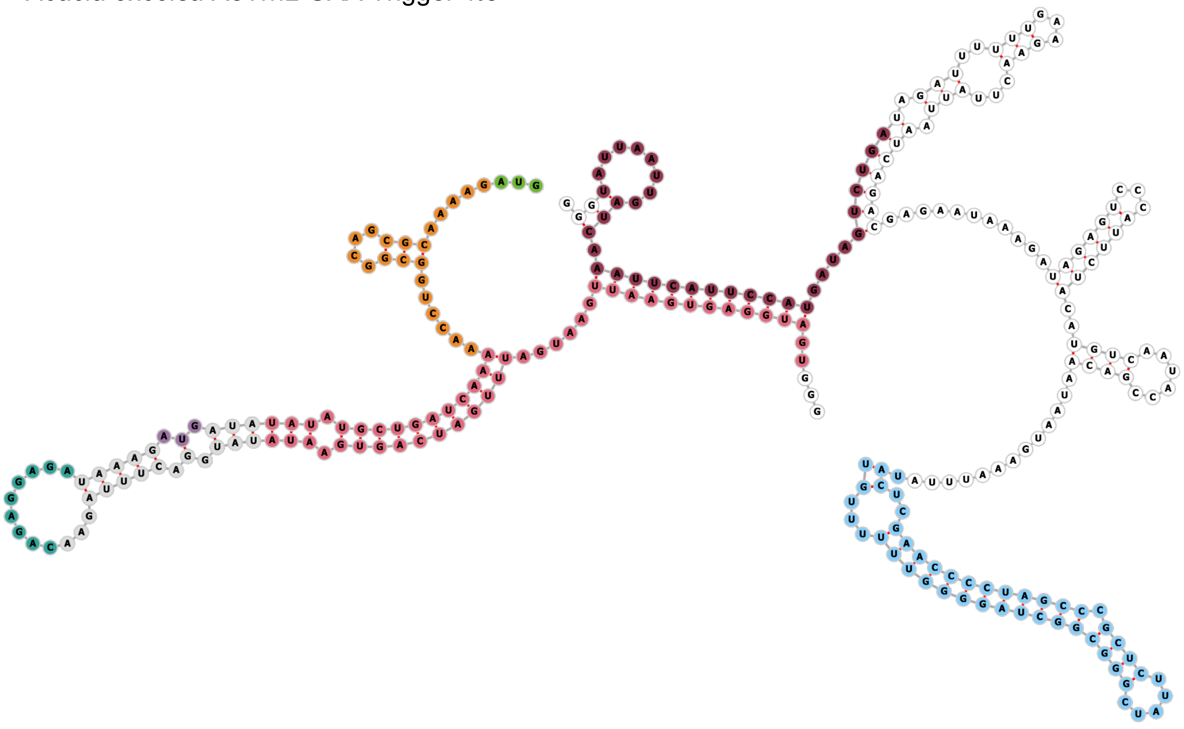

Dalbergia maritima DmTrnL-UAA Toehold Switch 1.3 Sensor
+ *Acacia melanoxylon* AmTrnL-UAA Trigger 1.3

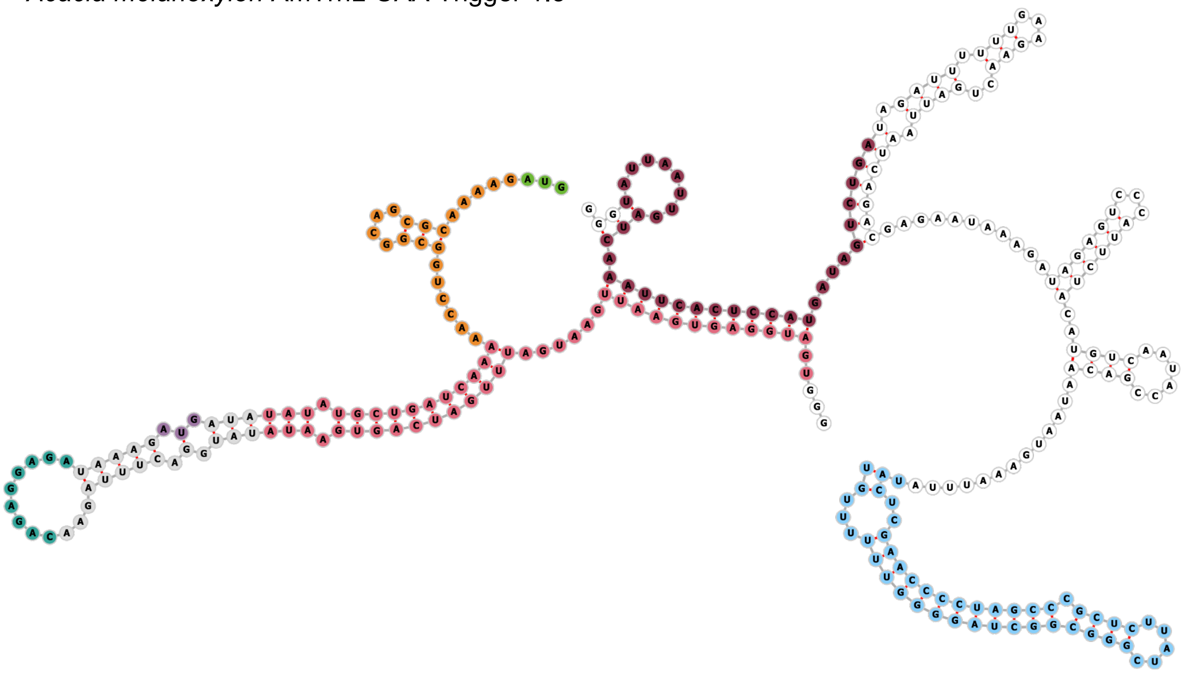

Dalbergia maritima DmTrnL-UAA Toehold Switch 1.3 Sensor
+ *Berchemia zeyheri* BzTrnL-UAA Trigger 1.3

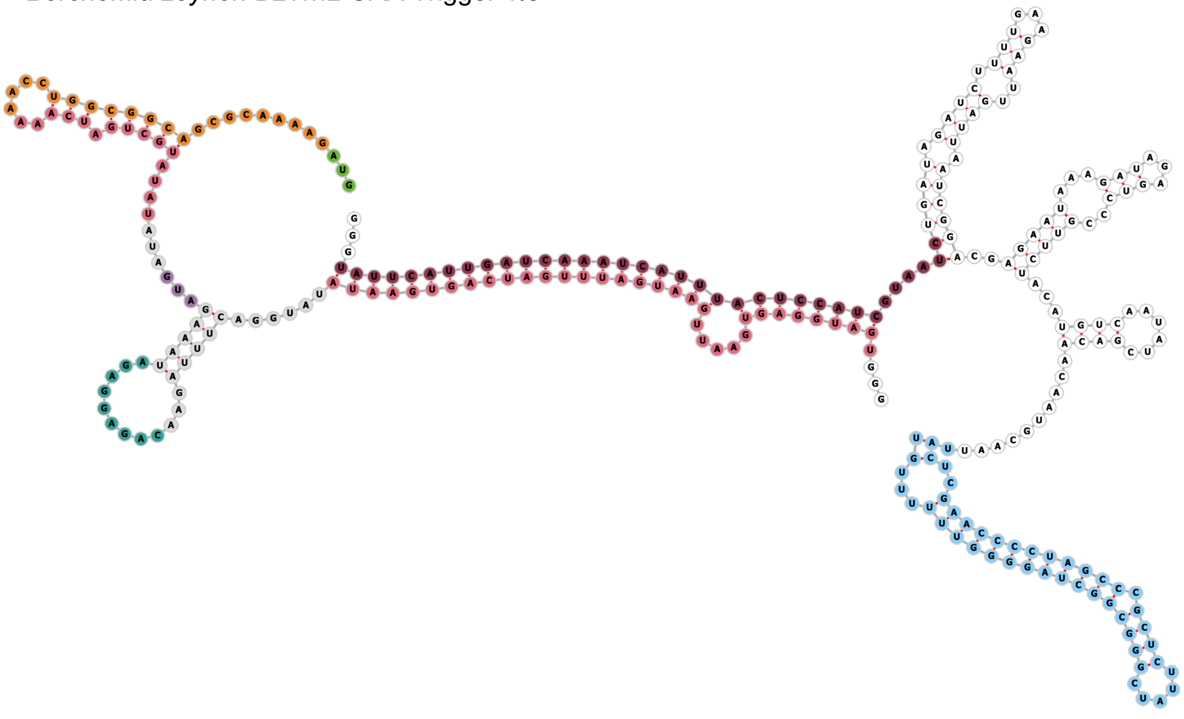

Dalbergia maritima DmTrnL-UAA Toehold Switch 1.3 Sensor
+ *Dalbergia baronii* DbTrnL-UAA Trigger 1.3

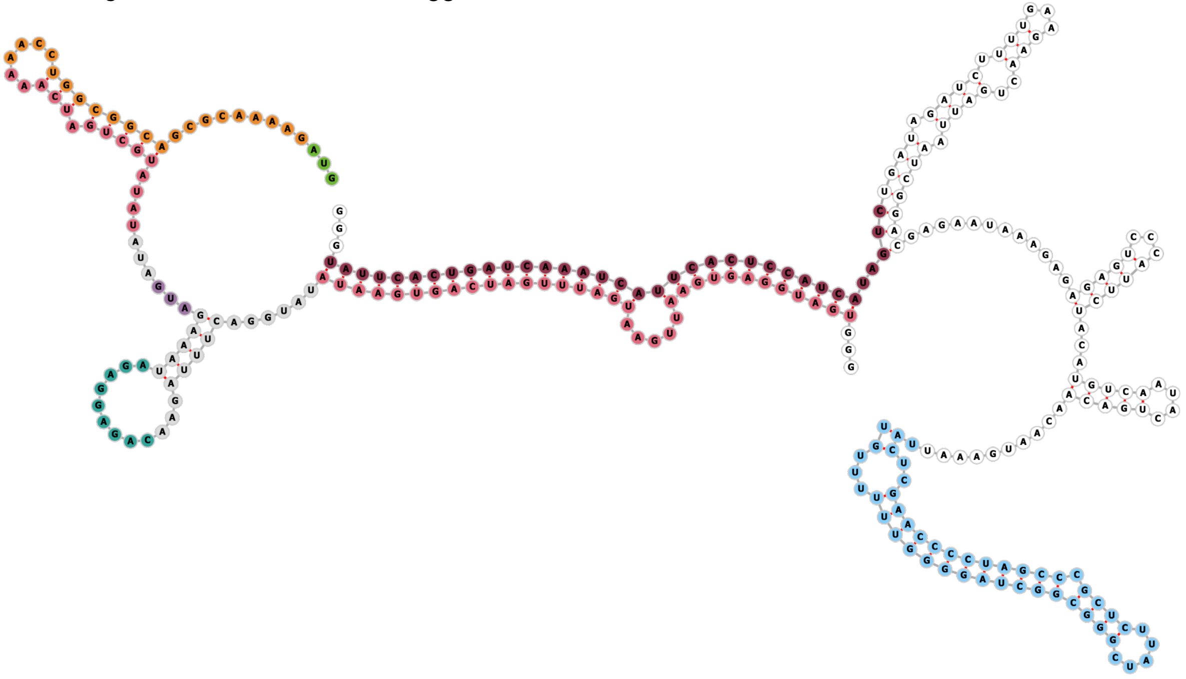

Dalbergia maritima DmTrnL-UAA Toehold Switch 1.3 Sensor
+ *Dalbergia pervillei* DpTrnL-UAA Trigger 1.3

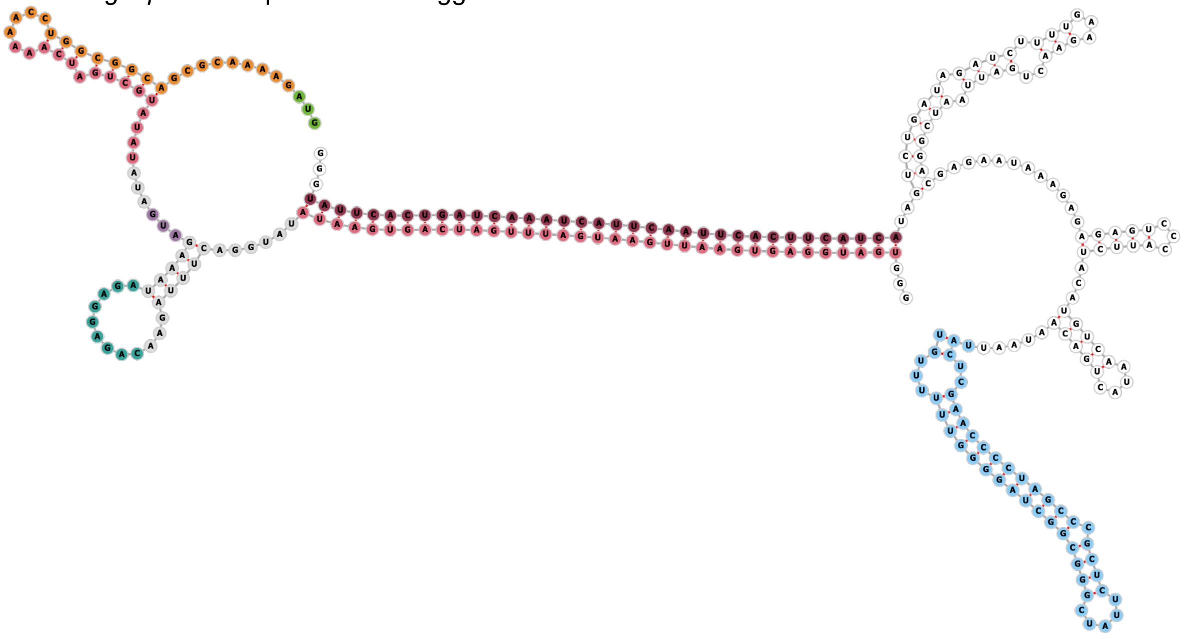

Dalbergia maritima DmTrnL-UAA Toehold Switch 1.3 Sensor
+ *Millettia laurentii* MlTrnL-UAA Trigger 1.3

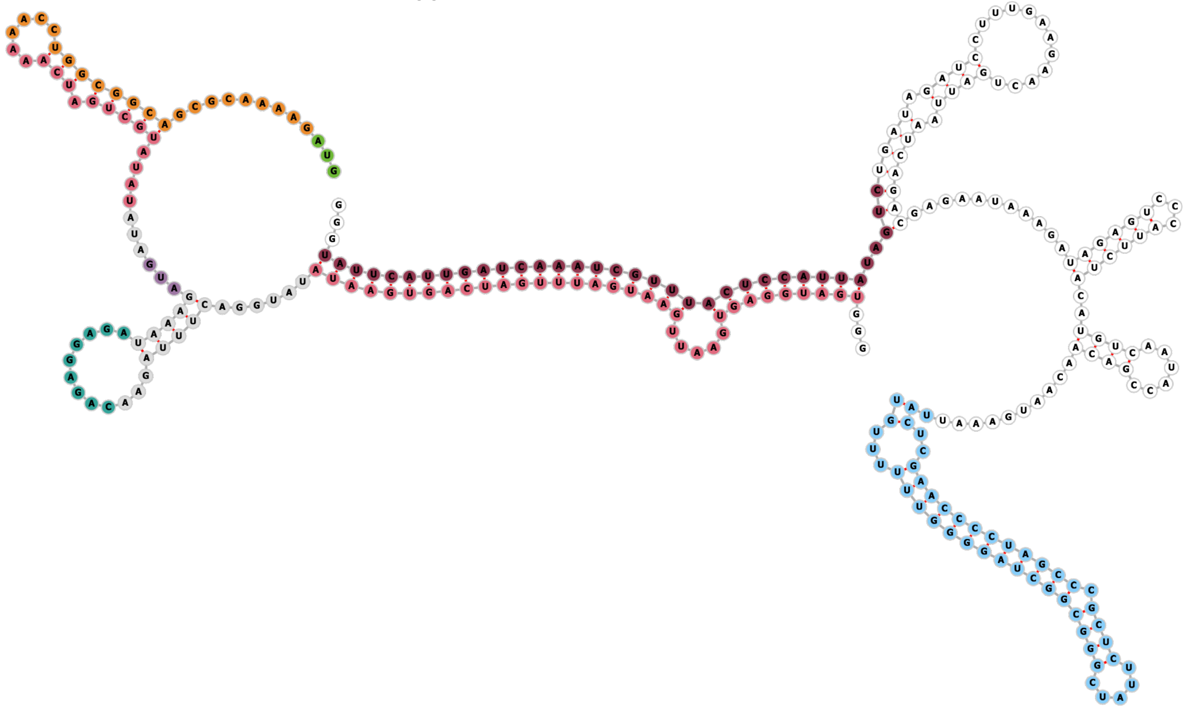

Supplementary Table S7. Sequences used in this study.

All plasmids were assembled following the Golden Gate cloning method [12,13] using either the NEB® Golden Gate Assembly Kit (BsaI-HF®v2) or the individual enzymes T4 DNA ligase, BsaI-HF®v2, BbsI-HF® from New England Biolabs and BsmBI from Thermo Fisher Scientific. All inserts were synthesized as double-stranded DNA fragments by Integrated DNA Technologies, Inc. (IDT). Q5® High-Fidelity DNA Polymerase (New England Biolabs) was used to carry out PCR reactions according to the supplier's protocol. Transformed *E. coli* strain DH5α cells were selected on LB medium containing either 35 µg/mL chloramphenicol (for pSB1C3 based plasmids) or 10 µg/mL tetracycline (for pSB3T5 based plasmids). Successful cloning was confirmed by sequencing at Eurofins Genomics. All nucleotide sequences were submitted to the iGEM's Registry of Standard Biological Parts (http://parts.igem.org/).

Sequence name	*Dalbergia maritima* DmMatK Toehold Switch 1.1 Sensor
Acc. number	BBa_K3453111
Sequence	tgccacctgacgtctaagaaaaggaatattcagcaatttgcccgtgccgaagaaaggcccacccgtgaaggtgagcc<br>agtgagttgattgctacgtaattagttagttagcccttagtgactggaattcgcggccgcttctagagtaatacgactcactat<br>agggataataatggatttatatggatcgttccaggttgataggactttagaacagaggagataaagatgtatcaaatggg<br>aacgatcaacctggcggcagcgcaaaagatgcgtaaaggcgaagaactgtttaccggtgtggttccgattctggtgga<br>actggacggcgatgttaatggtcataaattcagtgttcgcggcgaaggtgaaggcgatgcgacgaacggcaaactgac<br>cctgaaatttatctgcaccacgggtaaactgccggtcccgtggccgacgctggtgaccacgctgacctatggcgttcaat<br>gttttgcgcgttacccggatcacatgaaacagcacgactttttcaaatcggccatgccggaaggctatgtgcaggaacgt<br>acgattagctttaaagacgatggtacgtataaaacccgcgcggaagtgaaattcgaaggcgataccctggttaaccgta<br>tcgaactgaaaggtatcgatttcaaagaagatggcaatattctgggtcataaactggaatataacttcaattcccacaacg<br>tgtacatcaccgcggataaacagaaaaacggcattaaagccaatttcaaaatccgccataatgtggaagatggtagcg<br>ttcagctggccgaccactatcagcaaaacacgccgattggtgatggcccggtcctgctgccggacaatcactacctgag<br>tacccagtccgtgctgtcaaaagatccgaacgaaaaacgtgaccacatggtcctgctggaatttgtgacggctgcgggt<br>atcacccacggcatggacgaactgtataaaggcctgctgcaaacgacgaaaactacgctttagtagcttaataatact<br>cgaacccctagcccgctcttatcgggcggctaggggtttttttgttactagtagcggccgctgcaggagtcactaagggtta<br>gttagttagattagcagaaagtcaaaagcctccgaccggaggcttttgactaaaacttcccttggggttatcattggggctc<br>actcaaaggcggtaatcagataaaaaaaatccttagctttcgctaaggatgatttctgctagagatggaatagactggat<br>ggaggcggataaagttgcaggaccacttctgcgctcggcccttccggctggctggtttattgctgataaatctggagccgg<br>tgagcgtgggactcgcggtatcattgcagcactggggccagatggtaagccctcccgtatcgtagttatctacacgacgg<br>ggagtcaggcaactatggatgaacgaaatagacagatcgctgagataggtgcctcactgattaagcattggtaactgtc<br>agaccaagtttactcatatatactttagattgatttaaaacttcatttttaatttaaaaggatctaggtgaagatcctttttgataat<br>ctcatgaccaaaatcccttaacgtgagttttcgttccactgagcgtcagaccccttaataagatgatcttcttgagatcgttttg<br>gtctgcgcgtaatctcttgctctgaaaacgaaaaaaccgccttgcagggcggtttttcgaaggttctctgagctaccaactc<br>tttgaaccgaggtaactggcttggaggagcgcagtcaccaaaacttgtcctttcagtttagccttaaccggcgcatgacttc<br>aagactaactcctctaaatcaattaccagtggctgctgccagtggtgcttttgcatgtctttccgggttggactcaagacgat<br>agttaccggataaggcgcagcggtcggactgaacggggggttcgtgcatacagtccagcttggagcgaactgcctacc<br>cggaactgagtgtcaggcgtggaatgagacaaacgcggccataacagcggaatgacaccggtaaaccgaaaggc<br>aggaacaggagagcgcacgagggagccgccaggggaaacgcctggtatctttatagtcctgtcgggtttcgccacca<br>ctgatttgagcgtcagatttcgtgatgcttgtcaggggggcggagcctatggaaaaacggctttgccgcggccctctcactt<br>ccctgttaagtatcttcctggcatcttccaggaaatctccgccccgttcgtaagccatttccgctcgccgcagtcgaacgac<br>cgagcgtagcgagtcagtgagcgaggaagcggaatatatcctgtatcacatattctgctgacgcaccggtgcagcctttt<br>ttctcctgccacatgaagcacttcactgacaccctcatcagtgccaacatagtaagccagtatacactccgctagcgctg<br>aggtctgcctcgtgaagaaggtgttgctgactcataccaggcctgaatcgccccatcatccagccagaaagtgaggga<br>gccacggttgatgagagctttgttgtaggtggaccagttggtgattttgaacttttgctttgccacggaacggtctgcgttgtcg<br>ggaagatgcgtgatctgatccttcaactcagcaaaagttcgatttattcaacaaagccacgttgtgtctcaaaatctctgat<br>gttacattgcacaagataaaaatatatcatcatgaacaataaaactgtctgcttacataaacagtaatacaaggggtgttt<br>actagaggagattctcatgtttgacagcttatcatcgataagctttaatgcggtagtttatcacagttaaattgctaacgcagt<br>caggcaccgtgtatgaaatctaacaatgcgctcatcgtcattctcggcaccgtcaccctggacgctgtaggcataggctt<br>ggttatgccggtactgccgggcctcttgcgggatatcgtccattccgacagtattgccagtcactatggcgtgctgcttgcg<br>ctctatgcgttgatgcaatttctttgcgcacccgttctcggagccctgtccgaccgctttggccgccgtccagtcctgctcgctt<br>cgctccttggagccactatcgactacgcgatcatggcgaccacacccgtcctgtggattctctacgccggacgcatcgtg<br>gcgggcatcacgggtgccacaggtgcggttgctggtgcctatatcgccgacatcaccgatggggaagatcgggctcgc<br>cacttcgggctcatgagcgcttgtttcggcgtgggtatggtggcaggccccgtggccgggggactgttgggtgccatctcc<br>ttgcatgcaccattccttgcggcggcggtgctcaacggcctcaacctcctcctgggctgcttccttatgcaggaatcgcata<br>agggagagcgccgtccgatgcccttgcgtgccttcaatccagtcagctccttccggtgggcgcggggcatgactatcgtc

	gccgcacttatgactgttttctttatcatgcaactcgtaggacaggttccggcagcgctctgggtcattttcggcgaggaccg<br>ctttcgctggagcgcgacgatgatcggcctgtcgcttgcggtattcggaatcttgcacgccctcgctcaagccttcgtcacg<br>ggccccgccaccaaacgtttcggcgagaagcaggccattatcgcgggcatggcggccgacgcgctgggctacgtctt<br>gctggcgttcgcgacgcgcggctggatggccttccccattatgattcttctcgcttccggcggcatcggtatgcccgcgttg<br>caggccatgctgtcccgccaagtagatgacgaccatcagggacagcttcaagggtcgctcgcggctcttaccagcctc<br>acttcgatcattggaccgctgatcgtcacggcgatttatgccgcctcggcgagcacatggaacgggttggcatggattgta<br>ggtgccgccctttaccttgtctgcctccccgcgttgcgtcgcggtgcatggagccgggccacctcgacctaataatactag<br>ctccggcaaaaaaacgggcaaggtgtcaccaccctgcccttttctttaaaaccgaaaagattacttcgcgtt

Sequence name	*Dalbergia maritima* DmMatK Toehold Switch 1.1 Trigger
Acc. number	BBa_K3453121
Sequence	tgccacctgacgtctaagaaaccattattatcatgacattaacctataaaaataggcgtatcacgaggcagaatttcagat<br>aaaaaaaatccttagctttcgctaaggatgatttctggaattcgcggccgcttctagagtaatacgactcactatagggca<br>acctggaacgatccatataaatccattattatccgagcattcatttcactttttttgggggggctatctttcaaatgtgcggctc<br>aatttttcagtggtccggaatcaaatgctagaatactcgaacccctagcccgctcttatcgggcggctaggggtttttttgttac<br>tagtagcggccgctgcagtccggcaaaaaagggcaaggtgtcaccaccctgcccttttctttaaaaccgaaaagatta<br>cttcgcgttatgcaggcttcctcgctcactgactcgctgcgctcggtcgttcggctgcggcgagcggtatcagctcactcaa<br>aggcggtaatacggttatccacagaatcaggggataacgcaggaaagaacatgtgagcaaaaggccagcaaaag<br>gccaggaaccgtaaaaaggccgcgttgctggcgtttttccacaggctccgcccccctgacgagcatcacaaaaatcga<br>cgctcaagtcagaggtggcgaaacccgacaggactataaagataccaggcgtttccccctggaagctccctcgtgcgc<br>tctcctgttccgaccctgccgcttaccggatacctgtccgcctttctcccttcgggaagcgtggcgctttctcatagctcacgc<br>tgtaggtatctcagttcggtgtaggtcgttcgctccaagctgggctgtgtgcacgaaccccccgttcagcccgaccgctgc<br>gccttatccggtaactatcgtcttgagtccaacccggtaagacacgacttatcgccactggcagcagccactggtaacag<br>gattagcagagcgaggtatgtaggcggtgctacagagttcttgaagtggtggcctaactacggctacactagaagaac<br>agtatttggtatctgcgctctgctgaagccagttaccttcggaaaaagagttggtagctcttgatccggcaaacaaaccac<br>cgctggtagcggtggtttttttgtttgcaagcagcagattacgcgcagaaaaaaaggatctcaagaagatcctttgatctttt<br>ctacggggtctgacgctcagtggaacgaaaactcacgttaagggattttggtcatgagattatcaaaaaggatcttcacct<br>agatccttttaaattaaaaatgaagttttaaatcaatctaaagtatatatgagtaaacttggtctgacagctcgaggcttggat<br>tctcaccaataaaaaacgcccggcggcaaccgagcgttctgaacaaatccagatggagttctgaggtcattactggatc<br>tatcaacaggagtccaagcgagctcgatatcaaattacgccccgccctgccactcatcgcagtactgttgtaattcattaa<br>gcattctgccgacatggaagccatcacaaacggcatgatgaacctgaatcgccagcggcatcagcaccttgtcgccttg<br>cgtataatatttgcccatggtgaaaacgggggcgaagaagttgtccatattggccacgtttaaatcaaaactggtgaaac<br>tcacccagggattggctgagacgaaaaacatattctcaataaaccctttagggaaataggccaggttttcaccgtaaca<br>cgccacatcttgcgaatatatgtgtagaaactgccggaaatcgtcgtggtattcactccagagcgatgaaaacgtttcagt<br>ttgctcatggaaaacggtgtaacaagggtgaacactatcccatatcaccagctcaccgtctttcattgccatacgaaattc<br>cggatgagcattcatcaggcgggcaagaatgtgaataaaggccggataaaacttgtgcttatttttctttacggtctttaaa<br>aaggccgtaatatccagctgaacggtctggttataggtacattgagcaactgactgaaatgcctcaaaatgttctttacgat<br>gccattgggatatatcaacggtggtatatccagtgatttttttctccattttagcttccttagctcctgaaaatctcgataactca<br>aaaaatacgcccggtagtgatcttatttcattatggtgaaagttggaacctcttacgtgcccgatcaactcgag

Sequence name	*Dalbergia maritima* DmMatK Toehold Switch 1.3 Sensor
Acc. number	BBa_K3453112
Sequence	tgccacctgacgtctaagaaaaggaatattcagcaatttgcccgtgccgaagaaaggcccacccgtgaaggtgagcc<br>agtgagttgattgctacgtaattagttagttagcccttagtgactggaattcgcggccgcttctagagtaatacgactcactat<br>agggtttattcatcaaaagaggcgcattctttgaagccatatggactttagaacagaggagataaagatgatatggatgc<br>aaagaatgaacctggcggcagcgcaaaagatgcgtaaaggcgaagaactgtttaccggtgtggttccgattctggtgg<br>aactggacggcgatgttaatggtcataaattcagtgttcgcggcgaaggtgaaggcgatgcgacgaacggcaaactg<br>accctgaaatttatctgcaccacgggtaaactgccggtcccgtggccgacgctggtgaccacgctgacctatggcgttca<br>atgttttgcgcgttacccggatcacatgaaacagcacgactttttcaaatcggccatgccggaaggctatgtgcaggaac<br>gtacgattagctttaaagacgatggtacgtataaaacccgcgcggaagtgaaattcgaaggcgataccctggttaaccg<br>tatcgaactgaaaggtatcgatttcaaagaagatggcaatattctgggtcataaactggaatataacttcaattcccacaa<br>cgtgtacatcaccgcggataaacagaaaaacggcattaaagccaatttcaaaatccgccataatgtggaagatggtag<br>cgttcagctggccgaccactatcagcaaaacacgccgattggtgatggcccggtcctgctgccggacaatcactacctg<br>agtacccagtccgtgctgtcaaaagatccgaacgaaaaacgtgaccacatggtcctgctggaatttgtgacggctgcgg<br>gtatcacccacggcatggacgaactgtataaaggcctgctgcaaacgacgaaaactacgctttagtagcttaataata<br>ctcgaacccctagcccgctcttatcgggcggctaggggtttttttgttactagtagcggccgctgcaggagtcactaagggtt<br>agttagttagattagcagaaagtcaaaagcctccgaccggaggcttttgactaaaacttcccttggggttatcattggggct<br>cactcaaaggcggtaatcagataaaaaaaatccttagctttcgctaaggatgatttctgctagagatggaatagactgga

	tggaggcggataaagttgcaggaccacttctgcgctcggcccttccggctggctggtttattgctgataaatctggagccg<br>gtgagcgtgggactcgcggtatcattgcagcactggggccagatggtaagccctcccgtatcgtagttatctacacgacg<br>gggagtcaggcaactatggatgaacgaaatagacagatcgctgagataggtgcctcactgattaagcattggtaactgt<br>cagaccaagtttactcatatatactttagattgatttaaaacttcatttttaatttaaaaggatctaggtgaagatcctttttgata<br>atctcatgaccaaaatcccttaacgtgagttttcgttccactgagcgtcagaccccttaataagatgatcttcttgagatcgttt<br>tggtctgcgcgtaatctcttgctctgaaaacgaaaaaaccgccttgcagggcggtttttcgaaggttctctgagctaccaac<br>tctttgaaccgaggtaactggcttggaggagcgcagtcaccaaaacttgtcctttcagtttagccttaaccggcgcatgact<br>tcaagactaactcctctaaatcaattaccagtggctgctgccagtggtgcttttgcatgtctttccgggttggactcaagacg<br>atagttaccggataaggcgcagcggtcggactgaacggggggttcgtgcatacagtccagcttggagcgaactgccta<br>cccggaactgagtgtcaggcgtggaatgagacaaacgcggccataacagcggaatgacaccggtaaaccgaaag<br>gcaggaacaggagagcgcacgagggagccgccaggggaaacgcctggtatctttatagtcctgtcgggtttcgccac<br>cactgatttgagcgtcagatttcgtgatgcttgtcaggggggcggagcctatggaaaaacggctttgccgcggccctctca<br>cttccctgttaagtatcttcctggcatcttccaggaaatctccgccccgttcgtaagccatttccgctcgccgcagtcgaacg<br>accgagcgtagcgagtcagtgagcgaggaagcggaatatatcctgtatcacatattctgctgacgcaccggtgcagcct<br>tttttctcctgccacatgaagcacttcactgacaccctcatcagtgccaacatagtaagccagtatacactccgctagcgct<br>gaggtctgcctcgtgaagaaggtgttgctgactcataccaggcctgaatcgccccatcatccagccagaaagtgaggg<br>agccacggttgatgagagctttgttgtaggtggaccagttggtgattttgaacttttgctttgccacggaacggtctgcgttgtc<br>gggaagatgcgtgatctgatccttcaactcagcaaaagttcgatttattcaacaaagccacgttgtgtctcaaaatctctga<br>tgttacattgcacaagataaaaatatatcatcatgaacaataaaactgtctgcttacataaacagtaatacaaggggtgttt<br>actagaggagattctcatgtttgacagcttatcatcgataagctttaatgcggtagtttatcacagttaaattgctaacgcagt<br>caggcaccgtgtatgaaatctaacaatgcgctcatcgtcattctcggcaccgtcaccctggacgctgtaggcataggctt<br>ggttatgccggtactgccgggcctcttgcgggatatcgtccattccgacagtattgccagtcactatggcgtgctgcttgcg<br>ctctatgcgttgatgcaatttctttgcgcacccgttctcggagccctgtccgaccgctttggccgccgtccagtcctgctcgctt<br>cgctccttggagccactatcgactacgcgatcatggcgaccacacccgtcctgtggattctctacgccggacgcatcgtg<br>gcgggcatcacgggtgccacaggtgcggttgctggtgcctatatcgccgacatcaccgatggggaagatcggctcgc<br>cacttcgggctcatgagcgcttgtttcggcgtgggtatggtggcaggccccgtggccgggggactgttgggtgccatctcc<br>ttgcatgcaccattccttgcggcggcggtgctcaacggcctcaacctcctcctgggctgcttccttatgcaggaatcgcata<br>agggagagcgccgtccgatgcccttgcgtgccttcaatccagtcagctccttccggtgggcgcggggcatgactatcgtc<br>gccgcacttatgactgttttctttatcatgcaactcgtaggacaggttccggcagcgctctgggtcattttcggcgaggaccg<br>ctttcgctggagcgcgacgatgatcggcctgtcgcttgcggtattcggaatcttgcacgccctcgctcaagccttcgtcacg<br>ggccccgccaccaaacgtttcggcgagaagcaggccattatcgcgggcatggcggccgacgcgctgggctacgtctt<br>gctggcgttcgcgacgcgcggctggatggccttccccattatgattcttctcgcttccggcggcatcggtatgcccgcgttg<br>caggccatgctgtcccgccaagtagatgacgaccatcagggacagcttcaagggtcgctcgcggctcttaccagcctc<br>acttcgatcattggaccgctgatcgtcacggcgatttatgccgcctcggcgagcacatggaacgggttggcatggattgta<br>ggtgccgccctttaccttgtctgcctccccgcgttgcgtcgcggtgcatggagccgggccacctcgacctaataatactag<br>ctccggcaaaaaaacgggcaaggtgtcaccaccctgcccttttctttaaaaccgaaaagattacttcgcgtt

Sequence name	*Dalbergia maritima* DmMatK Toehold Switch 1.3 Trigger
Acc. number	BBa_K3453122
Sequence	tgccacctgacgtctaagaaaccattattatcatgacattaacctataaaaataggcgtatcacgaggcagaatttcagat<br>aaaaaaaatccttagctttcgctaaggatgatttctggaattcgcggccgcttctagagtaatacgactcactatagggtgg<br>cttcaaagaatgcgcctcttttgatgaataaatggaaacactatctcatctatttctggcaatgtcattttgatgtttggtctcaa<br>cctggaacgatccatataaatccattattattactcgaacccctagcccgctcttatcgggcggctaggggtttttttgttacta<br>gtagcggccgctgcagtccggcaaaaaagggcaaggtgtcaccaccctgcccttttctttaaaaccgaaaagattactt<br>cgcgttatgcaggcttcctcgctcactgactcgctgcgctcggtcgttcggctgcggcgagcggtatcagctcactcaaag<br>gcggtaatacggttatccacagaatcaggggataacgcaggaaagaacatgtgagcaaaaggccagcaaaaggcc<br>aggaaccgtaaaaaggccgcgttgctggcgtttttccacaggctccgcccccctgacgagcatcacaaaaatcgacgc<br>tcaagtcagaggtggcgaaacccgacaggactataaagataccaggcgtttccccctggaagctccctcgtgcgctctc<br>ctgttccgaccctgccgcttaccggatacctgtccgcctttctcccttcgggaagcgtggcgctttctcatagctcacgctgta<br>ggtatctcagttcggtgtaggtcgttcgctccaagctgggctgtgtgcacgaaccccccgttcagcccgaccgctgcgcct<br>tatccggtaactatcgtcttgagtccaacccggtaagacacgacttatcgccactggcagcagccactggtaacaggatt<br>agcagagcgaggtatgtaggcggtgctacagagttcttgaagtggtggcctaactacggctacactagaagaacagta<br>tttggtatctgcgctctgctgaagccagttaccttcggaaaaagagttggtagctcttgatccggcaaacaaaccaccgct<br>ggtagcggtggtttttttgtttgcaagcagcagattacgcgcagaaaaaaaggatctcaagaagatcctttgatcttttctac<br>ggggtctgacgctcagtggaacgaaaactcacgttaagggattttggtcatgagattatcaaaaaggatcttcacctaga<br>tccttttaaattaaaaatgaagttttaaatcaatctaaagtatatatgagtaaacttggtctgacagctcgaggcttggattctc<br>accaataaaaaacgcccggcggcaaccgagcgttctgaacaaatccagatggagttctgaggtcattactggatctatc<br>aacaggagtccaagcgagctcgatatcaaattacgccccgccctgccactcatcgcagtactgttgtaattcattaagca<br>ttctgccgacatggaagccatcacaaacggcatgatgaacctgaatcgccagcggcatcagcaccttgtcgccttgcgt

	ataatatttgcccatggtgaaaacgggggcgaagaagttgtccatattggccacgtttaaatcaaaactggtgaaactca cccagggattggctgacacgaaaaacatattctcaataaaccctttagggaaataggccaggttttcaccgtaacacgc cacatcttgcgaatatatgtgtagaaactgccggaaatcgtcgtggtattcactccagagcgatgaaaacgtttcagtttgc tcatggaaaacggtgtaacaagggtgaacactatcccatatcaccagctcaccgtctttcattgccatacgaaattccgg atgagcattcatcaggcgggcaagaatgtgaataaaggccggataaaacttgtgcttatttttctttacggtctttaaaaag gccgtaatatccagctgaacggtctggttataggtacattgagcaactgactgaaatgcctcaaaatgttctttacgatgcc attgggatatatcaacggtggtatatccagtgatttttttctccattttagcttccttagctcctgaaaatctcgataactcaaaa aatacgcccggtagtgatcttatttcattatggtgaaagttggaacctcttacgtgcccgatcaactcgag

Sequence name	*Dalbergia maritima* DmMatK Toehold Switch 1.4 Sensor
Acc. number	BBa_K3453113
Sequence	tgccacctgacgtctaagaaaaggaatattcagcaatttgcccgtgccgaagaaaggcccacccgtgaaggtgagcc agtgagttgattgctacgtaattagttagttagcccttagtgactggaattcgcggccgcttctagagtaatacgactcactat agggccaaacatcaaaatgacattgccagaaatagatgatatggactttagaacagaggagataaagatgatatcaat gatttctggcaacctggcggcagcgcaaaagatgcgtaaaggcgaagaactgtttaccggtgtggttccgattctggtgg aactggacggcgatgttaatggtcataaattcagtgttcgcggcgaaggtgaaggcgatgcgacgaacggcaaactg accctgaaatttatctgcaccacgggtaaactgccggtcccgtggccgacgctggtgaccacgctgacctatggcgttca atgttttgcgcgttacccggatcacatgaaacagcacgactttttcaaatcggccatgccggaaggctatgtgcaggaac gtacgattagctttaaagacgatggtacgtataaaacccgcgcggaagtgaaattcgaaggcgataccctggttaaccg tatcgaactgaaaggtatcgatttcaaagaagatggcaatattctgggtcataaactggaatataacttcaattcccacaa cgtgtacatcaccgcggataaacagaaaaacggcattaaagccaatttcaaaatccgccataatgtggaagatggtag cgttcagctggccgaccactatcagcaaaacacgccgattggtgatggcccggtcctgctgccggacaatcactacctg agtacccagtccgtgctgtcaaaagatccgaacgaaaaacgtgaccacatggtcctgctggaatttgtgacggctgcgg gtatcacccacggcatggacgaactgtataaaggcctgctgcaaacgacgaaaactacgctttagtagcttaataata ctcgaacccctagcccgctcttatcgggcggctagggggtttttttgttactagtagcggccgctgcaggagtcactaagggtt agttagttagattagcagaaagtcaaaagcctccgaccggaggcttttgactaaaacttcccttggggttatcattggggct cactcaaaggcggtaatcagataaaaaaaatccttagctttcgctaaggatgatttctgctagagatggaatagactgga tggaggcggataaagttgcaggaccacttctgcgctcggcccttccggctggctggtttattgctgataaatctggagccg gtgagcgtgggactcgcggtatcattgcagcactggggccagatggtaagccctcccgtatcgtagttatctacacgacg gggagtcaggcaactatggatgaacgaaatagacagatcgctgagataggtgcctcactgattaagcattggtaactgt cagaccaagtttactcatatatactttagattgatttaaaacttcatttttaatttaaaaggatctaggtgaagatcctttttgata atctcatgaccaaaatcccttaacgtgagttttcgttccactgagcgtcagaccccttaataagatgatcttcttgagatcgttt tggtctgcgcgtaatctcttgctctgaaaacgaaaaaaccgccttgcagggcggtttttcgaaggttctctgagctaccaac tctttgaaccgaggtaactggcttggaggagcgcagtcaccaaaacttgtcctttcagtttagccttaaccggcgcatgact tcaagactaactcctctaaatcaattaccagtggctgctgccagtggtgcttttgcatgtctttccgggttggactcaagacg atagttaccggataaggcgcagcggtcggactgaacggggggttcgtgcatacagtccagcttggagcgaactgccta cccggaactgagtgtcaggcgtggaatgagacaaacgcggccataacagcggaatgacaccggtaaaccgaaag gcaggaacaggagagcgcacgagggagccgccaggggaaacgcctggtatctttatagtcctgtcgggtttcgccac cactgatttgagcgtcagatttcgtgatgcttgtcaggggggcggagcctatggaaaaacggctttgccgcggccctctca cttccctgttaagtatcttcctggcatcttccaggaaatctccgccccgttcgtaagccatttccgctcgccgcagtcgaacg accgagcgtagcgagtcagtgagcgaggaagcggaatatatcctgtatcacatattctgctgacgcaccggtgcagcct tttttctcctgccacatgaagcacttcactgacaccctcatcagtgccaacatagtaagccagtatacactccgctagcgct gaggtctgcctcgtgaagaaggtgttgctgactcataccaggcctgaatcgccccatcatccagccagaaagtgaggg agccacggttgatgagagctttgttgtaggtggaccagttggtgattttgaacttttgctttgccacggaacggtctgcgttgtc gggaagatgcgtgatctgatccttcaactcagcaaaagttcgatttattcaacaaagccacgttgtgtctcaaaatctctga tgttacattgcacaagataaaaatatatcatcatgaacaataaaactgtctgcttacataaacagtaatacaaggggtgttt actagaggagattctcatgtttgacagcttatcatcgataagctttaatgcggtagtttatcacagttaaattgctaacgcagt caggcaccgtgtatgaaatctaacaatgcgctcatcgtcatcctcggcaccgtcaccctggacgctgtaggcataggctt ggttatgccggtactgccgggcctcttgcgggatatcgtccattccgacagtattgccagtcactatggcgtgctgcttgcg ctctatgcgttgatgcaatttctttgcgcacccgttctcggagccctgtccgaccgctttggccgccgtccagtcctgctcgctt cgctccttggagccactatcgactacgcgatcatggcgaccacacccgtcctgtggattctctacgccggacgcatcgtg gcgggcatcacgggtgccacaggtgcggttgctggtgcctatatcgccgacatcaccgatggggaagatcgggctcgc cacttcgggctcatgagcgcttgtttcggcgtgggtatggtggcaggccccgtggccgggggactgttgggtgccatctcc ttgcatgcaccattccttgcggcggcggtgctcaacggcctcaacctcctcctgggctgcttcctaatgcaggaatcgcata agggagagcgccgtccgatgcccttgcgtgccttcaatccagtcagctccttccggtgggcgcggggcatgactatcgtc gccgcacttatgactgtttcttttatcatgcaactcgtaggacaggttccggcagcgctctgggtcatttcggcgaggaccg ctttcgctggagcgcgacgatgatcggcctgtcgcttgcggtattcggaatcttgcacgccctcgctcaagccttcgtcacg ggccccgccaccaaacgtttcggcgagaagcaggccattatcgcgggcatggcggccgacgcgctgggctacgtctt gctggcgttcgcgacgcgcggctggatggccttccccattatgattcttctcgcttccggcggcatcggtatgcccgcgttg

	caggccatgctgtcccgccaagtagatgacgaccatcagggacagcttcaagggtcgctcgcggctcttaccagcctc<br>acttcgatcattggaccgctgatcgtcacggcgatttatgccgcctcggcgagcacatggaacgggttggcatggattgta<br>ggtgccgccctttaccttgtctgcctccccgcgttgcgtcgcggtgcatggagccgggccacctcgacctaataatactag<br>ctccggcaaaaaaacgggcaaggtgtcaccaccctgccctttttctttaaaaccgaaaagattacttcgcgtt

Sequence name	*Dalbergia maritima* DmMatK Toehold Switch 1.4 Trigger
Acc. number	BBa_K3453123
Sequence	tgccacctgacgtctaagaaaccattattatcatgacattaacctataaaaataggcgtatcacgaggcagaatttcagat<br>aaaaaaaatccttagctttcgctaaggatgatttctggaattcgcggccgcttctagagtaatacgactcactatagggtca<br>tctatttctggcaatgtcattttgatgtttggtctcaacctggaacgatccatataaatccattattatccgagcattcatttcactt<br>tttttggggggctatctttcaaatgtgctactcgaacccctagcccgctcttatcgggcggctaggggtttttttgttactagta<br>gcggccgctgcagtccggcaaaaaagggcaaggtgtcaccaccctgccctttttctttaaaaccgaaaagattacttcg<br>cgttatgcaggcttcctcgctcactgactcgctgcgctcggtcgttcggctgcggcgagcggtatcagctcactcaaaggc<br>ggtaatacggttatccacagaatcaggggataacgcaggaaagaacatgtgagcaaaaggccagcaaaaggcca<br>ggaaccgtaaaaaggccgcgttgctggcgtttttccacaggctccgcccccctgacgagcatcacaaaaatcgacgct<br>caagtcagaggtggcgaaacccgacaggactataaagataccaggcgtttccccctggaagctccctcgtgcgctctc<br>ctgttccgaccctgccgcttaccggatacctgtccgcctttctcccttcgggaagcgtggcgctttctcatagctcacgctgta<br>ggtatctcagttcggtgtaggtcgttcgctccaagctgggctgtgtgcacgaaccccccgttcagcccgaccgctgcgcct<br>tatccggtaactatcgtcttgagtccaacccggtaagacacgacttatcgccactggcagcagccactggtaacaggatt<br>agcagagcgaggtatgtaggcggtgctacagagttcttgaagtggtggcctaactacggctacactagaagaacagta<br>tttggtatctgcgctctgctgaagccagttaccttcggaaaaagagttggtagctcttgatccggcaaacaaaccaccgct<br>ggtagcggtggtttttttgtttgcaagcagcagattacgcgcagaaaaaaaggatctcaagaagatcctttgatcttttctac<br>ggggtctgacgctcagtggaacgaaaactcacgttaagggattttggtcatgagattatcaaaaaggatcttcacctaga<br>tccttttaaattaaaaatgaagttttaaatcaatctaaagtatatatgagtaaacttggtctgacagctcgaggcttggattctc<br>accaataaaaaacgcccggcggcaaccgagcgttctgaacaaatccagatggagttctgaggtcattactggatctatc<br>aacaggagtccaagcgagctcgatatcaaattacgccccgccctgccactcatcgcagtactgttgtaattcattaagca<br>ttctgccgacatggaagccatcacaaacggcatgatgaacctgaatcgccagcggcatcagcaccttgtcgccttgcgt<br>ataatatttgcccatggtgaaaacgggggcgaagaagttgtccatattggccacgtttaaatcaaaactggtgaaactca<br>cccagggattggctgacacgaaaaacatattctcaataaaccctttagggaaataggccaggttttcaccgtaacacgc<br>cacatcttgcgaatatatgtgtagaaactgccggaaatcgtcgtggtattcactccagagcgatgaaaacgtttcagtttgc<br>tcatggaaaacggtgtaacaagggtgaacactatcccatatcaccagctcaccgtctttcattgccatacgaaattccgg<br>atgagcattcatcaggcgggcaagaatgtgaataaaggccggataaaacttgtgcttatttttctttacggtctttaaaaag<br>gccgtaatatccagctgaacggtctggttataggtacattgagcaactgactgaaatgcctcaaaatgttctttacgatgcc<br>attgggatatatcaacggtggtatatccagtgatttttttctccattttagcttccttagctcctgaaaatctcgataactcaaaa<br>aatacgcccggtagtgatcttatttcattatggtgaaagttggaacctcttacgtgcccgatcaactcgag

Sequence name	*Dalbergia maritima* DmRbcL Toehold Switch 1.1 Sensor
Acc. number	BBa_K3453131
Sequence	tgccacctgacgtctaagaaaaggaatattcagcaatttgcccgtgccgaagaaaggcccacccgtgaaggtgagcc<br>agtgagttgattgctacgtaattagttagttagcccttagtgactggaattcgcggccgcttctagagtaatacgactcactat<br>agggaaagttttaatataagaagtagggattcgcaaatataggactttagaacagaggagataaagatgtatattatgg<br>aatccctaaacctggcggcagcgcaaaagatgcgtaaaggcgaagaactgtttaccggtgtggttccgattctggtgga<br>actggacggcgatgttaatggtcataaattcagtgttcgcggcgaaggtgaaggcgatgcgacgaacggcaaactgac<br>cctgaaatttatctgcaccacgggtaaactgccggtcccgtggccgacgctggtgaccacgctgacctatggcgttcaat<br>gttttgcgcgttacccggatcacatgaaacagcacgactttttcaaatcggccatgccggaaggctatgtgcaggaacgt<br>acgattagctttaaagacgatggtacgtataaaacccgcgcggaagtgaaattcgaaggcgataccctggttaaccgta<br>tcgaactgaaaggtatcgatttcaaagaagatggcaatattctgggtcataaactggaatataacttcaattcccacaacg<br>tgtacatcaccgcggataaacagaaaaacggcattaaagccaatttcaaaatccgccataatgtggaagatggtagcg<br>ttcagctggccgaccactatcagcaaaacacgccgattggtgatggcccggtcctgctgccggacaatcactacctgag<br>tacccagtccgtgctgtcaaaagatccgaacgaaaaacgtgaccacatggtcctgctggaatttgtgacggctgcgggt<br>atcacccacggcatggacgaactgtataaaggcctgctgcaaacgacgaaaactacgctttagtagcttaataatact<br>cgaacccctagcccgctcttatcgggcggctaggggtttttttgttactagtagcggccgctgcaggagtcactaagggtta<br>gttagttagattagcagaaagtcaaaagcctccgaccggaggcttttgactaaaacttcccttggggttatcattggggctc<br>actcaaaggcggtaatcagataaaaaaaatccttagctttcgctaaggatgatttctgctagagatggaatagactggat<br>ggaggcggataaagttgcaggaccacttctgcgctcggcccttccggctggctggtttattgctgataaatctggagccgg<br>tgagcgtgggactcgcggtatcattgcagcactggggccagatggtaagccctcccgtatcgtagttatctacacgacgg<br>ggagtcaggcaactatggatgaacgaaatagacagatcgctgagataggtgcctcactgattaagcattggtaactgtc<br>agaccaagtttactcatatatactttagattgatttaaaacttcatttttaatttaaaaggatctaggtgaagatcctttttgataat

	ctcatgaccaaaatcccttaacgtgagttttcgttccactgagcgtcagaccccttaataagatgatcttcttgagatcgttttg<br>gtctgcgcgtaatctcttgctctgaaaacgaaaaaaccgccttgcagggcggtttttcgaaggttctctgagctaccaactc<br>tttgaaccgaggtaactggcttggaggagcgcagtcaccaaaacttgtcctttcagtttagccttaaccggcgcatgacttc<br>aagactaactcctctaaatcaattaccagtggctgctgccagtggtgcttttgcatgtctttccgggttggactcaagacgat<br>agttaccggataaggcgcagcggtcggactgaacggggggttcgtgcatacagtccagcttggagcgaactgcctacc<br>cggaactgagtgtcaggcgtggaatgagacaaacgcggccataacagcggaatgacaccggtaaaccgaaaggc<br>aggaacaggagagcgcacgagggagccgccaggggaaacgcctggtatctttatagtcctgtcgggtttcgccacca<br>ctgatttgagcgtcagatttcgtgatgcttgtcaggggggcggagcctatggaaaaacggctttgccgcggccctctcactt<br>ccctgttaagtatcttcctggcatcttccaggaaatctccgccccgttcgtaagccatttccgctcgccgcagtcgaacgac<br>cgagcgtagcgagtcagtgagcgaggaagcggaatatatcctgtatcacatattctgctgacgcaccggtgcagcctttt<br>ttctcctgccacatgaagcacttcactgacaccctcatcagtgccaacatagtaagccagtatacactccgctagcgctg<br>aggtctgcctcgtgaagaaggtgttgctgactcataccaggcctgaatcgccccatcatccagccagaaagtgaggga<br>gccacggttgatgagagctttgttgtaggtggaccagttggtgattttgaacttttgctttgccacggaacggtctgcgttgtcg<br>ggaagatgcgtgatctgatccttcaactcagcaaaagttcgatttattcaacaaagccacgttgtgtctcaaaatctctgat<br>gttacattgcacaagataaaaatatatcatcatgaacaataaaactgtctgcttacataaacagtaatacaaggggtgttt<br>actagaggagattctcatgtttgacagcttatcatcgataagctttaatgcggtagtttatcacagttaaattgctaacgcagt<br>caggcaccgtgtatgaaatctaacaatgcgctcatcgtcattctcggcaccgtcaccctggacgctgtaggcataggctt<br>ggttatgccggtactgccgggcctcttgcgggatatcgtccattccgacagtattgccagtcactatggcgtgctgcttgcg<br>ctctatgcgttgatgcaatttctttgcgcacccgttctcggagccctgtccgaccgctttggccgccgtccagtcctgctcgctt<br>cgctccttggagccactatcgactacgcgatcatggcgaccacacccgtcctgtggattctctacgccggacgcatcgtg<br>gcgggcatcacgggtgccacaggtgcggttgctggtgcctatatcgccgacatcaccgatggggaagatcgggctcgc<br>cacttcgggctcatgagcgcttgtttcggcgtgggtatggtggcaggccccgtggccgggggactgttgggtgccatctcc<br>ttgcatgcaccattccttgcggcggcggtgctcaacggcctcaacctcctcctgggctgcttccttatgcaggaatcgcata<br>agggagagcgccgtccgatgcccttgcgtgccttcaatccagtcagctccttccggtgggcgcggggcatgactatcgtc<br>gccgcacttatgactgttttctttatcatgcaactcgtaggacaggttccggcagcgctctgggtcattttcggcgaggaccg<br>ctttcgctggagcgcgacgatgatcggcctgtcgcttgcggtattcggaatcttgcacgccctcgctcaagccttcgtcacg<br>ggccccgccaccaaacgtttcggcgagaagcaggccattatcgcgggcatggcggccgacgcgctgggctacgtctt<br>gctggcgttcgcgacgcgcggctggatggccttccccattatgattcttctcgcttccggcggcatcggtatgcccgcgttg<br>caggccatgctgtcccgccaagtagatgacgaccatcagggacagcttcaagggtcgctcgcggctcttaccagcctc<br>acttcgatcattggaccgctgatcgtcacggcgatttatgccgcctcggcgagcacatggaacgggttggcatggattgta<br>ggtgccgccctttaccttgtctgcctccccgcgttgcgtcgcggtgcatggagccgggccacctcgacctaataatactag<br>ctccggcaaaaaaacgggcaaggtgtcaccaccctgcccttttctttaaaaccgaaaagattacttcgcgtt

Sequence name	*Dalbergia maritima* DmRbcL Toehold Switch 1.1 Trigger
Acc. number	BBa_K3453141
Sequence	tgccacctgacgtctaagaaaccattattatcatgacattaacctataaaaataggcgtatcacgaggcagaatttcagat<br>aaaaaaaatccttagctttcgctaaggatgatttctggaattcgcggccgcttctagagtaatacgactcactatagggattt<br>gcgaatccctacttcttatattaaaactttccaaggtccgcctcacggtatccaagttgaaagagataaattaaacaagtat<br>ggccgtccactattgggatgtactattaaaccgatactcgaacccctagcccgctcttatcgggcggctaggggtttttttgtt<br>actagtagcggccgctgcagtccggcaaaaaagggcaaggtgtcaccaccctgcccttttctttaaaaccgaaaagat<br>tacttcgcgttatgcaggcttcctcgctcactgactcgctgcgctcggtcgttcggctgcggcgagcggtatcagctcactc<br>aaaggcggtaatacggttatccacagaatcaggggataacgcaggaaagaacatgtgagcaaaaggccagcaaa<br>aggccaggaaccgtaaaaaggccgcgttgctggcgtttttccacaggctccgcccccctgacgagcatcacaaaaatc<br>gacgctcaagtcagaggtggcgaaacccgacaggactataaagataccaggcgtttccccctggaagctccctcgtg<br>cgctctcctgttccgaccctgccgcttaccggatacctgtccgcctttctcccttcgggaagcgtggcgctttctcatagctca<br>cgctgtaggtatctcagttcggtgtaggtcgttcgctccaagctgggctgtgtgcacgaaccccccgttcagcccgaccgc<br>tgcgccttatccggtaactatcgtcttgagtccaacccggtaagacacgacttatcgccactggcagcagccactggtaa<br>caggattagcagagcgaggtatgtaggcggtgctacagagttcttgaagtggtggcctaactacggctacactagaag<br>aacagtatttggtatctgcgctctgctgaagccagttaccttcggaaaaagagttggtagctcttgatccggcaaacaaac<br>caccgctggtagcggtggtttttttgtttgcaagcagcagattacgcgcagaaaaaaaggatctcaagaagatcctttgat<br>cttttctacggggtctgacgctcagtggaacgaaaactcacgttaagggattttggtcatgagattatcaaaaaggatcttc<br>acctagatccttttaaattaaaaatgaagttttaaatcaatctaaagtatatatgagtaaacttggtctgacagctcgaggctt<br>ggattctcaccaataaaaaacgcccggcggcaaccgagcgttctgaacaaatccagatggagttctgaggtcattactg<br>gatctatcaacaggagtccaagcgagctcgatatcaaattacgccccgccctgccactcatcgcagtactgttgtaattca<br>ttaagcattctgccgacatggaagccatcacaaacggcatgatgaacctgaatcgccagcggcatcagcaccttgtcg<br>ccttgcgtataatatttgcccatggtgaaaacgggggcgaagaagttgtccatattggccacgtttaaatcaaaactggtg<br>aaactcacccagggattggctgagacgaaaaacatattctcaataaaccctttagggaaataggccaggttttcaccgt<br>aacacgccacatcttgcgaatatatgtgtagaaactgccggaaatcgtcgtggtattcactccagagcgatgaaaacgtt<br>tcagtttgctcatggaaaacggtgtaacaagggtgaacactatcccatatcaccagctcaccgtctttcattgccatacga

	aattccggatgagcattcatcaggcgggcaagaatgtgaataaaggccggataaaacttgtgcttatttttctttacggtctt<br>taaaaaggccgtaatatccagctgaacggtctggttataggtacattgagcaactgactgaaatgcctcaaaatgttcttta<br>cgatgccattgggatatatcaacggtggtatatccagtgatttttttctccattttagcttccttagctcctgaaaatctcgataa<br>ctcaaaaaatacgcccggtagtgatcttatttcattatggtgaaagttggaacctcttacgtgcccgatcaactcgag

Sequence name	*Dalbergia maritima* DmRbcL Toehold Switch 1.2 Sensor
Acc. number	BBa_K3453132
Sequence	tgccacctgacgtctaagaaaaggaatattcagcaatttgcccgtgccgaagaaaggcccacccgtgaaggtgagcc<br>agtgagttgattgctacgtaattagttagttagcccttagtgactggaattcgcggccgcttctagagtaatacgactcactat<br>agggataagaagtagggattcgcaaatcttccagacgtatatggactttagaacagaggagataaagatgatatacat<br>gtggaagattaacctggcggcagcgcaaaagatgcgtaaaggcgaagaactgtttaccggtgtggttccgattctggtg<br>gaactggacggcgatgttaatggtcataaattcagtgttcgcggcgaaggtgaaggcgatgcgacgaacggcaaact<br>gaccctgaaatttatctgcaccacgggtaaactgccggtcccgtggccgacgctggtgaccacgctgacctatggcgttc<br>aatgttttgcgcgttacccggatcacatgaaacagcacgactttttcaaatcggccatgccggaaggctatgtgcaggaa<br>cgtacgattagctttaaagacgatggtacgtataaaacccgcgcggaagtgaaattcgaaggcgataccctggttaacc<br>gtatcgaactgaaaggtatcgatttcaaagaagatggcaatattctgggtcataaactggaatataacttcaattcccaca<br>acgtgtacatcaccgcggataaacagaaaaacggcattaaagccaatttcaaaatccgccataatgtggaagatggta<br>gcgttcagctggccgaccactatcagcaaaacacgccgattggtgatggcccggtcctgctgccggacaatcactacct<br>gagtacccagtccgtgctgtcaaaagatccgaacgaaaaacgtgaccacatggtcctgctggaatttgtgacggctgcg<br>ggtatcacccacggcatggacgaactgtataaaggcctgctgcaaacgacgaaaactacgctttagtagcttaataat<br>actcgaacccctagcccgctcttatcgggcggctaggggtttttgttactagtagcggccgctgcaggagtcactaaggg<br>ttagttagttagattagcagaaagtcaaaagcctccgaccggaggcttttgactaaaacttcccttggggttatcattgggg<br>ctcactcaaaggcggtaatcagataaaaaaaatccttagctttcgctaaggatgatttctgctagagatggaatagactg<br>gatggaggcggataaagttgcaggaccacttctgcgctcggcccttccggctggctggtttattgctgataaatctggagc<br>cggtgagcgtgggactcgcggtatcattgcagcactggggccagatggtaagccctcccgtatcgtagttatctacacga<br>cggggagtcaggcaactatggatgaacgaaatagacagatcgctgagataggtgcctcactgattaagcattggtaac<br>tgtcagaccaagtttactcatatatactttagattgatttaaaacttcatttttaatttaaaaggatctaggtgaagatcctttttga<br>taatctcatgaccaaaatcccttaacgtgagttttcgttccactgagcgtcagaccccttaataagatgatcttcttgagatcg<br>ttttggtctgcgcgtaatctcttgctctgaaaacgaaaaaaccgccttgcagggcggtttttcgaaggttctctgagctacca<br>actctttgaaccgaggtaactggcttggaggagcgcagtcaccaaaacttgtcctttcagtttagccttaaccggcgcatg<br>acttcaagactaactcctctaaatcaattaccagtggctgctgccagtggtgcttttgcatgtctttccgggttggactcaaga<br>cgatagttaccggataaggcgcagcggtcggactgaacggggggttcgtgcatacagtccagcttggagcgaactgc<br>ctacccggaactgagtgtcaggcgtggaatgagacaaacgcggccataacagcggaatgacaccggtaaaccgaa<br>aggcaggaacaggagagcgcacgagggagccgccaggggaaacgcctggtatctttatagtcctgtcgggtttcgcc<br>accactgatttgagcgtcagatttcgtgatgcttgtcaggggggcggagcctatggaaaaacggctttgccgcggccctct<br>cacttccctgttaagtatcttcctggcatcttccaggaaatctccgccccgttcgtaagccatttccgctcgccgcagtcgaa<br>cgaccgagcgtagcgagtcagtgagcgaggaagcggaatatatcctgtatcacatattctgctgacgcaccggtgcag<br>cctttttctcctgccacatgaagcacttcactgacaccctcatcagtgccaacatagtaagccagtatacactccgctagc<br>gctgaggtctgcctcgtgaagaaggtgttgctgactcataccaggcctgaatcgccccatcatccagccagaaagtgag<br>ggagccacggttgatgagagctttgttgtaggtggaccagttggtgattttgaacttttgctttgccacggaacggtctgcgtt<br>gtcgggaagatgcgtgatctgatccttcaactcagcaaaagttcgatttattcaacaaagccacgttgtgtctcaaaatctc<br>tgatgttacattgcacaagataaaaatatatcatcatgaacaataaaactgtctgcttacataaacagtaatacaaggggt<br>gtttactagaggagattctcatgtttgacagcttatcatcgataagctttaatgcggtagtttatcacagttaaattgctaacgc<br>agtcaggcaccgtgtatgaaatctaacaatgcgctcatcgtcattctcggcaccgtcaccctggacgctgtaggcatagg<br>cttggttatgccggtactgccgggcctcttgcgggatatcgtccattccgacagtattgccagtcactatggcgtgctgcttg<br>cgctctatgcgttgatgcaatttctttgcgcacccgttctcggagccctgtccgaccgctttggccgccgtccagtcctgctcg<br>cttcgctccttggagccactatcgactacgcgatcatggcgaccacacccgtcctgtggattctctacgccggacgcatcg<br>tggcgggcatcacgggtgccacaggtgcggttgctggtgcctatatcgccgacatcaccgatggggaagatcgggctc<br>gccacttcgggctcatgagcgcttgtttcggcgtgggtatggtggcaggccccgtggccgggggactgttgggtgccatct<br>ccttgcatgcaccattccttgcggcggcggtgctcaacggcctcaacctcctcctgggctgcttcctaatgcaggaatcgca<br>taagggagagcgccgtccgatgcccttgcgtgccttcaatccagtcagctccttccggtgggcgcggggcatgactatcg<br>tcgccgcacttatgactgttttctttatcatgcaactcgtaggacaggttccggcagcgctctgggtcattttcggcgaggac<br>cgctttcgctggagcgcgacgatgatcggcctgtcgcttgcggtattcggaatcttgcacgccctcgctcaagccttcgtca<br>cgggccccgccaccaaacgtttcggcgagaagcaggccattatcgcgggcatggcggccgacgcgctgggctacgt<br>cttgctggcgttcgcgacgcgcggctggatggccttccccattatgattcttctcgcttccggcggcatcggtatgcccgcgt<br>tgcaggccatgctgtcccgccaagtagatgacgaccatcagggacagcttcaagggtcgctcgcggctcttaccagcct<br>cacttcgatcattggaccgctgatcgtcacggcgatttatgccgcctcggcgagcacatggaacgggttggcatggattgt<br>aggtgccgccctttaccttgtctgcctccccgcgttgcgtcgcggtgcatggagccgggccacctcgacctaataatacta<br>gctccggcaaaaaaacgggcaaggtgtcaccaccctgcccttttttctttaaaaccgaaaagattacttcgcgtt

Sequence name	*Dalbergia maritima* DmRbcL Toehold Switch 1.2 Trigger
Acc. number	BBa_K3453142
Sequence	tgccacctgacgtctaagaaaccattattatcatgacattaacctataaaaataggcgtatcacgaggcagaatttcagat aaaaaaaatccttagctttcgctaaggatgatttctggaattcgcggccgcttctagagtaatacgactcactatagggtac gtctggaagatttgcgaatccctacttcttatattaaaactttccaaggtccgcctcacggtatccaagttgaaagagataa attaaacaagtatggccgtccactattgggatgtatactcgaacccctagcccgctcttatcgggcggctaggggtttttgtt actagtagcggccgctgcagtccggcaaaaaagggcaaggtgtcaccaccctgccctttttctttaaaaccgaaaagat tacttcgcgttatgcaggcttcctcgctcactgactcgctgcgctcggtcgttcggctgcggcgagcggtatcagctcactc aaaggcggtaatacggttatccacagaatcaggggataacgcaggaaagaacatgtgagcaaaaggccagcaaa aggccaggaaccgtaaaaaggccgcgttgctggcgtttttccacaggctccgcccccctgacgagcatcacaaaaatc gacgctcaagtcagaggtggcgaaacccgacaggactataaagataccaggcgtttccccctggaagctccctcgtg cgctctcctgttccgaccctgccgcttaccggatacctgtccgcctttctcccttcgggaagcgtggcgctttctcatagctca cgctgtaggtatctcagttcggtgtaggtcgttcgctccaagctgggctgtgtgcacgaaccccccgttcagcccgaccgc tgcgccttatccggtaactatcgtcttgagtccaacccggtaagacacgacttatcgccactggcagcagccactggtaa caggattagcagagcgaggtatgtaggcggtgctacagagttcttgaagtggtggcctaactacggctacactagaag aacagtatttggtatctgcgctctgctgaagccagttaccttcggaaaaagagttggtagctcttgatccggcaaacaaac caccgctggtagcggtggtttttttgtttgcaagcagcagattacgcgcagaaaaaaaggatctcaagaagatcctttgat cttttctacggggtctgacgctcagtggaacgaaaactcacgttaagggattttggtcatgagattatcaaaaaggatcttc acctagatccttttaaattaaaaatgaagttttaaatcaatctaaagtatatatgagtaaacttggtctgacagctcgaggctt ggattctcaccaataaaaaacgcccggcggcaaccgagcgttctgaacaaatccagatggagttctgaggtcattactg gatctatcaacaggagtccaagcgagctcgatatcaaattacgccccgccctgccactcatcgcagtactgttgtaattca ttaagcattctgccgacatggaagccatcacaaacggcatgatgaacctgaatcgccagcggcatcagcaccttgtcg ccttgcgtataatatttgcccatggtgaaaacgggggcgaagaagttgtccatattggccacgtttaaatcaaaactggtg aaactcacccagggattggctgagacgaaaaacatattctcaataaaccctttagggaaataggccaggttttcaccgt aacacgccacatcttgcgaatatatgtgtagaaactgccggaaatcgtcgtggtattcactccagagcgatgaaaacgtt tcagtttgctcatggaaaacggtgtaacaagggtgaacactatcccatatcaccagctcaccgtctttcattgccatacga aattccggatgagcattcatcaggcgggcaagaatgtgaataaaggccggataaaacttgtgcttatttttctttacggtctt taaaaaggccgtaatatccagctgaacggtctggttataggtacattgagcaactgactgaaatgcctcaaaatgttcttta cgatgccattgggatatatcaacggtggtatatccagtgatttttttctccattttagcttccttagctcctgaaaatctcgataa ctcaaaaaatacgcccggtagtgatcttatttcattatggtgaaagttggaacctcttacgtgcccgatcaactcgag

Sequence name	*Dalbergia maritima* DmRbcL Toehold Switch 1.3 Sensor
Acc. number	BBa_K3453133
Sequence	tgccacctgacgtctaagaaaaggaatattcagcaatttgcccgtgccgaagaaaggcccacccgtgaaggtgagcc agtgagttgattgctacgtaattagttagttagcccttagtgactggaattcgcggccgcttctagagtaatacgactcactat agggacataagcaatatattgattctcttctccagcaacataggactttagaacagaggagataaagatgtatgttatggg agaagagaacctggcggcagcgcaaaagatgcgtaaaggcgaagaactgtttaccggtgtggttccgattctggtgg aactggacggcgatgttaatggtcataaattcagtgttcgcggcgaaggtgaaggcgatgcgacgaacggcaaactg accctgaaatttatctgcaccacgggtaaactgccggtcccgtggccgacgctggtgaccacgctgacctatggcgttca atgttttgcgcgttacccggatcacatgaaacagcacgactttttcaaatcggccatgccggaaggctatgtgcaggaac gtacgattagctttaaagacgatggtacgtataaaacccgcgcggaagtgaaattcgaaggcgataccctggttaaccg tatcgaactgaaaggtatcgatttcaaagaagatggcaatattctgggtcataaactggaatataacttcaattcccacaa cgtgtacatcaccgcggataaacagaaaaacggcattaaagccaatttcaaaatccgccataatgtggaagatggtag cgttcagctggccgaccactatcagcaaaacacgccgattggtgatggcccggtcctgctgccggacaatcactacctg agtacccagtccgtgctgtcaaaagatccgaacgaaaaacgtgaccacatggtcctgctggaatttgtgacggctgcgg gtatcacccacggcatggacgaactgtataaaggcctgctgcaaacgacgaaaactacgctttagtagcttaataata ctcgaacccctagcccgctcttatcgggcggctaggggtttttgttactagtagcggccgctgcaggagtcactaagggtt agttagttagattagcagaaagtcaaaagcctccgaccggaggcttttgactaaaacttcccttggggttatcattggggct cactcaaaggcggtaatcagataaaaaaaatccttagctttcgctaaggatgatttctgctagagatggaatagactgga tggaggcggataaagttgcaggaccacttctgcgctcggcccttccggctggctggtttattgctgataaatctggagccg gtgagcgtgggactcgcggtatcattgcagcactggggccagatggtaagccctcccgtatcgtagttatctacacgacg gggagtcaggcaactatggatgaacgaaatagacagatcgctgagataggtgcctcactgattaagcattggtaactgt cagaccaagtttactcatatatactttagattgatttaaaacttcatttttaatttaaaaggatctaggtgaagatcctttttgata atctcatgaccaaaatcccttaacgtgagttttcgttccactgagcgtcagaccccttaataagatgatcttcttgagatcgttt tggtctgcgcgtaatctcttgctctgaaaacgaaaaaaccgccttgcagggcggtttttcgaaggttctctgagctaccaac tctttgaaccgaggtaactggcttggaggagcgcagtcaccaaaacttgtcctttcagtttagccttaaccggcgcatgact tcaagactaactcctctaaatcaattaccagtggctgctgccagtggtgcttttgcatgtctttccgggttggactcaagacg atagttaccggataaggcgcagcggtcggactgaacggggggttcgtgcatacagtccagcttggagcgaactgccta

	cccggaactgagtgtcaggcgtggaatgagacaaacgcggccataacagcggaatgacaccggtaaaccgaaag<br>gcaggaacaggagagcgcacgagggagccgccaggggaaacgcctggtatctttatagtcctgtcgggtttcgccac<br>cactgatttgagcgtcagatttcgtgatgcttgtcaggggggcggagcctatggaaaaacggctttgccgcggccctctca<br>cttccctgttaagtatcttcctggcatcttccaggaaatctccgccccgttcgtaagccatttccgctcgccgcagtcgaacg<br>accgagcgtagcgagtcagtgagcgaggaagcggaatatatcctgtatcacatattctgctgacgcaccggtgcagcct<br>tttttctcctgccacatgaagcacttcactgacaccctcatcagtgccaacatagtaagccagtatacactccgctagcgct<br>gaggtctgcctcgtgaagaaggtgttgctgactcataccaggcctgaatcgccccatcatccagccagaaagtgaggg<br>agccacggttgatgagagctttgttgtaggtggaccagttggtgattttgaacttttgctttgccacggaacggtctgcgttgtc<br>gggaagatgcgtgatctgatccttcaactcagcaaaagttcgatttattcaacaaagccacgttgtgtctcaaaatctctga<br>tgttacattgcacaagataaaaatatatcatcatgaacaataaaactgtctgcttacataaacagtaatacaaggggtgttt<br>actagaggagattctcatgtttgacagcttatcatcgataagctttaatgcggtagtttatcacagttaaattgctaacgcagt<br>caggcaccgtgtatgaaatctaacaatgcgctcatcgtcattctcggcaccgtcaccctggacgctgtaggcataggctt<br>ggttatgccggtactgccgggcctcttgcgggatatcgtccattccgacagtattgccagtcactatggcgtgctgcttgcg<br>ctctatgcgttgatgcaatttctttgcgcacccgttctcggagccctgtccgaccgctttggccgccgtccagtcctgctcgctt<br>cgctccttggagccactatcgactacgcgatcatggcgaccacacccgtcctgtggattctctacgccggacgcatcgtg<br>gcgggcatcacgggtgccacaggtgcggttgctggtgcctatatcgccgacatcaccgatggggaagatcgggctcgc<br>cacttcgggctcatgagcgcttgtttcggcgtgggtatggtggcaggccccgtggccgggggactgttgggtgccatctcc<br>ttgcatgcaccattccttgcggcggcggtgctcaacggcctcaacctcctcctgggctgcttccttatgcaggaatcgcata<br>agggagagcgccgtccgatgcccttgcgtgccttcaatccagtcagctccttccggtgggcgcggggcatgactatcgtc<br>gccgcacttatgactgttttctttatcatgcaactcgtaggacaggttccggcagcgctctgggtcattttcggcgaggaccg<br>ctttcgctggagcgcgacgatgatcggcctgtcgcttgcggtattcggaatcttgcacgccctcgctcaagccttcgtcacg<br>ggccccgccaccaaacgtttcggcgagaagcaggccattatcgcgggcatggcggccgacgcgctgggctacgtctt<br>gctggcgttcgcgacgcgcggctggatggccttccccattatgattcttctcgcttccggcggcatcggtatgcccgcgttg<br>caggccatgctgtcccgccaagtagatgacgaccatcagggacagcttcaagggtcgctcgcggctcttaccagcctc<br>acttcgatcattggaccgctgatcgtcacggcgatttatgccgcctcggcgagcacatggaacgggttggcatggattgta<br>ggtgccgccctttaccttgtctgcctccccgcgttgcgtcgcggtgcatggagccgggccacctcgacctaataatactag<br>ctccggcaaaaaaacgggcaaggtgtcaccaccctgccctttttctttaaaaccgaaaagattacttcgcgtt

Sequence name	*Dalbergia maritima* DmRbcL Toehold Switch 1.3 Trigger
Acc. number	BBa_K3453143
Sequence	tgccacctgacgtctaagaaaccattattatcatgacattaacctataaaaataggcgtatcacgaggcagaatttcagat<br>aaaaaaaatccttagctttcgctaaggatgatttctggaattcgcggccgcttctagagtaatacgactcactatagggttg<br>ctggagaagagaatcaatatattgcttatgtagcttatcccttagaccttttttgaagaaggttctgttactaacatgtttacttcc<br>attgtaggtaatgtatttgggttcaaggcctactcgaacccctagcccgctcttatcgggcggctaggggtttttttgttactagt<br>agcggccgctgcagtccggcaaaaaagggcaaggtgtcaccaccctgccctttttctttaaaaccgaaaagattacttc<br>gcgttatgcaggcttcctcgctcactgactcgctgcgctcggtcgttcggctgcggcgagcggtatcagctcactcaaagg<br>cggtaatacggttatccacagaatcaggggataacgcaggaaagaacatgtgagcaaaaggccagcaaaaggcca<br>ggaaccgtaaaaaggccgcgttgctggcgtttttccacaggctccgcccccctgacgagcatcacaaaaatcgacgct<br>caagtcagaggtggcgaaacccgacaggactataaagataccaggcgtttccccctggaagctccctcgtgcgctctc<br>ctgttccgaccctgccgcttaccggatacctgtccgcctttctcccttcgggaagcgtggcgctttctcatagctcacgctgta<br>ggtatctcagttcggtgtaggtcgttcgctccaagctgggctgtgtgcacgaaccccccgttcagcccgaccgctgcgcct<br>tatccggtaactatcgtcttgagtccaacccggtaagacacgacttatcgccactggcagcagccactggtaacaggatt<br>agcagagcgaggtatgtaggcggtgctacagagttcttgaagtggtggcctaactacggctacactagaagaacagta<br>tttggtatctgcgctctgctgaagccagttaccttcggaaaaagagttggtagctcttgatccggcaaacaaaccaccgct<br>ggtagcggtggtttttttgtttgcaagcagcagattacgcgcagaaaaaaaggatctcaagaagatcctttgatcttttctac<br>ggggtctgacgctcagtggaacgaaaactcacgttaagggattttggtcatgagattatcaaaaaggatcttcacctaga<br>tccttttaaattaaaaatgaagttttaaatcaatctaaagtatatatgagtaaacttggtctgacagctcgaggcttggattctc<br>accaataaaaaacgcccggcggcaaccgagcgttctgaacaaatccagatggagttctgaggtcattactggatctatc<br>aacaggagtccaagcgagctcgatatcaaattacgccccgccctgccactcatcgcagtactgttgtaattcattaagca<br>ttctgccgacatggaagccatcacaaacggcatgatgaacctgaatcgccagcggcatcagcaccttgtcgccttgcgt<br>ataatatttgcccatggtgaaaacgggggcgaagaagttgtccatattggccacgtttaaatcaaaactggtgaaactca<br>cccagggattggctgagacgaaaaacatattctcaataaaccctttagggaaataggccaggttttcaccgtaacacgc<br>cacatcttgcgaatatatgtgtagaaactgccggaaatcgtcgtggtattcactccagagcgatgaaaacgtttcagtttgc<br>tcatggaaaacggtgtaacaagggtgaacactatcccatatcaccagctcaccgtctttcattgccatacgaaattccgg<br>atgagcattcatcaggcgggcaagaatgtgaataaaggccggataaaacttgtgcttatttttctttacggtctttaaaaag<br>gccgtaatatccagctgaacggtctggttataggtacattgagcaactgactgaaatgcctcaaaatgttctttacgatgcc<br>attgggatatatcaacggtggtatatccagtgatttttttctccattttagcttccttagctcctgaaaatctcgataactcaaaa<br>aatacgcccggtagtgatcttatttcattatggtgaaagttggaacctcttacgtgcccgatcaactcgag

Sequence name	*Dalbergia maritima* DmTrnL-UAA Toehold Switch 1.1 Sensor
Acc. number	BBa_K3453151
Sequence	tgccacctgacgtctaagaaaaggaatattcagcaatttgcccgtgccgaagaaaggcccacccgtgaaggtgagcc<br>agtgagttgattgctacgtaattagttagttagcccttagtgactggaattcgcggccgcttctagagtaatacgactcactat<br>agggaatagaaattgggagtcttcattaatcaattgaaatatggactttagaacagaggagataaagatgatatttatgttg<br>attaataacctggcggcagcgcaaaagatgcgtaaaggcgaagaactgtttaccggtgtggttccgattctggtggaact<br>ggacggcgatgttaatggtcataaattcagtgttcgcggcgaaggtgaaggcgatgcgacgaacggcaaactgaccct<br>gaaatttatctgcaccacgggtaaactgccggtcccgtggccgacgctggtgaccacgctgacctatggcgttcaatgttt<br>tgcgcgttacccggatcacatgaaacagcacgactttttcaaatcggccatgccggaaggctatgtgcaggaacgtacg<br>attagctttaaagacgatggtacgtataaaacccgcgcggaagtgaaattcgaaggcgataccctggttaaccgtatcg<br>aactgaaaggtatcgatttcaaagaagatggcaatattctgggtcataaactggaatataacttcaattcccacaacgtgt<br>acatcaccgcggataaacagaaaaacggcattaaagccaatttcaaaatccgccataatgtggaagatggtagcgttc<br>agctggccgaccactatcagcaaaacacgccgattggtgatggcccggtcctgctgccggacaatcactacctgagta<br>cccagtccgtgctgtcaaaagatccgaacgaaaaacgtgaccacatggtcctgctggaatttgtgacggctgcgggtat<br>cacccacggcatggacgaactgtataaaaggcctgctgcaaacgacgaaaactacgctttagtagcttaataatactcg<br>aacccctagcccgctcttatcgggcggctaggggtttttgttactagtagcggccgctgcaggagtcactaagggttagtt<br>agttagattagcagaaagtcaaaagcctccgaccggaggcttttgactaaaacttcccttggggttatcattggggctcac<br>tcaaaggcggtaatcagataaaaaaaatccttagctttcgctaaggatgatttctgctagagatggaatagactggatgg<br>aggcggataaagttgcaggaccacttctgcgctcggcccttccggctggctggtttattgctgataaatctggagccggtg<br>agcgtgggactcgcggtatcattgcagcactggggccagatggtaagccctcccgtatcgtagttatctacacgacggg<br>gagtcaggcaactatggatgaacgaaatagacagatcgctgagataggtgcctcactgattaagcattggtaactgtca<br>gaccaagtttactcatatatactttagattgatttaaaacttcatttttaatttaaaaggatctaggtgaagatcctttttgataatc<br>tcatgaccaaaatcccttaacgtgagttttcgttccactgagcgtcagaccccttaataagatgatcttcttgagatcgttttgg<br>tctgcgcgtaatctcttgctctgaaaacgaaaaaaccgccttgcagggcggtttttcgaaggttctctgagctaccaactctt<br>tgaaccgaggtaactggcttggaggagcgcagtcaccaaaacttgtcctttcagtttagccttaaccggcgcatgacttca<br>agactaactcctctaaatcaattaccagtggctgctgccagtggtgcttttgcatgtctttccgggttggactcaagacgata<br>gttaccggataaggcgcagcggtcggactgaacggggggttcgtgcatacagtccagcttggagcgaactgcctaccc<br>ggaactgagtgtcaggcgtggaatgagacaaacgcggccataacagcggaatgacaccggtaaaccgaaaggca<br>ggaacaggagagcgcacgagggagccgccaggggaaacgcctggtatctttatagtcctgtcgggtttcgccaccact<br>gatttgagcgtcagatttcgtgatgcttgtcaggggggcggagcctatggaaaaacggctttgccgcggccctctcacttc<br>cctgttaagtatcttcctggcatcttccaggaaatctccgccccgttcgtaagccatttccgctcgccgcagtcgaacgacc<br>gagcgtagcgagtcagtgagcgaggaagcggaatatatcctgtatcacatattctgctgacgcaccggtgcagcctttttt<br>ctcctgccacatgaagcacttcactgacaccctcatcagtgccaacatagtaagccagtatacactccgctagcgctga<br>ggtctgcctcgtgaagaaggtgttgctgactcataccaggcctgaatcgccccatcatccagccagaaagtgagggag<br>ccacggttgatgagagctttgttgtaggtggaccagttggtgattttgaacttttgctttgccacggaacggtctgcgttgtcgg<br>gaagatgcgtgatctgatccttcaactcagcaaaagttcgatttattcaacaaagccacgttgtgtctcaaaatctctgatgt<br>tacattgcacaagataaaaatatatcatcatgaacaataaaactgtctgcttacataaacagtaatacaaggggtgtttac<br>tagaggagattctcatgtttgacagcttatcatcgataagctttaatgcggtagtttatcacagttaaattgctaacgcagtca<br>ggcaccgtgtatgaaatctaacaatgcgctcatcgtcattctcggcaccgtcaccctggacgctgtaggcataggcttggt<br>tatgccggtactgccgggcctcttgcgggatatcgtccattccgacagtattgccagtcactatggcgtgctgcttgcgctct<br>atgcgttgatgcaatttctttgcgcacccgttctcggagccctgtccgaccgctttggccgccgtccagtcctgctcgcttcgc<br>tccttggagccactatcgactacgcgatcatggcgaccacacccgtcctgtggattctctacgccggacgcatcgtggcg<br>ggcatcacgggtgccacaggtgcggttgctggtgcctatatcgccgacatcaccgatggggaagatcgggctcgccac<br>ttcgggctcatgagcgcttgtttcggcgtgggtatggtggcaggccccgtggccgggggactgttgggtgccatctccttgc<br>atgcaccattccttgcggcggcggtgctcaacggcctcaacctcctcctgggctgcttccttatgcaggaatcgcataagg<br>gagagcgccgtccgatgcccttgcgtgccttcaatccagtcagctccttccggtgggcgcggggcatgactatcgtcgcc<br>gcacttatgactgttttctttatcatgcaactcgtaggacaggttccggcagcgctctgggtcattttcggcgaggaccgcttt<br>cgctggagcgcgacgatgatcggcctgtcgcttgcggtattcggaatcttgcacgccctcgctcaagccttcgtcacggg<br>ccccgccaccaaacgtttcggcgagaagcaggccattatcgcgggcatggcggccgacgcgctgggctacgtcttgct<br>ggcgttcgcgacgcgcggctggatggccttccccattatgattcttctcgcttccggcggcatcggtatgcccgcgttgcag<br>gccatgctgtcccgccaagtagatgacgaccatcagggacagcttcaagggtcgctcgcggctcttaccagcctcactt<br>cgatcattggaccgctgatcgtcacggcgatttatgccgcctcggcgagcacatggaacgggttggcatggattgtaggt<br>gccgccctttaccttgtctgcctccccgcgttgcgtcgcggtgcatggagccgggccacctcgacctaataatactagctc<br>cggcaaaaaaacgggcaaggtgtcaccaccctgcccttttctttaaaaccgaaaagattacttcgcgtt

Sequence name	*Dalbergia maritima* DmTrnL-UAA Toehold Switch 1.1 Trigger
Acc. number	BBa_K3453161
Sequence	tgccacctgacgtctaagaaaccattattatcatgacattaacctataaaaataggcgtatcacgaggcagaatttcagat<br>aaaaaaaatccttagctttcgctaaggatgatttctggaattcgcggccgcttctagagtaatacgactcactatagggtttc<br>aattgattaatgaagactcccaatttctatttgtgaatcgtcatctcacaattgaaagatgtgaatcaaatcaattccaagttg<br>aagaaagaattgaatattcactgatcaaatcatactcgaacccctagcccgctcttatcgggcggctaggggtttttgtta<br>ctagtagcggccgctgcagtccggcaaaaaagggcaaggtgtcaccaccctgccctttttctttaaaaccgaaaagatt<br>acttcgcgttatgcaggcttcctcgctcactgactcgctgcgctcggtcgttcggctgcggcgagcggtatcagctcactca<br>aaggcggtaatacggttatccacagaatcaggggataacgcaggaaagaacatgtgagcaaaaggccagcaaaa<br>ggccaggaaccgtaaaaaggccgcgttgctggcgtttttccacaggctccgcccccctgacgagcatcacaaaaatcg<br>acgctcaagtcagaggtggcgaaacccgacaggactataaagataccaggcgtttccccctggaagctccctcgtgc<br>gctctcctgttccgaccctgccgcttaccggatacctgtccgcctttctcccttcgggaagcgtggcgctttctcatagctcac<br>gctgtaggtatctcagttcggtgtaggtcgttcgctccaagctgggctgtgtgcacgaaccccccgttcagcccgaccgct<br>gcgccttatccggtaactatcgtcttgagtccaacccggtaagacacgacttatcgccactggcagcagccactggtaac<br>aggattagcagagcgaggtatgtaggcggtgctacagagttcttgaagtggtggcctaactacggctacactagaaga<br>acagtatttggtatctgcgctctgctgaagccagttaccttcggaaaaagagttggtagctcttgatccggcaaacaaacc<br>accgctggtagcggtggtttttttgtttgcaagcagcagattacgcgcagaaaaaaaggatctcaagaagatcctttgatct<br>tttctacggggtctgacgctcagtggaacgaaaactcacgttaagggattttggtcatgagattatcaaaaaggatcttcac<br>ctagatccttttaaattaaaaatgaagttttaaatcaatctaaagtatatatgagtaaacttggtctgacagctcgaggcttgg<br>attctcaccaataaaaaacgcccggcggcaaccgagcgttctgaacaaatccagatggagttctgaggtcattactgga<br>tctatcaacaggagtccaagcgagctcgatatcaaattacgccccgccctgccactcatcgcagtactgttgtaattcatta<br>agcattctgccgacatggaagccatcacaaacggcatgatgaacctgaatcgccagcggcatcagcaccttgtcgcctt<br>gcgtataatatttgcccatggtgaaaacgggggcgaagaagttgtccatattggccacgtttaaatcaaaactggtgaaa<br>ctcacccagggattggctgagacgaaaaacatattctcaataaaccctttagggaaataggccaggttttcaccgtaac<br>acgccacatcttgcgaatatatgtgtagaaactgccggaaatcgtcgtggtattcactccagagcgatgaaaacgtttca<br>gtttgctcatggaaaacggtgtaacaagggtgaacactatcccatatcaccagctcaccgtctttcattgccatacgaaatt<br>ccggatgagcattcatcaggcgggcaagaatgtgaataaaggccggataaaacttgtgcttatttttctttacggtctttaa<br>aaaggccgtaatatccagctgaacggtctggttataggtacattgagcaactgactgaaatgcctcaaaatgttctttacg<br>atgccattgggatatatcaacggtggtatatccagtgatttttttctccattttagcttccttagctcctgaaaatctcgataactc<br>aaaaaatacgcccggtagtgatcttatttcattatggtgaaagttggaacctcttacgtgcccgatcaactcgag

Sequence name	*Dalbergia maritima* DmTrnL-UAA Toehold Switch 1.2 Sensor
Acc. number	BBa_K3453152
Sequence	tgccacctgacgtctaagaaaaggaatattcagcaatttgcccgtgccgaagaaaggcccacccgtgaaggtgagcc<br>agtgagttgattgctacgtaattagttagttagcccttagtgactggaattcgcggccgcttctagagtaatacgactcactat<br>agggttcacaaatagaaattgggagtcttcattaatcaatatggactttagaacagaggagataaagatgatattgatga<br>atgaagacaacctggcggcagcgcaaaagatgcgtaaaggcgaagaactgtttaccggtgtggttccgattctggtgg<br>aactggacggcgatgttaatggtcataaattcagtgttcgcggcgaaggtgaaggcgatgcgacgaacggcaaactg<br>accctgaaatttatctgcaccacgggtaaactgccggtcccgtggccgacgctggtgaccacgctgacctatggcgttca<br>atgttttgcgcgttacccggatcacatgaaacagcacgactttttcaaatcggccatgccggaaggctatgtgcaggaac<br>gtacgattagctttaaagacgatggtacgtataaaacccgcgcggaagtgaaattcgaaggcgataccctggttaaccg<br>tatcgaactgaaaggtatcgatttcaaagaagatggcaatattctgggtcataaactggaatataacttcaattcccacaa<br>cgtgtacatcaccgcggataaacagaaaaacggcattaaagccaatttcaaaatccgccataatgtggaagatggtag<br>cgttcagctggccgaccactatcagcaaaacacgccgattggtgatggcccggtcctgctgccggacaatcactacctg<br>agtacccagtccgtgctgtcaaaagatccgaacgaaaaacgtgaccacatggtcctgctggaatttgtgacggctgcgg<br>gtatcacccacggcatggacgaactgtataaaggcctgctgcaaacgacgaaaactacgctttagtagcttaataata<br>ctcgaacccctagcccgctcttatcgggcggctaggggtttttgttactagtagcggccgctgcaggagtcactaagggtt<br>agttagttagattagcagaaagtcaaaagcctccgaccggaggcttttgactaaaacttcccttggggttatcattggggct<br>cactcaaaggcggtaatcagataaaaaaaatccttagctttcgctaaggatgatttctgctagagatggaatagactgga<br>tggaggcggataaagttgcaggaccacttctgcgctcggcccttccggctggctggtttattgctgataaatctggagccg<br>gtgagcgtgggactcgcggtatcattgcagcactggggccagatggtaagccctcccgtatcgtagttatctacacgacg<br>gggagtcaggcaactatggatgaacgaaatagacagatcgctgagataggtgcctcactgattaagcattggtaactgt<br>cagaccaagtttactcatatatactttagattgatttaaaacttcatttttaatttaaaaggatctaggtgaagatcctttttgata<br>atctcatgaccaaaatcccttaacgtgagttttcgttccactgagcgtcagaccccttaataagatgatcttcttgagatcgttt<br>tggtctgcgcgtaatctcttgctctgaaaacgaaaaaaccgccttgcagggcggtttttcgaaggttctctgagctaccaac<br>tctttgaaccgaggtaactggcttggaggagcgcagtcaccaaaacttgtcctttcagtttagccttaaccggcgcatgact<br>tcaagactaactcctctaaatcaattaccagtggctgctgccagtggtgcttttgcatgtctttccgggttggactcaagacg<br>atagttaccggataaggcgcagcggtcggactgaacggggggttcgtgcatacagtccagcttggagcgaactgccta

	cccggaactgagtgtcaggcgtggaatgagacaaacgcggccataacagcggaatgacaccggtaaaccgaaag<br>gcaggaacaggagagcgcacgagggagccgccaggggaaacgcctggtatctttatagtcctgtcgggtttcgccac<br>cactgatttgagcgtcagatttcgtgatgcttgtcaggggggcggagcctatggaaaaacggctttgccgcggccctctca<br>cttccctgttaagtatcttcctggcatcttccaggaaatctccgccccgttcgtaagccatttccgctcgccgcagtcgaacg<br>accgagcgtagcgagtcagtgagcgaggaagcggaatatatcctgtatcacatattctgctgacgcaccggtgcagcct<br>tttttctcctgccacatgaagcacttcactgacaccctcatcagtgccaacatagtaagccagtatacactccgctagcgct<br>gaggtctgcctcgtgaagaaggtgttgctgactcataccaggcctgaatcgccccatcatccagccagaaagtgaggg<br>agccacggttgatgagagctttgttgtaggtggaccagttggtgattttgaacttttgctttgccacggaacggtctgcgttgtc<br>gggaagatgcgtgatctgatccttcaactcagcaaaagttcgatttattcaacaaagccacgttgtgtctcaaaatctctga<br>tgttacattgcacaagataaaaatatatcatcatgaacaataaaactgtctgcttacataaacagtaatacaaggggtgttt<br>actagaggagattctcatgtttgacagcttatcatcgataagctttaatgcggtagtttatcacagttaaattgctaacgcagt<br>caggcaccgtgtatgaaatctaacaatgcgctcatcgtcattctcggcaccgtcaccctggacgctgtaggcataggctt<br>ggttatgccggtactgccgggcctcttgcgggatatcgtccattccgacagtattgccagtcactatggcgtgctgcttgcg<br>ctctatgcgttgatgcaatttctttgcgcacccgttctcggagccctgtccgaccgctttggccgccgtccagtcctgctcgctt<br>cgctccttggagccactatcgactacgcgatcatggcgaccacacccgtcctgtggattctctacgccggacgcatcgtg<br>gcgggcatcacgggtgccacaggtgcggttgctggtgcctatatcgccgacatcaccgatggggaagatcgggctcgc<br>cacttcgggctcatgagcgcttgtttcggcgtgggtatggtggcaggccccgtggccgggggactgttgggtgccatctcc<br>ttgcatgcaccattccttgcggcggcggtgctcaacggcctcaacctcctcctgggctgcttccttatgcaggaatcgcata<br>agggagagcgccgtccgatgcccttgcgtgccttcaatccagtcagctccttccggtgggcgcggggcatgactatcgtc<br>gccgcacttatgactgttttctttatcatgcaactcgtaggacaggttccggcagcgctctgggtcattttcggcgaggaccg<br>ctttcgctggagcgcgacgatgatcggcctgtcgcttgcggtattcggaatcttgcacgccctcgctcaagccttcgtcacg<br>ggccccgccaccaaacgtttcggcgagaagcaggccattatcgcgggcatggcggccgacgcgctgggctacgtctt<br>gctggcgttcgcgacgcgcggctggatggccttccccattatgattcttctcgcttccggcggcatcggtatgcccgcgttg<br>caggccatgctgtcccgccaagtagatgacgaccatcagggacagcttcaagggtcgctcgcggctcttaccagcctc<br>acttcgatcattggaccgctgatcgtcacggcgatttatgccgcctcggcgagcacatggaacgggttggcatggattgta<br>ggtgccgccctttaccttgtctgcctccccgcgttgcgtcgcggtgcatggagccgggccacctcgacctaataatactag<br>ctccggcaaaaaaacgggcaaggtgtcaccaccctgccctttttctttaaaaccgaaaagattacttcgcgtt

Sequence name	*Dalbergia maritima* DmTrnL-UAA Toehold Switch 1.2 Trigger
Acc. number	BBa_K3453162
Sequence	tgccacctgacgtctaagaaaccattattatcatgacattaacctataaaaataggcgtatcacgaggcagaatttcagat<br>aaaaaaaatccttagctttcgctaaggatgatttctggaattcgcggccgcttctagagtaatacgactcactatagggttg<br>attaatgaagactcccaatttctatttgtgaatcgtcatctcacaattgaaagatgtgaatcaaatcaattccaagttgaaga<br>aagaattgaatattcactgatcaaatcattcaattactcgaacccctagcccgctcttatcgggcggctaggggtttttttgtta<br>ctagtagcggccgctgcagtccggcaaaaaagggcaaggtgtcaccaccctgccctttttctttaaaaccgaaaagatt<br>acttcgcgttatgcaggcttcctcgctcactgactcgctgcgctcggtcgttcggctgcggcgagcggtatcagctcactca<br>aaggcggtaatacggttatccacagaatcaggggataacgcaggaaagaacatgtgagcaaaaggccagcaaaa<br>ggccaggaaccgtaaaaaggccgcgttgctggcgtttttccacaggctccgcccccctgacgagcatcacaaaaatcg<br>acgctcaagtcagaggtggcgaaacccgacaggactataaagataccaggcgtttccccctggaagctccctcgtgc<br>gctctcctgttccgaccctgccgcttaccggatacctgtccgcctttctcccttcgggaagcgtggcgctttctcatagctcac<br>gctgtaggtatctcagttcggtgtaggtcgttcgctccaagctgggctgtgtgcacgaaccccccgttcagcccgaccgct<br>gcgccttatccggtaactatcgtcttgagtccaacccggtaagacacgacttatcgccactggcagcagccactggtaac<br>aggattagcagagcgaggtatgtaggcggtgctacagagttcttgaagtggtggcctaactacggctacactagaaga<br>acagtatttggtatctgcgctctgctgaagccagttaccttcggaaaaagagttggtagctcttgatccggcaaacaaacc<br>accgctggtagcggtggtttttttgtttgcaagcagcagattacgcgcagaaaaaaaggatctcaagaagatcctttgatct<br>tttctacggggtctgacgctcagtggaacgaaaactcacgttaagggattttggtcatgagattatcaaaaaggatcttcac<br>ctagatccttttaaattaaaaatgaagttttaaatcaatctaaagtatatatgagtaaacttggtctgacagctcgaggcttgg<br>attctcaccaataaaaaacgcccggcggcaaccgagcgttctgaacaaatccagatggagttctgaggtcattactgga<br>tctatcaacaggagtccaagcgagctcgatatcaaattacgccccgccctgccactcatcgcagtactgttgtaattcatta<br>agcattctgccgacatggaagccatcacaaacggcatgatgaacctgaatcgccagcggcatcagcaccttgtcgcctt<br>gcgtataatatttgcccatggtgaaaacgggggcgaagaagttgtccatattggccacgtttaaatcaaaactggtgaaa<br>ctcacccagggattggctgagacgaaaaacatattctcaataaaccctttagggaaataggccaggttttcaccgtaac<br>acgccacatcttgcgaatatatgtgtagaaactgccggaaatcgtcgtggtattcactccagagcgatgaaaacgtttca<br>gtttgctcatggaaaacggtgtaacaagggtgaacactatcccatatcaccagctcaccgtctttcattgccatacgaaatt<br>ccggatgagcattcatcaggcgggcaagaatgtgaataaaggccggataaaacttgtgcttatttttctttacggtctttaa<br>aaaggccgtaatatccagctgaacggtctggttataggtacattgagcaactgactgaaatgcctcaaaatgttctttacg<br>atgccattgggatatatcaacggtggtatatccagtgatttttttctccattttagcttccttagctcctgaaaatctcgataactc<br>aaaaaatacgcccggtagtgatcttatttcattatggtgaaagttggaacctcttacgtgcccgatcaactcgag

Sequence name	*Dalbergia maritima* DmTrnL-UAA Toehold Switch 1.3 Sensor
Acc. number	BBa_K3453153
Sequence	tgccacctgacgtctaagaaaaggaatattcagcaatttgcccgtgccgaagaaaggcccacccgtgaaggtgagcc agtgagttgattgctacgtaattagttagttagcccttagtgactggaattcgcggccgcttctagagtaatacgactcactat agggtgatggagtgaattgaatgatttgatcagtgaatatatggactttagaacagaggagataaagatgatatatatgct gatcaaaaacctggcggcagcgcaaaagatgcgtaaaggcgaagaactgtttaccggtgtggttccgattctggtgga actggacggcgatgttaatggtcataaattcagtgttcgcggcgaaggtgaaggcgatgcgacgaacggcaaactgac cctgaaatttatctgcaccacgggtaaactgccggtcccgtggccgacgctggtgaccacgctgacctatggcgttcaat gttttgcgcgttacccggatcacatgaaacagcacgactttttcaaatcggccatgccggaaggctatgtgcaggaacgt acgattagctttaaagacgatggtacgtataaaacccgcgcggaagtgaaattcgaaggcgataccctggttaaccgta tcgaactgaaaggtatcgatttcaaagaagatggcaatattctgggtcataaactggaatataacttcaattcccacaacg tgtacatcaccgcggataaacagaaaaacggcattaaagccaatttcaaaatccgccataatgtggaagatggtagcg ttcagctggccgaccactatcagcaaaacacgccgattggtgatggcccggtcctgctgccggacaatcactacctgag tacccagtccgtgctgtcaaaagatccgaacgaaaaacgtgaccacatggtcctgctggaatttgtgacggctgcgggt atcacccacggcatggacgaactgtataaaggcctgctgcaaacgacgaaaactacgctttagtagcttaataatact cgaacccctagcccgctcttatcgggcggctaggggtttttttgttactagtagcggccgctgcaggagtcactaagggtta gttagttagattagcagaaagtcaaaagcctccgaccggaggcttttgactaaaacttcccttggggttatcattggggctc actcaaaggcggtaatcagataaaaaaaatccttagctttcgctaaggatgatttctgctagagatggaatagactggat ggaggcggataaagttgcaggaccacttctgcgctcggcccttccggctggctggtttattgctgataaatctggagccgg tgagcgtgggactcgcggtatcattgcagcactggggccagatggtaagccctcccgtatcgtagttatctacacgacgg ggagtcaggcaactatggatgaacgaaatagacagatcgctgagataggtgcctcactgattaagcattggtaactgtc agaccaagtttactcatatatactttagattgatttaaaacttcatttttaatttaaaaggatctaggtgaagatcctttttgataat ctcatgaccaaaatcccttaacgtgagttttcgttccactgagcgtcagaccccttaataagatgatcttcttgagatcgttttg gtctgcgcgtaatctcttgctctgaaaacgaaaaaaccgccttgcagggcggtttttcgaaggttctctgagctaccaactc tttgaaccgaggtaactggcttggaggagcgcagtcaccaaaacttgtcctttcagtttagccttaaccggcgcatgacttc aagactaactcctctaaatcaattaccagtggctgctgccagtggtgcttttgcatgtctttccgggttggactcaagacgat agttaccggataaggcgcagcggtcggactgaacggggggttcgtgcatacagtccagcttggagcgaactgcctacc cggaactgagtgtcaggcgtggaatgagacaaacgcggccataacagcggaatgacaccggtaaaccgaaaggc aggaacaggagagcgcacgagggagccgccaggggaaacgcctggtatctttatagtcctgtcgggtttcgccacca ctgatttgagcgtcagatttcgtgatgcttgtcaggggggcggagcctatggaaaaacggctttgccgcggccctctcactt ccctgttaagtatcttcctggcatcttccaggaaatctccgccccgttcgtaagccatttccgctcgccgcagtcgaacgac cgagcgtagcgagtcagtgagcgaggaagcggaatatatcctgtatcacatattctgctgacgcaccggtgcagcctttt ttctcctgccacatgaagcacttcactgacaccctcatcagtgccaacatagtaagccagtatacactccgctagcgctg aggtctgcctcgtgaagaaggtgttgctgactcataccaggcctgaatcgccccatcatccagccagaaagtgaggga gccacggttgatgagagctttgttgtaggtggaccagttggtgattttgaacttttgctttgccacggaacggtctgcgttgtcg ggaagatgcgtgatctgatccttcaactcagcaaaagttcgatttattcaacaaagccacgttgtgtctcaaaatctctgat gttacattgcacaagataaaaatatatcatcatgaacaataaaactgtctgcttacataaacagtaatacaaggggtgttt actagaggagattctcatgtttgacagcttatcatcgataagctttaatgcggtagtttatcacagttaaattgctaacgcagt caggcaccgtgtatgaaatctaacaatgcgctcatcgtcattctcggcaccgtcaccctggacgctgtaggcataggctt ggttatgccggtactgccgggcctcttgcgggatatcgtccattccgacagtattgccagtcactatggcgtgctgcttgcg ctctatgcgttgatgcaatttctttgcgcacccgttctcggagccctgtccgaccgctttggccgccgtccagtcctgctcgctt cgctccttggagccactatcgactacgcgatcatggcgaccacacccgtcctgtggattctctacgccggacgcatcgtg gcgggcatcacgggtgccacaggtgcggttgctggtgcctatatcgccgacatcaccgatggggaagatcgggctcgc cacttcgggctcatgagcgcttgtttcggcgtgggtatggtggcaggccccgtggccgggggactgttgggtgccatctcc ttgcatgcaccattccttgcggcggcggtgctcaacggcctcaacctcctcctgggctgcttcctaatgcaggaatcgcata agggagagcgccgtccgatgcccttgcgtgccttcaatccagtcagctccttccggtgggcgcggggcatgactatcgtc gccgcacttatgactgttttctttatcatgcaactcgtaggacaggttccggcagcgctctgggtcattttcggcgaggaccg ctttcgctggagcgcgacgatgatcggcctgtcgcttgcggtattcggaatcttgcacgccctcgctcaagccttcgtcacg ggccccgccaccaaacgtttcggcgagaagcaggccattatcgcgggcatggcggccgacgcgctgggctacgtctt gctggcgttcgcgacgcgcggctggatggccttccccattatgattcttctcgcttccggcggcatcggtatgcccgcgttg caggccatgctgtcccgccaagtagatgacgaccatcagggacagcttcaagggtcgctcgcggctcttaccagcctc acttcgatcattggaccgctgatcgtcacggcgatttatgccgcctcggcgagcacatggaacgggttggcatggattgta ggtgccgccctttaccttgtctgcctccccgcgttgcgtcgcggtgcatggagccgggccacctcgacctaataatactag ctccggcaaaaaaacgggcaaggtgtcaccaccctgccctttttctttaaaaccgaaaagattacttcgcgtt

Sequence name	*Dalbergia maritima* DmTrnL-UAA Toehold Switch 1.3 Trigger
Acc. number	BBa_K3453163
Sequence	tgccacctgacgtctaagaaaccattattatcatgacattaacctataaaaataggcgtatcacgaggcagaatttcagat aaaaaaaatccttagctttcgctaaggatgatttctggaattcgcggccgcttctagagtaatacgactcactatagggtatt cactgatcaaatcattcaattcactccatcatagtctgatagatcttttgaagaactgattaatcggacgagaataaagaga gagtcccattctacatgtcaatactgacaacaattactcgaacccctagcccgctcttatcgggcggctaggggtttttgtt actagtagcggccgctgcagtccggcaaaaaagggcaaggtgtcaccaccctgccctttttctttaaaaccgaaaagat tacttcgcgttatgcaggcttcctcgctcactgactcgctgcgctcggtcgttcggctgcggcgagcggtatcagctcactc aaaggcggtaatacggttatccacagaatcaggggataacgcaggaaagaacatgtgagcaaaaggccagcaaa aggccaggaaccgtaaaaaggccgcgttgctggcgtttttccacaggctccgcccccctgacgagcatcacaaaaatc gacgctcaagtcagaggtggcgaaacccgacaggactataaagataccaggcgtttccccctggaagctccctcgtg cgctctcctgttccgaccctgccgcttaccggatacctgtccgcctttctcccttcgggaagcgtggcgctttctcatagctca cgctgtaggtatctcagttcggtgtaggtcgttcgctccaagctgggctgtgtgcacgaaccccccgttcagcccgaccgc tgcgccttatccggtaactatcgtcttgagtccaacccggtaagacacgacttatcgccactggcagcagccactggtaa caggattagcagagcgaggtatgtaggcggtgctacagagttcttgaagtggtggcctaactacggctacactagaag aacagtatttggtatctgcgctctgctgaagccagttaccttcggaaaaagagttggtagctcttgatccggcaaacaaac caccgctggtagcggtggtttttttgtttgcaagcagcagattacgcgcagaaaaaaaggatctcaagaagatcctttgat cttttctacggggtctgacgctcagtggaacgaaaactcacgttaagggattttggtcatgagattatcaaaaaggatcttc acctagatccttttaaattaaaaatgaagttttaaatcaatctaaagtatatatgagtaaacttggtctgacagctcgaggctt ggattctcaccaataaaaaacgcccggcggcaaccgagcgttctgaacaaatccagatggagttctgaggtcattactg gatctatcaacaggagtccaagcgagctcgatatcaaattacgccccgccctgccactcatcgcagtactgttgtaattca ttaagcattctgccgacatggaagccatcacaaacggcatgatgaacctgaatcgccagcggcatcagcaccttgtcg ccttgcgtataatatttgcccatggtgaaaacgggggcgaagaagttgtccatattggccacgtttaaatcaaaactggtg aaactcacccagggattggctgagacgaaaaacatattctcaataaaccctttagggaaataggccaggttttcaccgt aacacgccacatcttgcgaatatatgtgtagaaactgccggaaatcgtcgtggtattcactccagagcgatgaaaacgtt tcagtttgctcatggaaaacggtgtaacaagggtgaacactatcccatatcaccagctcaccgtctttcattgccatacga aattccggatgagcattcatcaggcgggcaagaatgtgaataaaggccggataaaacttgtgcttatttttctttacggtctt taaaaaggccgtaatatccagctgaacggtctggttataggtacattgagcaactgactgaaatgcctcaaaatgttcttta cgatgccattgggatatatcaacggtggtatatccagtgatttttttctccattttagcttccttagctcctgaaaatctcgataa ctcaaaaaatacgcccggtagtgatcttatttcattatggtgaaagttggaacctcttacgtgcccgatcaactcgag

Sequence name	*Dalbergia maritima* DmMatK Trigger (Full)
Acc. number	BBa_K3453120
Sequence	tgccacctgacgtctaagaaaccattattatcatgacattaacctataaaaataggcgtatcacgaggcagaatttcagat aaaaaaaatccttagctttcgctaaggatgatttctggaattcgcggccgcttctagagtaatacgactcactataggggg aatagtcttattactaaaaaaaaacttatttctactttttcaaaaagtaatccaagaattctcttgttcctatataatttttatgtatg tgaacacgaatccatcttcctttttctacgtaagagatcctcttatttacgattaaactcttttatcgttatttttgagcgaatctattt ctatgcaaaaatcgaacatcttgtggaagtcttttctaagaatttttcgtctaccttatcattcttcaaggatcctttgattcattac gttagatatcaaggaaaagccattctggcttcaaagaatgcgcctcttttgatgaataaatggaaacactatctcatctattt ctggcaatgtcattttgatgtttggtctcaacctggaacgatccatataaatccattattatccgagcattcatttcactttttttgg gggggctatctttcaaatgtgcggctcaatttttcagtggtccggaatcaaatgctagaaaattcatttctaatcgaaatttta tgaaaaagcttgatacaatagttccaattattccttttactcgaacccctagcccgctcttatcgggcggctaggggtttttgtt actagtagcggccgctgcagtccggcaaaaaagggcaaggtgtcaccaccctgccctttttctttaaaaccgaaaagat tacttcgcgttatgcaggcttcctcgctcactgactcgctgcgctcggtcgttcggctgcggcgagcggtatcagctcactc aaaggcggtaatacggttatccacagaatcaggggataacgcaggaaagaacatgtgagcaaaaggccagcaaa aggccaggaaccgtaaaaaggccgcgttgctggcgtttttccacaggctccgcccccctgacgagcatcacaaaaatc gacgctcaagtcagaggtggcgaaacccgacaggactataaagataccaggcgtttccccctggaagctccctcgtg cgctctcctgttccgaccctgccgcttaccggatacctgtccgcctttctcccttcgggaagcgtggcgctttctcatagctca cgctgtaggtatctcagttcggtgtaggtcgttcgctccaagctgggctgtgtgcacgaaccccccgttcagcccgaccgc tgcgccttatccggtaactatcgtcttgagtccaacccggtaagacacgacttatcgccactggcagcagccactggtaa caggattagcagagcgaggtatgtaggcggtgctacagagttcttgaagtggtggcctaactacggctacactagaag aacagtatttggtatctgcgctctgctgaagccagttaccttcggaaaaagagttggtagctcttgatccggcaaacaaac caccgctggtagcggtggtttttttgtttgcaagcagcagattacgcgcagaaaaaaaggatctcaagaagatcctttgat cttttctacggggtctgacgctcagtggaacgaaaactcacgttaagggattttggtcatgagattatcaaaaaggatcttc acctagatccttttaaattaaaaatgaagttttaaatcaatctaaagtatatatgagtaaacttggtctgacagctcgaggctt ggattctcaccaataaaaaacgcccggcggcaaccgagcgttctgaacaaatccagatggagttctgaggtcattactg gatctatcaacaggagtccaagcgagctcgatatcaaattacgccccgccctgccactcatcgcagtactgttgtaattca ttaagcattctgccgacatggaagccatcacaaacggcatgatgaacctgaatcgccagcggcatcagcaccttgtcg

	ccttgcgtataatatttgcccatggtgaaaacgggggcgaagaagttgtccatattggccacgtttaaatcaaaactggtg<br>aaactcacccagggattggctgacacgaaaaacatattctcaataaaccctttagggaaataggccaggttttcaccgt<br>aacacgccacatcttgcgaatatatgtgtagaaactgccggaaatcgtcgtggtattcactccagagcgatgaaaacgtt<br>tcagtttgctcatggaaaacggtgtaacaagggtgaacactatcccatatcaccagctcaccgtctttcattgccatacga<br>aattccggatgagcattcatcaggcgggcaagaatgtgaataaaggccggataaaacttgtgcttatttttctttacggtctt<br>taaaaaggccgtaatatccagctgaacggtctggttataggtacattgagcaactgactgaaatgcctcaaaatgttcttta<br>cgatgccattgggatatatcaacggtggtatatccagtgatttttttctccattttagcttccttagctcctgaaaatctcgataa<br>ctcaaaaaatacgcccggtagtgatcttatttcattatggtgaaagttggaacctcttacgtgcccgatcaactcgag

Sequence name	*Dalbergia maritima* DmRbcL Trigger (Full)
Acc. number	BBa_K3453140
Sequence	tgccacctgacgtctaagaaaccattattatcatgacattaacctataaaaataggcgtatcacgaggcagaatttcagat<br>aaaaaaaatccttagctttcgctaaggatgatttctggaattcgcggccgcttctagagtaatacgactcactatagggaat<br>tgacttattatactcctgactatgaaacgaaagatactgatatcttggccgcattccgagtaactcctcaacctggagttcct<br>cctgaagaagcgggtgccgcggtagctgccgaatcttctactggtacatggacaaccgtttggaccgatgggcttacca<br>gtcttgatcgttacaaaggacgatgctacaacatcgagcccgttgctggagaagagaatcaatatattgcttatgtagctta<br>tcccttagaccttttttgaagaaggttctgttactaacatgtttacttccattgtaggtaatgtatttgggttcaaggccctgcgcg<br>ccctacgtctggaagatttgcgaatccctacttcttatattaaaactttccaaggtccgcctcacggtatccaagttgaaaga<br>gataaattaaacaagtatggccgtccactattgggatgtactattaaaccgaaattggggttatccgctaagaattacggt<br>agagcagtttatgtactcgaacccctagcccgctcttatcgggcggctaggggtttttgttactagtagcggccgctgcagt<br>ccggcaaaaaagggcaaggtgtcaccaccctgccctttttctttaaaaccgaaaagattacttcgcgttatgcaggcttcc<br>tcgctcactgactcgctgcgctcggtcgttcggctgcggcgagcggtatcagctcactcaaaggcggtaatacggttatcc<br>acagaatcaggggataacgcaggaaagaacatgtgagcaaaaggccagcaaaaggccaggaaccgtaaaaag<br>gccgcgttgctggcgtttttccacaggctccgcccccctgacgagcatcacaaaaatcgacgctcaagtcagaggtggc<br>gaaacccgacaggactataaagataccaggcgtttccccctggaagctccctcgtgcgctctcctgttccgaccctgccg<br>cttaccggatacctgtccgcctttctcccttcgggaagcgtggcgctttctcatagctcacgctgtaggtatctcagttcggtgt<br>aggtcgttcgctccaagctgggctgtgtgcacgaaccccccgttcagcccgaccgctgcgccttatccggtaactatcgtc<br>ttgagtccaacccggtaagacacgacttatcgccactggcagcagccactggtaacaggattagcagagcgaggtatg<br>taggcggtgctacagagttcttgaagtggtggcctaactacggctacactagaagaacagtatttggtatctgcgctctgct<br>gaagccagttaccttcggaaaaagagttggtagctcttgatccggcaaacaaaccaccgctggtagcggtggtttttttgtt<br>tgcaagcagcagattacgcgcagaaaaaaaggatctcaagaagatcctttgatcttttctacggggtctgacgctcagtg<br>gaacgaaaactcacgttaagggattttggtcatgagattatcaaaaaggatcttcacctagatccttttaaattaaaaatga<br>agttttaaatcaatctaaagtatatatgagtaaacttggtctgacagctcgaggcttggattctcaccaataaaaaacgccc<br>ggcggcaaccgagcgttctgaacaaatccagatggagttctgaggtcattactggatctatcaacaggagtccaagcg<br>agctcgatatcaaattacgccccgccctgccactcatcgcagtactgttgtaattcattaagcattctgccgacatggaagc<br>catcacaaacggcatgatgaacctgaatcgccagcggcatcagcaccttgtcgccttgcgtataatatttgcccatggtg<br>aaaacgggggcgaagaagttgtccatattggccacgtttaaatcaaaactggtgaaactcacccagggattggctgag<br>acgaaaaacatattctcaataaaccctttagggaaataggccaggttttcaccgtaacacgccacatcttgcgaatatat<br>gtgtagaaactgccggaaatcgtcgtggtattcactccagagcgatgaaaacgtttcagtttgctcatggaaaacggtgta<br>acaagggtgaacactatcccatatcaccagctcaccgtctttcattgccatacgaaattccggatgagcattcatcaggc<br>gggcaagaatgtgaataaaggccggataaaacttgtgcttatttttctttacggtctttaaaaaggccgtaatatccagctg<br>aacggtctggttataggtacattgagcaactgactgaaatgcctcaaaatgttctttacgatgccattgggatatatcaacg<br>gtggtatatccagtgatttttttctccattttagcttccttagctcctgaaaatctcgataactcaaaaaatacgcccggtagtg<br>atcttatttcattatggtgaaagttggaacctcttacgtgcccgatcaactcgag

Sequence name	*Dalbergia maritima* DmTrnL-UAA Trigger (Full)
Acc. number	BBa_K3453160
Sequence	tgccacctgacgtctaagaaaccattattatcatgacattaacctataaaaataggcgtatcacgaggcagaatttcagat<br>aaaaaaaatccttagctttcgctaaggatgatttctggaattcgcggccgcttctagagtaatacgactcactatagggag<br>aaaccccggaattaacaatgggcaatcctgagccaaatcccgttttctgaaagcaaagaaaagttaagaaagcgaga<br>ataaaaaaaaggataggtgcagagactcaatggaagctgttctaacaaatggggttgacgatatttcctttcgtgttagga<br>aaagaatccttccatcgaaattataaaaaggtacgtatttgtactgaaatactatttcaattgattaatgaagactcccaattt<br>ctatttgtgaatcgtcatctcacaattgaaagatgtgaatcaaatcaattccaagttgaagaaagaattgaatattcactgat<br>caaatcattcaattcactccatcatagtctgatagatcttttgaagaactgattaatcggacgagaataaagagagagtcc<br>cattctacatgtcaatactgacaacaatgaaatttatactcgaacccctagcccgctcttatcgggcggctaggggtttttgt<br>tactagtagcggccgctgcagtccggcaaaaaagggcaaggtgtcaccaccctgccctttttctttaaaaccgaaaaga<br>ttacttcgcgttatgcaggcttcctcgctcactgactcgctgcgctcggtcgttcggctgcggcgagcggtatcagctcactc<br>aaaggcggtaatacggttatccacagaatcaggggataacgcaggaaagaacatgtgagcaaaaggccagcaaa

	aggccaggaaccgtaaaaaggccgcgttgctggcgtttttccacaggctccgcccccctgacgagcatcacaaaaatc<br>gacgctcaagtcagaggtggcgaaacccgacaggactataaagataccaggcgtttccccctggaagctccctcgtg<br>cgctctcctgttccgaccctgccgcttaccggatacctgtccgcctttctcccttcgggaagcgtggcgctttctcatagctca<br>cgctgtaggtatctcagttcggtgtaggtcgttcgctccaagctgggctgtgtgcacgaaccccccgttcagcccgaccgc<br>tgcgccttatccggtaactatcgtcttgagtccaacccggtaagacacgacttatcgccactggcagcagccactggtaa<br>caggattagcagagcgaggtatgtaggcggtgctacagagttcttgaagtggtggcctaactacggctacactagaag<br>aacagtatttggtatctgcgctctgctgaagccagttaccttcggaaaaagagttggtagctcttgatccggcaaacaaac<br>caccgctggtagcggtggtttttttgtttgcaagcagcagattacgcgcagaaaaaaaggatctcaagaagatcctttgat<br>cttttctacggggtctgacgctcagtggaacgaaaactcacgttaagggattttggtcatgagattatcaaaaaggatcttc<br>acctagatccttttaaattaaaaatgaagttttaaatcaatctaaagtatatatgagtaaacttggtctgacagctcgaggctt<br>ggattctcaccaataaaaaacgcccggcggcaaccgagcgttctgaacaaatccagatggagttctgaggtcattactg<br>gatctatcaacaggagtccaagcgagctcgatatcaaattacgccccgccctgccactcatcgcagtactgttgtaattca<br>ttaagcattctgccgacatggaagccatcacaaacggcatgatgaacctgaatcgccagcggcatcagcaccttgtcg<br>ccttgcgtataatatttgcccatggtgaaaacgggggcgaagaagttgtccatattggccacgtttaaatcaaaactggtg<br>aaactcacccagggattggctgagacgaaaaacatattctcaataaaccctttagggaaataggccaggttttcaccgt<br>aacacgccacatcttgcgaatatatgtgtagaaactgccggaaatcgtcgtggtattcactccagagcgatgaaaacgtt<br>tcagtttgctcatggaaaacggtgtaacaagggtgaacactatcccatatcaccagctcaccgtctttcattgccatacga<br>aattccggatgagcattcatcaggcgggcaagaatgtgaataaaggccggataaaacttgtgcttatttttctttacggtctt<br>taaaaaggccgtaatatccagctgaacggtctggttataggtacattgagcaactgactgaaatgcctcaaaatgttcttta<br>cgatgccattgggatatatcaacggtggtatatccagtgatttttttctccattttagcttccttagctcctgaaaatctcgataa<br>ctcaaaaaatacgcccggtagtgatcttatttcattatggtgaaagttggaacctcttacgtgcccgatcaactcgag

Sequence name	*Acacia melanoxylon* AmMatK Trigger 1.1
Acc. number	BBa_K3453320
Sequence	ctgccacctgacgtctaagaaagcttggtctcatggaattcgcggccgcttctagagtaatacgactcactatagggcaa<br>ccaggaaagattcatataaaccaattatccgagcattcattttactttttgggttatttttcaaatgtgcggctaaatccttcagt<br>ggtacggagtcaaatgttggaaaagtcattttactcgaacccctagcccgctcttatcgggcggctaggggtttttttgttact<br>agtagcggccgctgcagtgagaccggatccgctcactcaaaggcggtaatc

Sequence name	*Berchemia zeyheri* BzMatK Trigger 1.1
Acc. number	BBa_K3453321
Sequence	ctgccacctgacgtctaagaaagcttggtctcatggaattcgcggccgcttctagagtaatacgactcactatagggcaa<br>cccggaaggatctacataaaccaattattcaagcattccttcggctttttgggctatctttcaagtgtgcgactaaatctttcatt<br>ggtacggagtcaaacgctcgaaaattcattttactcgaacccctagcccgctcttatcgggcggctaggggtttttttgttact<br>agtagcggccgctgcagtgagaccggatccgctcactcaaaggcggtaatc

Sequence name	*Dalbergia cochinchinensis* DcMatK Trigger 1.1
Acc. number	BBa_K3453322
Sequence	ctgccacctgacgtctaagaaagcttggtctcatggaattcgcggccgcttctagagtaatacgactcactatagggcaa<br>cctggaacgatccattatataactccactattatccgagcattcatttcactttttttgggggggctatctttcaaatgtgcggct<br>caatttttcagtggtccggaatcaaatgctatactcgaacccctagcccgctcttatcgggcggctaggggtttttttgttacta<br>gtagcggccgctgcagtgagaccggatccgctcactcaaaggcggtaatc

Sequence name	*Dalbergia hupeana* DhMatK Trigger 1.1
Acc. number	BBa_K3453323
Sequence	ctgccacctgacgtctaagaaagcttggtctcatggaattcgcggccgcttctagagtaatacgactcactatagggcaa<br>cccggaacgatccatataaatccgttattatccgagcattcatttcactttttttgggggggctatctttcaaatgtgcggctca<br>atttttcagcggtccggaatcaaatgctagaatactcgaacccctagcccgctcttatcgggcggctaggggtttttttgttact<br>agtagcggccgctgcagtgagaccggatccgctcactcaaaggcggtaatc

Sequence name	*Dalbergia ovata* DoMatK Trigger 1.1
Acc. number	BBa_K3453324
Sequence	ctgccacctgacgtctaagaaagcttggtctcatggaattcgcggccgcttctagagtaatacgactcactatagggcaa<br>cctggaacgatccatcatataaatccactattatccgagcattcatttcactttttttgggggggctatctttcaaatgtgcggct

	caatttttcagtggtccggaatcaaatgctatactcgaacccctagcccgctcttatcgggcggctaggggtttttgttacta<br>gtagcggccgctgcagtgagaccggatccgctcactcaaaggcggtaatc

Sequence name	*Dalbergia pervillei* DpMatK Trigger 1.1
Acc. number	BBa_K3453325
Sequence	ctgccacctgacgtctaagaaagcttggtctcatggaattcgcggccgcttctagagtaatacgactcactatagggcaa<br>cctgaaacgatccatataaatccattattatccgagcattcatttcactttttttgggggggctatctttcaaatgtgcggctca<br>atttttcagtggtccggaatcaaatgctagaatactcgaacccctagcccgctcttatcgggcggctaggggtttttgttact<br>agtagcggccgctgcagtgagaccggatccgctcactcaaaggcggtaatc

Sequence name	*Erythroxylum havanense* EhMatK Trigger 1.1
Acc. number	BBa_K3453326
Sequence	ctgccacctgacgtctaagaaagcttggtctcatggaattcgcggccgcttctagagtaatacgactcactatagggcag<br>ccggaaaagatcgatataaactcattatccaaacactctttagactttttgggctatttttcaagtgtacgacaaaatccttcg<br>gcgatacgtagtcaaatgatagaaaaatcattttactcgaacccctagcccgctcttatcgggcggctaggggtttttgtta<br>ctagtagcggccgctgcagtgagaccggatccgctcactcaaaggcggtaatc

Sequence name	*Millettia laurentii* MlMatK Trigger 1.1
Acc. number	BBa_K3453327
Sequence	ctgccacctgacgtctaagaaagcttggtctcatggaattcgcggccgcttctagagtaatacgactcactataggggaa<br>ccaggaacgatctatataaatcaattatccgagcattcatttcgctttttgggctatttttaaatgtgcagttaaatctttcagtg<br>gtacggagtcaaatgttacaaaattcattttactcgaacccctagcccgctcttatcgggcggctaggggtttttgttactag<br>tagcggccgctgcagtgagaccggatccgctcactcaaaggcggtaatc

Sequence name	*Machaerium scleroxylon* MsMatK Trigger 1.1
Acc. number	BBa_K3453328
Sequence	ctgccacctgacgtctaagaaagcttggtctcatggaattcgcggccgcttctagagtaatacgactcactatagggcaa<br>cctgaaacgatccatataaacccattattatccgagcattcatttcatttttttgggggggctatctttcaaatgtgcgactac<br>atttttcagtggtacggaatcaaatgctagaatactcgaacccctagcccgctcttatcgggcggctaggggtttttgttact<br>agtagcggccgctgcagtgagaccggatccgctcactcaaaggcggtaatc

Sequence name	*Pterocarpus indicus* PiMatK Trigger 1.1
Acc. number	BBa_K3453329
Sequence	ctgccacctgacgtctaagaaagcttggtctcatggaattcgcggccgcttctagagtaatacgactcactatagggcaa<br>ccaggaacgatccatataaaccaattattatccgagcattcatttaactttttttgggggggttatctttcaaatgtgcggctaa<br>atttttcagtggtacggagtcaaatgctagaatactcgaacccctagcccgctcttatcgggcggctaggggtttttgttact<br>agtagcggccgctgcagtgagaccggatccgctcactcaaaggcggtaatc

Sequence name	*Acacia melanoxylon* AmRbcL Trigger 1.1
Acc. number	BBa_K3453340
Sequence	ctgccacctgacgtctaagaaagcttggtctcatggaattcgcggccgcttctagagtaatacgactcactatagggatttg<br>cgaatccctccttcttattctaaaactttccaaggtccgcctcacggcatccaagttgagagagataaattgaacaagtac<br>ggccgtcccctattgggatgtactattaaaccaatactcgaacccctagcccgctcttatcgggcggctaggggtttttgtt<br>actagtagcggccgctgcagtgagaccggatccgctcactcaaaggcggtaatc

Sequence name	*Berchemia zeyheri* BzRbcL Trigger 1.1
Acc. number	BBa_K3453341
Sequence	ctgccacctgacgtctaagaaagcttggtctcatggaattcgcggccgcttctagagtaatacgactcactatagggatttg<br>cgaatccccccctgcttattctaaaactttccaaggcccgcctcatggcatccaagttgagagagataagttgaacaagtat<br>ggccgccccctattgggatgtactattaaaccgatactcgaacccctagcccgctcttatcgggcggctaggggtttttgtt<br>actagtagcggccgctgcagtgagaccggatccgctcactcaaaggcggtaatc

Sequence name	*Dalbergia baronii* DbRbcL Trigger 1.1
Acc. number	BBa_K3453342
Sequence	ctgccacctgacgtctaagaaagcttggtctcatggaattcgcggccgcttctagagtaatacgactcactatagggatttg<br>cgaatccctacttcttatgttaaaactttccaaggtccgcctcacggtatccaagttgaaagagataaattaaacaagtatg<br>gccgtcccctattgggatgtactattaaaccgatactcgaacccctagcccgctcttatcgggcggctaggggtttttgtta<br>ctagtagcggccgctgcagtgagaccggatccgctcactcaaaggcggtaatc

Sequence name	*Dysoxylum fraserianum* DfRbcL Trigger 1.1
Acc. number	BBa_K3453343
Sequence	ctgccacctgacgtctaagaaagcttggtctcatggaattcgcggccgcttctagagtaatacgactcactatagggatct<br>acgaatccctaccgcatatattaaaactttccaaggtccacctcatggcatccaagttgagagagataaattgaacaagt<br>atggccgtcccttattgggatgtactattaaaccaatactcgaacccctagcccgctcttatcgggcggctaggggtttttgt<br>tactagtagcggccgctgcagtgagaccggatccgctcactcaaaggcggtaatc

Sequence name	*Dalbergia granadillo* DgRbcL Trigger 1.1
Acc. number	BBa_K3453344
Sequence	ctgccacctgacgtctaagaaagcttggtctcatggaattcgcggccgcttctagagtaatacgactcactatagggatttg<br>cgaatccctacttcttatattaaaacttttcaaggtccgcctcacggtatccaagttgaaagagataaattaaacaagtatg<br>gccgtcccctattgggatgtactattaaaccgatactcgaacccctagcccgctcttatcgggcggctaggggtttttgtta<br>ctagtagcggccgctgcagtgagaccggatccgctcactcaaaggcggtaatc

Sequence name	*Dalbergia pervillei* DpRbcL Trigger 1.1
Acc. number	BBa_K3453345
Sequence	ctgccacctgacgtctaagaaagcttggtctcatggaattcgcggccgcttctagagtaatacgactcactatagggatttg<br>cgaatacctacttcttatattaaaactttccaaggtccgcctcacggtatccaagttgaaagagataaattaaacaagtatg<br>gccgtcccctattgggatgtactattaaaccgatactcgaacccctagcccgctcttatcgggcggctaggggtttttgtta<br>ctagtagcggccgctgcagtgagaccggatccgctcactcaaaggcggtaatc

Sequence name	*Erythroxylum havanense* EhRbcL Trigger 1.1
Acc. number	BBa_K3453346
Sequence	ctgccacctgacgtctaagaaagcttggtctcatggaattcgcggccgcttctagagtaatacgactcactatagggatttg<br>cgaattcctaaagcttatactaaaactttccaaggcccgcctcatggcatccaatctgagagagataaattgaacaagta<br>cggccgcccccctattgggctgtactattaaacctatactcgaacccctagcccgctcttatcgggcggctaggggtttttgtt<br>actagtagcggccgctgcagtgagaccggatccgctcactcaaaggcggtaatc

Sequence name	*Metopium brownei* MbRbcL Trigger 1.1
Acc. number	BBa_K3453347
Sequence	ctgccacctgacgtctaagaaagcttggtctcatggaattcgcggccgcttctagagtaatacgactcactatagggatct<br>acgaatccctaccgcgtatacaaaaactttccaaggaccaccgcatgggatccaagttgagagagataaattgaaca<br>agtatggacgtcccctattgggatgtactattaaacctatactcgaacccctagcccgctcttatcgggcggctaggggtttt<br>ttgttactagtagcggccgctgcagtgagaccggatccgctcactcaaaggcggtaatc

Sequence name	*Millettia laurentii* MIRbcL Trigger 1.1
Acc. number	BBa_K3453348
Sequence	ctgccacctgacgtctaagaaagcttggtctcatggaattcgcggccgcttctagagtaatacgactcactatagggatttg<br>cgaatccctaattcttatattaaaactttccaaggtccacctcatggtatccaagttgagagagataaattgaacaagtatg<br>gacgtcccctattgggatgtactattaaacctatactcgaacccctagcccgctcttatcgggcggctaggggtttttgttac<br>tagtagcggccgctgcagtgagaccggatccgctcactcaaaggcggtaatc

Sequence name	*Machaerium scleroxylon* MsRbcL Trigger 1.1
Acc. number	BBa_K3453349
Sequence	ctgccacctgacgtctaagaaagcttggtctcatggaattcgcggccgcttctagagtaatacgactcactatagggatttg<br>cgaattcctacttcttatattaaaactttccagggtccgcctcacggtatccaagttgaaagagataaattaaacaagtatg<br>gccgtcccctattgggatgtactattaaaccgatactcgaacccctagcccgctcttatcgggcggctaggggtttttgtta<br>ctagtagcggccgctgcagtgagaccggatccgctcactcaaaggcggtaatc

Sequence name	*Acacia excelsa* AeTrnL-UAA Trigger 1.3
Acc. number	BBa_K3453360
Sequence	ctgccacctgacgtctaagaaagcttggtctcatggaattcgcggccgcttctagagtaatacgactcactatagggtatta<br>attgatcaaattcattccatgatagtctgatagatttttgaagaacttattaatcagacgagaataaagatagagtcccattc<br>tacatgtcaataccgacaataatgaaatttatactcgaacccctagcccgctcttatcgggcggctaggggtttttgttacta<br>gtagcggccgctgcagtgagaccggatccgctcactcaaaggcggtaatc

Sequence name	*Acacia melanoxylon* AmTrnL-UAA Trigger 1.3
Acc. number	BBa_K3453361
Sequence	ctgccacctgacgtctaagaaagcttggtctcatggaattcgcggccgcttctagagtaatacgactcactatagggtatta<br>attgatcaaattcactccatgatagtctgatagatttttgaagaactgattaatcagacgagaataaagatagagtcccatt<br>ctacatgtcaataccgacaataatgaaatttatactcgaacccctagcccgctcttatcgggcggctaggggtttttgttact<br>agtagcggccgctgcagtgagaccggatccgctcactcaaaggcggtaatc

Sequence name	*Berchemia zeyheri* BzTrnL-UAA Trigger 1.3
Acc. number	BBa_K3453362
Sequence	ctgccacctgacgtctaagaaagcttggtctcatggaattcgcggccgcttctagagtaatacgactcactatagggtattc<br>attgatcaaatcatttactccatcgtaatctgatagatcttttgaagaattgattaatcggacgagaataaagatagagtccc<br>gttctacatgtcaatatcgacaacaatgcaattactcgaacccctagcccgctcttatcgggcggctaggggtttttgttact<br>agtagcggccgctgcagtgagaccggatccgctcactcaaaggcggtaatc

Sequence name	*Dalbergia baronii* DbTrnL-UAA Trigger 1.3
Acc. number	BBa_K3453363
Sequence	ctgccacctgacgtctaagaaagcttggtctcatggaattcgcggccgcttctagagtaatacgactcactatagggtattc<br>actgatcaaatcattcactccatcatagtctgatagatcttttgaagaactgattaatcggacgagaataaagagagagtc<br>ccattctacatgtcaatactgacaacaatgaaattactcgaacccctagcccgctcttatcgggcggctaggggtttttgtt<br>actagtagcggccgctgcagtgagaccggatccgctcactcaaaggcggtaatc

Sequence name	*Dalbergia pervillei* DpTrnL-UAA Trigger 1.3
Acc. number	BBa_K3453364
Sequence	ctgccacctgacgtctaagaaagcttggtctcatggaattcgcggccgcttctagagtaatacgactcactatagggtattc<br>actgatcaaatcattcaattcacttcatcatagtctgatagatcttttgaagaactgattaatcggacgagaataaagagag<br>agtcccattctacatgtcaatactgacaataattactcgaacccctagcccgctcttatcgggcggctaggggtttttgttac<br>tagtagcggccgctgcagtgagaccggatccgctcactcaaaggcggtaatc

Sequence name	*Millettia laurentii* MlTrnL-UAA Trigger 1.3
Acc. number	BBa_K3453365
Sequence	ctgccacctgacgtctaagaaagcttggtctcatggaattcgcggccgcttctagagtaatacgactcactatagggtattc<br>attgatcaaatcgtttactccattatagtctgatagatcctttgaagaactgattaatcagacgagaataaagatagagtcc<br>cattctacatgtcaataccgacaacaatgaaattactcgaacccctagcccgctcttatcgggcggctaggggtttttgtta<br>ctagtagcggccgctgcagtgagaccggatccgctcactcaaaggcggtaatc

Sequence name	Negative control (no promoter, no RBS):<br>Golden Gate (BsaI) adapter + sfGFP-LVAtag + SBa_000587 terminator
Acc. number	BBa_K3453101
Sequence	tgccacctgacgtctaagaaaaggaatattcagcaatttgcccgtgccgaagaaaggcccacccgtgaaggtgagcc<br>agtgagttgattgctacgtaattagttagttagcccttagtgactggaattcgcggccgcttctagagagagaccggatccg<br>gtctctatgcgtaaaggcgaagaactgtttaccggtgtggttccgattctggtggaactggacggcgatgttaatggtcata<br>aattcagtgttcgcggcgaaggtgaaggcgatgcgacgaacggcaaactgaccctgaaatttatctgcaccacgggta<br>aactgccggtcccgtggccgacgctggtgaccacgctgacctatggcgttcaatgttttgcgcgttacccggatcacatga<br>aacagcacgactttttcaaatcggccatgccggaaggctatgtgcaggaacgtacgattagctttaaagacgatggtac<br>gtataaaacccgcgcggaagtgaaattcgaaggcgataccctggttaaccgtatcgaactgaaaggtatcgatttcaaa<br>gaagatggcaatattctgggtcataaactggaatataacttcaattcccacaacgtgtacatcaccgcggataaacaga<br>aaaacggcattaaagccaatttcaaaatccgccataatgtggaagatggtagcgttcagctggccgaccactatcagc<br>aaaacacgccgattggtgatggcccggtcctgctgccggacaatcactacctgagtacccagtccgtgctgtcaaaag<br>atccgaacgaaaaacgtgaccacatggtcctgctggaatttgtgacggctgcgggtatcacccacggcatggacgaac<br>tgtataaaaggcctgctgcaaacgacgaaaactacgctttagtagcttaataatactcgaacccctagcccgctcttatcg<br>ggcggctaggggtttttgttactagtagcggccgctgcaggagtcactaagggttagttagttagattagcagaaagtca<br>aaagcctccgaccggaggcttttgactaaaacttcccttggggttatcattggggctcactcaaaggcggtaatcagata<br>aaaaaaatccttagctttcgctaaggatgatttctgctagagatggaatagactggatggaggcggataaagttgcagga<br>ccacttctgcgctcggcccttccggctggctggtttattgctgataaatctggagccggtgagcgtgggactcgcggtatca<br>ttgcagcactggggccagatggtaagccctcccgtatcgtagttatctacacgacggggagtcaggcaactatggatga<br>acgaaatagacagatcgctgagataggtgcctcactgattaagcattggtaactgtcagaccaagtttactcatatatactt<br>tagattgatttaaaacttcatttttaatttaaaaggatctaggtgaagatcctttttgataatctcatgaccaaaatcccttaacg<br>tgagttttcgttccactgagcgtcagaccccttaataagatgatcttcttgagatcgttttggtctgcgcgtaatctcttgctctga<br>aaacgaaaaaaccgccttgcagggcggtttttcgaaggttctctgagctaccaactctttgaaccgaggtaactggcttg<br>gaggagcgcagtcaccaaaacttgtcctttcagtttagccttaaccggcgcatgacttcaagactaactcctctaaatcaa<br>ttaccagtggctgctgccagtggtgcttttgcatgtctttccgggttggactcaagacgatagttaccggataaggcgcagc<br>ggtcggactgaacggggggttcgtgcatacagtccagcttggagcgaactgcctacccggaactgagtgtcaggcgtg<br>gaatgagacaaacgcggccataacagcggaatgacaccggtaaaccgaaaggcaggaacaggagagcgcacg<br>agggagccgccaggggaaacgcctggtatctttatagtcctgtcgggtttcgccaccactgatttgagcgtcagatttcgtg<br>atgcttgtcaggggggcggagcctatggaaaaacggctttgccgcggccctctcacttccctgttaagtatcttcctggcat<br>cttccaggaaatctccgccccgttcgtaagccatttccgctcgccgcagtcgaacgaccgagcgtagcgagtcagtgag<br>cgaggaagcggaatatatcctgtatcacatattctgctgacgcaccggtgcagcctttttctcctgccacatgaagcactt<br>cactgacaccctcatcagtgccaacatagtaagccagtatacactccgctagcgctgaggtctgcctcgtgaagaaggt<br>gttgctgactcataccaggcctgaatcgccccatcatccagccagaaagtgagggagccacggttgatgagagctttgtt<br>gtaggtggaccagttggtgattttgaacttttgctttgccacggaacggtctgcgttgtcgggaagatgcgtgatctgatcctt<br>caactcagcaaaagttcgatttattcaacaaagccacgttgtgtctcaaaatctctgatgttacattgcacaagataaaaat<br>atatcatcatgaacaataaaactgtctgcttacataaacagtaatacaaggggtgtttactagaggagattctcatgtttga<br>cagcttatcatcgataagctttaatgcggtagtttatcacagttaaattgctaacgcagtcaggcaccgtgtatgaaatctaa<br>caatgcgctcatcgtcattctcggcaccgtcaccctggacgctgtaggcataggcttggttatgccggtactgccgggcct<br>cttgcgggatatcgtccattccgacagtattgccagtcactatggcgtgctgcttgcgctctatgcgttgatgcaatttctttgc<br>gcacccgttctcggagccctgtccgaccgctttggccgccgtccagtcctgctcgcttcgctccttggagccactatcgact<br>acgcgatcatggcgaccacacccgtcctgtggattctctacgccggacgcatcgtggcgggcatcacgggtgccacag<br>gtgcggttgctggtgcctatatcgccgacatcaccgatggggaagatcgggctcgccacttcgggctcatgagcgcttgtt<br>tcggcgtgggtatggtggcaggccccgtggccgggggactgttgggtgccatctccttgcatgcaccattccttgcggcg<br>gcggtgctcaacggcctcaacctcctcctgggctgcttccttatgcaggaatcgcataagggagagcgccgtccgatgc<br>ccttgcgtgccttcaatccagtcagctccttccggtgggcgcggggcatgactatcgtcgccgcacttatgactgttttctttat<br>catgcaactcgtaggacaggttccggcagcgctctgggtcattttcggcgaggaccgctttcgctggagcgcgacgatg<br>atcggcctgtcgcttgcggtattcggaatcttgcacgccctcgctcaagccttcgtcacgggccccgccaccaaacgtttc<br>ggcgagaagcaggccattatcgcgggcatggcggccgacgcgctgggctacgtcttgctggcgttcgcgacgcgcgg<br>ctggatggccttccccattatgattcttctcgcttccggcggcatcggtatgcccgcgttgcaggccatgctgtcccgccaag<br>tagatgacgaccatcagggacagcttcaagggtcgctcgcggctcttaccagcctcacttcgatcattggaccgctgatc<br>gtcacggcgatttatgccgcctcggcgagcacatggaacgggttggcatggattgtaggtgccgccctttaccttgtctgc<br>ctccccgcgttgcgtcgcggtgcatggagccgggccacctcgacctaataatactagctccggcaaaaaaacgggca<br>aggtgtcaccaccctgcccttttttctttaaaaccgaaaagattacttcgcgtt

Sequence name	Negative control (no promoter, no RBS):<br>Golden Gate (BsmBI) adapter + sfGFP-LVAtag + SBa_000587 terminator
Acc. number	BBa_K3453102
Sequence	tgccacctgacgtctaagaaaaggaatattcagcaatttgcccgtgccgaagaaaggcccacccgtgaaggtgagcc<br>agtgagttgattgctacgtaattagttagttagcccttagtgactggaattcgcggccgcttctagagagagacgggatccc

	gtctctatgcgtaaaggcgaagaactgtttaccggtgtggttccgattctggtggaactggacggcgatgttaatggtcata<br>aattcagtgttcgcggcgaaggtgaaggcgatgcgacgaacggcaaactgaccctgaaatttatctgcaccacgggta<br>aactgccggtcccgtggccgacgctggtgaccacgctgacctatggcgttcaatgttttgcgcgttacccggatcacatga<br>aacagcacgactttttcaaatcggccatgccggaaggctatgtgcaggaacgtacgattagctttaaagacgatggtac<br>gtataaaacccgcgcggaagtgaaattcgaaggcgataccctggttaaccgtatcgaactgaaaggtatcgatttcaaa<br>gaagatggcaatattctgggtcataaactggaatataacttcaattcccacaacgtgtacatcaccgcggataaacaga<br>aaaacggcattaaagccaatttcaaaatccgccataatgtggaagatggtagcgttcagctggccgaccactatcagc<br>aaaacacgccgattggtgatggcccggtcctgctgccggacaatcactacctgagtacccagtccgtgctgtcaaaag<br>atccgaacgaaaaacgtgaccacatggtcctgctggaatttgtgacggctgcgggtatcacccacggcatggacgaac<br>tgtataaaaggcctgctgcaaacgacgaaaactacgctttagtagcttaataatactcgaacccctagcccgctcttatcg<br>ggcggctaggggtttttgttactagtagcggccgctgcaggagtcactaagggttagttagttagattagcagaaagtca<br>aaagcctccgaccggaggcttttgactaaaacttcccttggggttatcattggggctcactcaaaggcggtaatcagata<br>aaaaaaatccttagctttcgctaaggatgatttctgctagagatggaatagactggatggaggcggataaagttgcagga<br>ccacttctgcgctcggcccttccggctggctggtttattgctgataaatctggagccggtgagcgtgggactcgcggtatca<br>ttgcagcactggggccagatggtaagccctcccgtatcgtagttatctacacgacggggagtcaggcaactatggatga<br>acgaaatagacagatcgctgagataggtgcctcactgattaagcattggtaactgtcagaccaagtttactcatatatactt<br>tagattgatttaaaacttcatttttaatttaaaaggatctaggtgaagatcctttttgataatctcatgaccaaaatcccttaacg<br>tgagttttcgttccactgagcgtcagaccccttaataagatgatcttcttgagatcgttttggtctgcgcgtaatctcttgctctga<br>aaacgaaaaaaccgccttgcagggcggtttttcgaaggttctctgagctaccaactctttgaaccgaggtaactggcttg<br>gaggagcgcagtcaccaaaacttgtcctttcagtttagccttaaccggcgcatgacttcaagactaactcctctaaatcaa<br>ttaccagtggctgctgccagtggtgcttttgcatgtctttccgggttggactcaagacgatagttaccggataaggcgcagc<br>ggtcggactgaacggggggttcgtgcatacagtccagcttggagcgaactgcctacccggaactgagtgtcaggcgtg<br>gaatgagacaaacgcggccataacagcggaatgacaccggtaaaccgaaaggcaggaacaggagagcgcacg<br>agggagccgccaggggaaacgcctggtatctttatagtcctgtcgggtttcgccaccactgatttgagcgtcagatttcgtg<br>atgcttgtcaggggggcggagcctatggaaaaacggctttgccgcggccctctcacttccctgttaagtatcttcctggcat<br>cttccaggaaatctccgccccgttcgtaagccatttccgctcgccgcagtcgaacgaccgagcgtagcgagtcagtgag<br>cgaggaagcggaatatatcctgtatcacatattctgctgacgcaccggtgcagcctttttttctcctgccacatgaagcactt<br>cactgacaccctcatcagtgccaacatagtaagccagtatacactccgctagcgctgaggtctgcctcgtgaagaaggt<br>gttgctgactcataccaggcctgaatcgccccatcatccagccagaaagtgagggagccacggttgatgagagctttgtt<br>gtaggtggaccagttggtgattttgaacttttgctttgccacggaacggtctgcgttgtcgggaagatgcgtgatctgatcctt<br>caactcagcaaaagttcgatttattcaacaaagccacgttgtgtctcaaaatctctgatgttacattgcacaagataaaaat<br>atatcatcatgaacaataaaactgtctgcttacataaacagtaatacaaggggtgtttactagaggagattctcatgtttga<br>cagcttatcatcgataagctttaatgcggtagtttatcacagttaaattgctaacgcagtcaggcaccgtgtatgaaatctaa<br>caatgcgctcatcgtcattctcggcaccgtcaccctggacgctgtaggcataggcttggttatgccggtactgccgggcct<br>cttgcgggatatcgtccattccgacagtattgccagtcactatggcgtgctgcttgcgctctatgcgttgatgcaatttctttgc<br>gcacccgttctcggagccctgtccgaccgctttggccgccgtccagtcctgctcgcttcgctccttggagccactatcgact<br>acgcgatcatggcgaccacacccgtcctgtggattctctacgccggacgcatcgtggcgggcatcacgggtgccacag<br>gtgcggttgctggtgcctatatcgccgacatcaccgatggggaagatcgggctcgccacttcgggctcatgagcgcttgtt<br>tcggcgtgggtatggtggcaggccccgtggccgggggactgttgggtgccatctccttgcatgcaccattccttgcggcg<br>gcggtgctcaacggcctcaacctcctcctgggctgcttccttatgcaggaatcgcataagggagagcgccgtccgatgc<br>ccttgcgtgccttcaatccagtcagctccttccggtgggcgcggggcatgactatcgtcgccgcacttatgactgttttcttat<br>catgcaactcgtaggacaggttccggcagcgctctgggtcattttcggcgaggaccgctttcgctggagcgcgacgatg<br>atcggcctgtcgcttgcggtattcggaatcttgcacgccctcgctcaagccttcgtcacggccccgccaccaaacgtttc<br>ggcgagaagcaggccattatcgcgggcatggcggccgacgcgctgggctacgtcttgctggcgttcgcgacgcgcgg<br>ctggatggccttccccattatgattcttctcgcttccggcggcatcggtatgcccgcgttgcaggccatgctgtcccgccaag<br>tagatgacgaccatcagggacagcttcaagggtcgctcgcggctcttaccagcctcacttcgatcattggaccgctgatc<br>gtcacggcgatttatgccgcctcggcgagcacatggaacgggttggcatggattgtaggtgccgccctttaccttgtctgc<br>ctccccgcgttgcgtcgcggtgcatggagccgggccacctcgacctaataatactagctccggcaaaaaaacgggca<br>aggtgtcaccaccctgcccttttttctttaaaaccgaaaagattacttcgcgtt

Sequence name	Negative control (no promoter, no RBS):<br>Golden Gate (BbsI) adapter + sfGFP-LVAtag + SBa_000587 terminator
Acc. number	BBa_K3453103
Sequence	tgccacctgacgtctaagaaaaggaatattcagcaatttgcccgtgccgaagaaaggcccacccgtgaaggtgagcc<br>agtgagttgattgctacgtaattagttagttagcccttagtgactggaattcgcggccgcttctagagatgtcttcgcgcgaa<br>gacttatgcgtaaaggcgaagaactgtttaccggtgtggttccgattctggtggaactggacggcgatgttaatggtcata<br>aattcagtgttcgcggcgaaggtgaaggcgatgcgacgaacggcaaactgaccctgaaatttatctgcaccacgggta<br>aactgccggtcccgtggccgacgctggtgaccacgctgacctatggcgttcaatgttttgcgcgttacccggatcacatga<br>aacagcacgactttttcaaatcggccatgccggaaggctatgtgcaggaacgtacgattagctttaaagacgatggtac<br>gtataaaacccgcgcggaagtgaaattcgaaggcgataccctggttaaccgtatcgaactgaaaggtatcgatttcaaa

	gaagatggcaatattctgggtcataaactggaatataacttcaattcccacaacgtgtacatcaccgcggataaacaga<br>aaaacggcattaaagccaatttcaaaatccgccataatgtggaagatggtagcgttcagctggccgaccactatcagc<br>aaaacacgccgattggtgatggcccggtcctgctgccggacaatcactacctgagtacccagtccgtgctgtcaaaag<br>atccgaacgaaaaacgtgaccacatggtcctgctggaatttgtgacggctgcgggtatcacccacggcatggacgaac<br>tgtataaaggcctgctgcaaacgacgaaaactacgctttagtagcttaataatactcgaacccctagcccgctcttatcg<br>ggcggctaggggtttttgttactagtagcggccgctgcaggagtcactaagggttagttagttagattagcagaaagtca<br>aaagcctccgaccggaggcttttgactaaaacttcccttggggttatcattggggctcactcaaaggcggtaatcagata<br>aaaaaaatccttagctttcgctaaggatgatttctgctagagatggaatagactggatggaggcggataaagttgcagga<br>ccacttctgcgctcggcccttccggctggctggtttattgctgataaatctggagccggtgagcgtgggactcgcggtatca<br>ttgcagcactggggccagatggtaagccctcccgtatcgtagttatctacacgacggggagtcaggcaactatggatga<br>acgaaatagacagatcgctgagataggtgcctcactgattaagcattggtaactgtcagaccaagtttactcatatatactt<br>tagattgatttaaaacttcatttttaatttaaaaggatctaggtgaagatcctttttgataatctcatgaccaaaatcccttaacg<br>tgagttttcgttccactgagcgtcagaccccttaataagatgatcttcttgagatcgttttggtctgcgcgtaatctcttgctctga<br>aaacgaaaaaaccgccttgcagggcggtttttcgaaggttctctgagctaccaactctttgaaccgaggtaactggcttg<br>gaggagcgcagtcaccaaaacttgtcctttcagtttagccttaaccggcgcatgacttcaagactaactcctctaaatcaa<br>ttaccagtggctgctgccagtggtgcttttgcatgtctttccgggttggactcaagacgatagttaccggataaggcgcagc<br>ggtcggactgaacggggggttcgtgcatacagtccagcttggagcgaactgcctacccggaactgagtgtcaggcgtg<br>gaatgagacaaacgcggccataacagcggaatgacaccggtaaaccgaaaggcaggaacaggagagcgcacg<br>agggagccgccaggggaaacgcctggtatctttatagtcctgtcgggtttcgccaccactgatttgagcgtcagatttcgtg<br>atgcttgtcaggggggcggagcctatggaaaaacggctttgccgcggccctctcacttccctgttaagtatcttcctggcat<br>cttccaggaaatctccgccccgttcgtaagccatttccgctcgccgcagtcgaacgaccgagcgtagcgagtcagtgag<br>cgaggaagcggaatatatcctgtatcacatattctgctgacgcaccggtgcagcctttttttctcctgccacatgaagcactt<br>cactgacaccctcatcagtgccaacatagtaagccagtatacactccgctagcgctgaggtctgcctcgtgaagaaggt<br>gttgctgactcataccaggcctgaatcgccccatcatccagccagaaagtgagggagccacggttgatgagagctttgtt<br>gtaggtggaccagttggtgattttgaacttttgctttgccacggaacggtctgcgttgtcgggaagatgcgtgatctgatcctt<br>caactcagcaaaagttcgatttattcaacaaagccacgttgtgtctcaaaatctctgatgttacattgcacaagataaaaat<br>atatcatcatgaacaataaaactgtctgcttacataaacagtaatacaaggggtgtttactagaggagattctcatgtttga<br>cagcttatcatcgataagctttaatgcggtagtttatcacagttaaattgctaacgcagtcaggcaccgtgtatgaaatctaa<br>caatgcgctcatcgtcattctcggcaccgtcaccctggacgctgtaggcataggcttggttatgccggtactgccgggcct<br>cttgcgggatatcgtccattccgacagtattgccagtcactatggcgtgctgcttgcgctctatgcgttgatgcaatttctttgc<br>gcacccgttctcggagccctgtccgaccgctttggccgccgtccagtcctgctcgcttcgctccttggagccactatcgact<br>acgcgatcatggcgaccacacccgtcctgtggattctctacgccggacgcatcgtggcgggcatcacgggtgccacag<br>gtgcggttgctggtgcctatatcgccgacatcaccgatggggaagatcgggctcgccacttcgggctcatgagcgcttgtt<br>tcggcgtgggtatggtggcaggccccgtggccgggggactgttgggtgccatctccttgcatgcaccattccttgcggcg<br>gcggtgctcaacggcctcaacctcctcctgggctgcttcctaatgcaggaatcgcataagggagagcgccgtccgatgc<br>ccttgcgtgccttcaatccagtcagctccttccggtgggcgcggggcatgactatcgtcgccgcacttatgactgttttctttat<br>catgcaactcgtaggacaggttccggcagcgctctgggtcattttcggcgaggaccgctttcgctggagcgcgacgatg<br>atcggcctgtcgcttgcggtattcggaatcttgcacgccctcgctcaagccttcgtcacgggccccgccaccaaacgtttc<br>ggcgagaagcaggccattatcgcgggcatggcggccgacgcgctgggctacgtcttgctggcgttcgcgacgcgcgg<br>ctggatggccttccccattatgattcttctcgcttccggcggcatcggtatgcccgcgttgcaggccatgctgtcccgccaag<br>tagatgacgaccatcagggacagcttcaagggtcgctcgcggctcttaccagcctcacttcgatcattggaccgctgatc<br>gtcacggcgatttatgccgcctcggcgagcacatggaacgggttggcatggattgtaggtgccgccctttaccttgtctgc<br>ctccccgcgttgcgtcgcggtgcatggagccgggccacctcgacctaataatactagctccggcaaaaaaacgggca<br>aggtgtcaccaccctgccctttttctttaaaaccgaaaagattacttcgcgtt

Sequence name	Positive control (Custom made RBS):<br>sfGFP-LVAtag expression cassette under the control of the T7 promoter
Acc. number	BBa_K3453104
Sequence	tgccacctgacgtctaagaaaaggaatattcagcaatttgcccgtgccgaagaaaggcccacccgtgaaggtgagcc<br>agtgagttgattgctacgtaattagttagttagcccttagtgactggaattcgcggccgcttctagagtaatacgactcactat<br>agggcccgcccatacaaaataaggagcgttatttatgcgtaaaggcgaagaactgtttaccggtgtggttccgattctggt<br>ggaactggacggcgatgttaatggtcataaattcagtgttcgcggcgaaggtgaaggcgatgcgacgaacggcaaac<br>tgaccctgaaatttatctgcaccacgggtaaactgccggtcccgtggccgacgctggtgaccacgctgacctatggcgtt<br>caatgttttgcgcgttacccggatcacatgaaacagcacgactttttcaaatcggccatgccggaaggctatgtgcagga<br>acgtacgattagctttaaagacgatggtacgtataaaacccgcgcggaagtgaaattcgaaggcgataccctggttaac<br>cgtatcgaactgaaaggtatcgatttcaaagaagatggcaatattctgggtcataaactggaatataacttcaattcccac<br>aacgtgtacatcaccgcggataaacagaaaaacggcattaaagccaatttcaaaatccgccataatgtggaagatggt<br>agcgttcagctggccgaccactatcagcaaaacacgccgattggtgatggcccggtcctgctgccggacaatcactac<br>ctgagtacccagtccgtgctgtcaaaagatccgaacgaaaaacgtgaccacatggtcctgctggaatttgtgacggctg<br>cgggtatcacccacggcatggacgaactgtataaaggcctgctgcaaacgacgaaaactacgctttagtagcttaat

	aatactcgaacccctagcccgctcttatcgggcggctaggggttttttgttactagtagcggccgctgcaggagtcactaa<br>gggttagttagttagattagcagaaagtcaaaagcctccgaccggaggcttttgactaaaacttcccttggggttatcattg<br>gggctcactcaaaggcggtaatcagataaaaaaaatccttagctttcgctaaggatgatttctgctagagatggaataga<br>ctggatggaggcggataaagttgcaggaccacttctgcgctcggcccttccggctggctggtttattgctgataaatctgg<br>agccggtgagcgtgggactcgcggtatcattgcagcactggggccagatggtaagccctcccgtatcgtagttatctaca<br>cgacggggagtcaggcaactatggatgaacgaaatagacagatcgctgagataggtgcctcactgattaagcattggt<br>aactgtcagaccaagtttactcatatatactttagattgatttaaaacttcatttttaatttaaaaggatctaggtgaagatccttt<br>ttgataatctcatgaccaaaatcccttaacgtgagttttcgttccactgagcgtcagaccccttaataagatgatcttcttgag<br>atcgttttggtctgcgcgtaatctcttgctctgaaaacgaaaaaaccgccttgcagggcggtttttcgaaggttctctgagct<br>accaactctttgaaccgaggtaactggcttggaggagcgcagtcaccaaaacttgtcctttcagtttagccttaaccggcg<br>catgacttcaagactaactcctctaaatcaattaccagtggctgctgccagtggtgcttttgcatgtctttccgggttggactc<br>aagacgatagttaccggataaggcgcagcggtcggactgaacggggggttcgtgcatacagtccagcttggagcgaa<br>ctgcctacccggaactgagtgtcaggcgtggaatgagacaaacgcggccataacagcggaatgacaccggtaaacc<br>gaaaggcaggaacaggagagcgcacgagggagccgccaggggaaacgcctggtatctttatagtcctgtcgggtttc<br>gccaccactgatttgagcgtcagatttcgtgatgcttgtcaggggggcggagcctatggaaaaacggctttgccgcggcc<br>ctctcacttccctgttaagtatcttcctggcatcttccaggaaatctccgccccgttcgtaagccatttccgctcgccgcagtc<br>gaacgaccgagcgtagcgagtcagtgagcgaggaagcggaatatatcctgtatcacatattctgctgacgcaccggtg<br>cagcctttttctcctgccacatgaagcacttcactgacaccctcatcagtgccaacatagtaagccagtatacactccgct<br>agcgctgaggtctgcctcgtgaagaaggtgttgctgactcataccaggcctgaatcgccccatcatccagccagaaagt<br>gagggagccacggttgatgagagctttgttgtaggtggaccagttggtgattttgaacttttgctttgccacggaacggtctg<br>cgttgtcgggaagatgcgtgatctgatccttcaactcagcaaaagttcgatttattcaacaaagccacgttgtgtctcaaaa<br>tctctgatgttacattgcacaagataaaaatatatcatcatgaacaataaaactgtctgcttacataaacagtaatacaag<br>gggtgtttactagaggagattctcatgtttgacagcttatcatcgataagctttaatgcggtagtttatcacagttaaattgcta<br>acgcagtcaggcaccgtgtatgaaatctaacaatgcgctcatcgtcattctcggcaccgtcaccctggacgctgtaggca<br>taggcttggttatgccggtactgccgggcctcttgcgggatatcgtccattccgacagtattgccagtcactatggcgtgctg<br>cttgcgctctatgcgttgatgcaatttctttgcgcacccgttctcggagccctgtccgaccgctttggccgccgtccagtcctg<br>ctcgcttcgctccttggagccactatcgactacgcgatcatggcgaccacacccgtcctgtggattctctacgccggacgc<br>atcgtggcgggcatcacgggtgccacaggtgcggttgctggtgcctatatcgccgacatcaccgatggggaagatcgg<br>gctcgccacttcgggctcatgagcgcttgtttcggcgtgggtatggtggcaggccccgtggccgggggactgttgggtgc<br>catctccttgcatgcaccattccttgcggcggcggtgctcaacggcctcaacctcctcctgggctgcttcctaatgcaggaat<br>cgcataagggagagcgccgtccgatgcccttgcgtgccttcaatccagtcagctccttccggtgggcgcggggcatgac<br>tatcgtcgccgcacttatgactgttttctttatcatgcaactcgtaggacaggttccggcagcgctctgggtcattttcggcga<br>ggaccgctttcgctggagcgcgacgatgatcggcctgtcgcttgcggtattcggaatcttgcacgccctcgctcaagcctt<br>cgtcacgggccccgccaccaaacgtttcggcgagaagcaggccattatcgcgggcatggcggccgacgcgctgggc<br>tacgtcttgctggcgttcgcgacgcgcggctggatggccttccccattatgattcttctcgcttccggcggcatcggtatgcc<br>cgcgttgcaggccatgctgtcccgccaagtagatgacgaccatcagggacagcttcaagggtcgctcgcggctcttacc<br>agcctcacttcgatcattggaccgctgatcgtcacggcgatttatgccgcctcggcgagcacatggaacgggttggcatg<br>gattgtaggtgccgccctttaccttgtctgcctccccgcgttgcgtcgcggtgcatggagccgggccacctcgacctaata<br>atactagctccggcaaaaaaacgggcaaggtgtcaccaccctgccctttttctttaaaaccgaaaagattacttcgcgtt

Sequence name	Positive control (Synthetic RBS stem-loop):<br>sfGFP-LVAtag expression cassette under the control of the T7 promoter
Acc. number	BBa_K3453105
Sequence	tgccacctgacgtctaagaaaaggaatattcagcaatttgcccgtgccgaagaaaggcccacccgtgaaggtgagcc<br>agtgagttgattgctacgtaattagttagttagcccttagtgactggaattcgcggccgcttctagagtaatacgactcactat<br>agggggactttagaacagaggagataaagatgcgtaaaggcgaagaactgtttaccggtgtggttccgattctggtgga<br>actggacggcgatgttaatggtcataaattcagtgttcgcggcgaaggtgaaggcgatgcgacgaacggcaaactgac<br>cctgaaatttatctgcaccacgggtaaactgccggtcccgtggccgacgctggtgaccacgctgacctatggcgttcaat<br>gttttgcgcgttacccggatcacatgaaacagcacgactttttcaaatcggccatgccggaaggctatgtgcaggaacgt<br>acgattagctttaaagacgatggtacgtataaaacccgcgcggaagtgaaattcgaaggcgataccctggttaaccgta<br>tcgaactgaaaggtatcgatttcaaagaagatggcaatattctgggtcataaactggaatataacttcaattcccacaacg<br>tgtacatcaccgcggataaacagaaaaacggcattaaagccaatttcaaaatccgccataatgtggaagatggtagcg<br>ttcagctggccgaccactatcagcaaaacacgccgattggtgatggcccggtcctgctgccggacaatcactacctgag<br>tacccagtccgtgctgtcaaaagatccgaacgaaaaacgtgaccacatggtcctgctggaatttgtgacggctgcgggt<br>atcacccacggcatggacgaactgtataaaggcctgctgcaaacgacgaaaactacgctttagtagcttaataatact<br>cgaacccctagcccgctcttatcgggcggctaggggttttttgttactagtagcggccgctgcaggagtcactaagggtta<br>gttagttagattagcagaaagtcaaaagcctccgaccggaggcttttgactaaaacttcccttggggttatcattggggctc<br>actcaaaggcggtaatcagataaaaaaaatccttagctttcgctaaggatgatttctgctagagatggaatagactggat<br>ggaggcggataaagttgcaggaccacttctgcgctcggcccttccggctggctggtttattgctgataaatctggagccgg<br>tgagcgtgggactcgcggtatcattgcagcactggggccagatggtaagccctcccgtatcgtagttatctacacgacgg

	ggagtcaggcaactatggatgaacgaaatagacagatcgctgagataggtgcctcactgattaagcattggtaactgtc<br>agaccaagtttactcatatatactttagattgatttaaaacttcatttttaatttaaaaggatctaggtgaagatcctttttgataat<br>ctcatgaccaaaatcccttaacgtgagttttcgttccactgagcgtcagaccccttaataagatgatcttcttgagatcgttttg<br>gtctgcgcgtaatctcttgctctgaaaacgaaaaaaccgccttgcagggcggtttttcgaaggttctctgagctaccaactc<br>tttgaaccgaggtaactggcttggaggagcgcagtcaccaaaacttgtcctttcagtttagccttaaccggcgcatgacttc<br>aagactaactcctctaaatcaattaccagtggctgctgccagtggtgcttttgcatgtctttccgggttggactcaagacgat<br>agttaccggataaggcgcagcggtcggactgaacggggggttcgtgcatacagtccagcttggagcgaactgcctacc<br>cggaactgagtgtcaggcgtggaatgagacaaacgcggccataacagcggaatgacaccggtaaaccgaaaggc<br>aggaacaggagagcgcacgagggagccgccaggggaaacgcctggtatctttatagtcctgtcgggtttcgccacca<br>ctgatttgagcgtcagatttcgtgatgcttgtcaggggggcggagcctatggaaaaacggctttgccgcggccctctcactt<br>ccctgttaagtatcttcctggcatcttccaggaaatctccgccccgttcgtaagccatttccgctcgccgcagtcgaacgac<br>cgagcgtagcgagtcagtgagcgaggaagcggaatatatcctgtatcacatattctgctgacgcaccggtgcagcctttt<br>ttctcctgccacatgaagcacttcactgacaccctcatcagtgccaacatagtaagccagtatacactccgctagcgctg<br>aggtctgcctcgtgaagaaggtgttgctgactcataccaggcctgaatcgccccatcatccagccagaaagtgaggga<br>gccacggttgatgagagctttgttgtaggtggaccagttggtgattttgaacttttgctttgccacggaacggtctgcgttgtcg<br>ggaagatgcgtgatctgatccttcaactcagcaaaagttcgatttattcaacaaagccacgttgtgtctcaaaatctctgat<br>gttacattgcacaagataaaaatatatcatcatgaacaataaaactgtctgcttacataaacagtaatacaaggggtgttt<br>actagaggagattctcatgtttgacagcttatcatcgataagctttaatgcggtagtttatcacagttaaattgctaacgcagt<br>caggcaccgtgtatgaaatctaacaatgcgctcatcgtcattctcggcaccgtcaccctggacgctgtaggcataggctt<br>ggttatgccggtactgccgggcctcttgcgggatatcgtccattccgacagtattgccagtcactatggcgtgctgcttgcg<br>ctctatgcgttgatgcaatttctttgcgcacccgttctcggagccctgtccgaccgctttggccgccgtccagtcctgctcgctt<br>cgctccttggagccactatcgactacgcgatcatggcgaccacacccgtcctgtggattctctacgccggacgcatcgtg<br>gcgggcatcacgggtgccacaggtgcggttgctggtgcctatatcgccgacatcaccgatggggaagatcgggctcgc<br>cacttcgggctcatgagcgcttgtttcggcgtgggtatggtggcaggccccgtggccgggggactgttgggtgccatctcc<br>ttgcatgcaccattccttgcggcggcggtgctcaacggcctcaacctcctcctgggctgcttccttatgcaggaatcgcata<br>agggagagcgccgtccgatgcccttgcgtgccttcaatccagtcagctccttccggtgggcgcggggcatgactatcgtc<br>gccgcacttatgactgttttctttatcatgcaactcgtaggacaggttccggcagcgctctgggtcattttcggcgaggaccg<br>ctttcgctggagcgcgacgatgatcggcctgtcgcttgcggtattcggaatcttgcacgccctcgctcaagccttcgtcacg<br>ggccccgccaccaaacgtttcggcgagaagcaggccattatcgcgggcatggcggccgacgcgctgggctacgtctt<br>gctggcgttcgcgacgcgcggctggatggccttccccattatgattcttctcgcttccggcggcatcggtatgcccgcgttg<br>caggccatgctgtcccgccaagtagatgacgaccatcagggacagcttcaagggtcgctcgcggctcttaccagcctc<br>acttcgatcattggaccgctgatcgtcacggcgatttatgccgcctcggcgagcacatggaacgggttggcatggattgta<br>ggtgccgccctttaccttgtctgcctccccgcgttgcgtcgcggtgcatggagccgggccacctcgacctaataatactag<br>ctccggcaaaaaaacgggcaaggtgtcaccaccctgcccttttctttaaaaccgaaaagattacttcgcgtt

Supplementary References

[1] Hong Z, Li J, Liu X, Lian J, Zhang N, Yang Z, et al. The chromosome-level draft genome of Dalbergia odorifera. Gigascience 2020;9. https://doi.org/10.1093/gigascience/giaa084.

[2] Song Y, Zhang Y, Xu J, Li W, Li M. Characterization of the complete chloroplast genome sequence of Dalbergia species and its phylogenetic implications. Sci Rep 2019;9:20401. https://doi.org/10.1038/s41598-019-56727-x.

[3] Liu Y, Huang P, Li C-H, Zang F-Q, Zheng Y-Q. Characterization of the complete chloroplast genome of Dalbergia cultrata (Leguminosae). Mitochondrial DNA B Resour 2019;4:2369–70. https://doi.org/10.1080/23802359.2019.1631131.

[4] Hassold S, Lowry PP, Bauert MR, Razafintsalama A, Ramamonjisoa L, Widmer A. DNA barcoding of Malagasy rosewoods: towards a molecular identification of CITES-listed Dalbergia species. PLoS One 2016;11:e0157881. https://doi.org/10.1371/journal.pone.0157881.

[5] CBOL Plant Working Group. A DNA barcode for land plants. Proc Natl Acad Sci U S A 2009;106:12794–7. https://doi.org/10.1073/pnas.0905845106.

[6] Ratsimbazafy C, Newton DJ, Ringuet S. Timber island: rosewood and ebony trade of Madagascar. 2016.

[7] Pardee K, Green AA, Takahashi MK, Braff D, Lambert G, Lee JW, et al. Rapid, low-cost detection of Zika virus using programmable biomolecular components. Cell 2016;165:1255–66. https://doi.org/10.1016/j.cell.2016.04.059.

[8] Huang A, Nguyen PQ, Stark JC, Takahashi MK, Donghia N, Ferrante T, et al. BioBits™ Explorer: A modular synthetic biology education kit. Science Advances 2018;4:eaat5105. https://doi.org/10.1126/sciadv.aat5105.

[9] To AC-Y, Chu DH-T, Wang AR, Li FC-Y, Chiu AW-O, Gao DY, et al. A comprehensive web tool for toehold switch design. Bioinformatics 2018;34:2862–4. https://doi.org/10.1093/bioinformatics/bty216.

[10] Zadeh JN, Steenberg CD, Bois JS, Wolfe BR, Pierce MB, Khan AR, et al. NUPACK: Analysis and design of nucleic acid systems. J Comput Chem 2011;32:170–3. https://doi.org/10.1002/jcc.21596.

[11] Kerpedjiev P, Hammer S, Hofacker IL. Forna (force-directed RNA): Simple and effective online RNA secondary structure diagrams. Bioinformatics 2015;31:3377–9. https://doi.org/10.1093/bioinformatics/btv372.

[12] Engler C, Kandzia R, Marillonnet S. A one pot, one step, precision cloning method with high throughput capability. PLoS ONE 2008;3:e3647. https://doi.org/10.1371/journal.pone.0003647.

[13] Engler C, Gruetzner R, Kandzia R, Marillonnet S. Golden gate shuffling: a one-pot DNA shuffling method based on type IIs restriction enzymes. PLoS ONE 2009;4:e5553. https://doi.org/10.1371/journal.pone.0005553.
